# Jail, an unappreciated medical home: Assessing the feasibility of a strengths-based case management intervention to improve the care retention of HIV-infected persons once released from jail

**DOI:** 10.1371/journal.pone.0191643

**Published:** 2018-03-30

**Authors:** Anne C. Spaulding, Ana Drobeniuc, Paula M. Frew, Tiffany L. Lemon, Emeli J. Anderson, Colin Cerwonka, Chava Bowden, John Freshley, Carlos del Rio

**Affiliations:** 1 Department of Medicine, Division of Infectious Diseases, Emory University School of Medicine, Atlanta, Georgia, United States of America; 2 Department of Epidemiology, Rollins School of Public Health, Emory University, Atlanta, Georgia, United States of America; 3 Department of Behavioral Sciences and Health Education, Rollins School of Public Health, Emory University, Atlanta, Georgia, United States of America; 4 Hubert Department of Global Health, Rollins School of Public Health, Emory University, Atlanta, Georgia, United States of America; NYC Department of Health and Mental Hygiene, UNITED STATES

## Abstract

**Background:**

Linkage to and retention in care for US persons living with HIV (PLWH) after release from jail usually declines. We know of no rigorously evaluated behavioral interventions that can improve this. We hypothesized that a strengths-based case management intervention that we developed for PLWH leaving jail would increase linkage/retention in care (indicated by receipt of laboratory draws) and a suppressed HIV viral load (VL) in the year following release.

**Methods and findings:**

We conducted a quasi-experimental feasibility study of our intervention for PLWH jailed in Atlanta. We recruited 113 PLWH in jail starting in 2014. “SUCCESS” (Sustained, Unbroken Connection to Care, Entry Services, and Suppression) began in jail and continued post-release. Subjects who started the intervention but subsequently began long-term incarcerations were excluded from further analysis. Persons who were retained in the intervention group were compared to contemporaneously incarcerated PLWH who did not receive the intervention. Identities were submitted to an enhanced HIV/AIDS reporting system (eHARS) at the state health department to capture all laboratories drawn. Both community engagement and care upon jail return were assessed equally. For 44 intervention participants released to Atlanta, 50% of care occurred on subsequent jail stays, as documented with EventFlow software. Forty-five receiving usual services only were recruited for comparison. By examining records of jail reentries, half of participants and 60% of controls recidivated (range: 1–8 returns). All but 6 participants in the intervention and 9 subjects in the comparison arm had ≥1 laboratory recorded in eHARS post-release. Among the intervention group, 52% were retained in care (i.e., had two laboratory studies, > = 3 months apart), versus 40% among the comparison group (OR = 1.60, 95% CI (0.71, 3.81)). Both arms showed improved viral load suppression.

**Conclusions:**

There was a trend towards increased retention for PLWH released from jail after SUCCESS, compared to usual services. Measuring linkage at all venues, including jail-based clinics, fully captured engagement for this frequently recidivating population.

**Trial registration:**

ClinicalTrials.gov NCT02185742

## Introduction

Staying connected with HIV care is challenging for most infected persons. In the United States, with the world’s highest incarceration rate,[1] 150,000 persons with HIV come in contact with the criminal justice system each year,[2] which further complicates linkage to and retention in care. Most HIV-infected persons in jails and prisons were infected and diagnosed prior to their current incarceration.[3, 4] A recent meta-analysis showed that 76% of persons living with HIV (PLWH) entering American correctional facilities were linked to and retained in care while incarcerated.[5] Viral suppression is also achievable in the structured environment of a correctional facility, but post-release, the percentage of persons in each step of the care continuum plummets to rates much lower than before entry—the meta-analysis estimated that nationally only 30% of persons who are released are retained in care.[5, 6]

Of the 150,000 HIV+ persons released annually from U.S. correctional facilities, 95% leave jails, which are short-term, high throughput correctional facilities usually run by counties or cities for those who are awaiting trial or serving brief sentences.[2] Results from a study in Baltimore suggest that a short jail stay can disrupt antiretroviral therapy much more than a prison sentence.[7] A jail can release a detained person based on a sudden decision, such as the court system dropping charges. During this unplanned event, health workers may not give the patient sufficient medications to tide him or her over until arranging a visit to a community clinic. A recent study in New York City showed sporadic, brief occurrences of imprisonment or prolonged jail stays were particularly disruptive to maintaining a suppressed viral load, as indicated by laboratory results recorded in its comprehensive state HIV registry.[8] Since undiagnosed, and diagnosed persons out of care, are the source of 9 out of 10 new infections,[9] current efforts to expand HIV testing and treatment in jails must be coordinated with efforts to link or re-engage persons into community care after discharge.[10]

We conducted a quasi-experimental feasibility study of an intervention of strengths-based case management for HIV-infected jailed individuals, to increase linkage and retention in HIV care post-release. The intervention, SUCCESS (Sustained, Unbroken Connection to Care, Entry Services, and Suppression), is an enhanced version of the intervention used in the Antiretroviral Treatment and Access Study (ARTAS).[11] In this paper, we explore the trajectory of participants in the year following release from jail. The primary outcomes of the study are 1) retention in HIV care and 2) whether the last known HIV viral load was suppressed (≤200 copies/ml. We hypothesize that those receiving the intervention linked and stayed linked to care at rates higher than those who did not receive the intervention.

## Methods

### Recruitment

We recruited participants from Fulton County Jail (FCJ) in Atlanta, Georgia, a 2500-bed mega-jail, where HIV population prevalence has been rising from 2–3% in 2011[12] to almost 5% in 2017 (range 100–130 infected persons/range 2500–2600 total population). Fulton County is one of two counties that make up Atlanta; the eastern 10% of the city is in DeKalb County. The three jails serving the city are Fulton and DeKalb County Jails, and the Atlanta City Detention Center. We recruited study participants to intervention and comparison groups, via jail healthcare staff referrals, in alternating months from August 2014 to February 2015, to demonstrate the feasibility of accruing study participants at a rate that would support an adequately powered randomized trial in the future. During the first month and subsequent odd months, we recruited participants for the intervention; we recruited an equal number of participants in month 2 (and each subsequent even month) for the comparison group. We sought participants who were HIV infected, aged over 18 years, and likely to leave jail within 6 weeks and settle in Atlanta. Those in the comparison group received usual care, involving minimal discharge planning provided by jail staff, such as provision of a list of local HIV clinics and social service agencies, and were passively observed. We enrolled persons in the comparison group with the intention of having gender and age balanced with intervention participants. Both gender and age are associated with medication adherence and both can be derived from administrative data; in contrast, to measure other variables predicting adherence requires administering psychosocial surveys.

Both those recruited in the intervention group and the comparison group needed to be English-speaking and able to demonstrate literacy. We assessed literacy and therefore ability to conduct the texting portion of the intervention with the Rapid Estimate of Adult Literacy in Medicine (REALM) test.[13] In addition, those in the intervention group needed to be willing to use a cell phone for text messaging during the study. Those eligible for participation in the study underwent the informed consent process. Those in the intervention group were informed that the SUCCESS discharge planning process would be stopped if they subsequently started a long-term sentence. Participants in the intervention group also signed individual releases of information for each site of future care that they identified, and completed a baseline audio-computer assisted survey instrument (ACASI) survey to assess risk behaviors, access to care, and additional health-related information.

All study participants provided written consent for us to follow their clinical laboratory results during the index incarceration and in the year following initial release. The source of these laboratory results was two-fold. For both intervention and comparison groups, aggregate data on linkage, retention, and viral suppression status was collected from the Georgia State Department of Public Health (GDPH) enhanced HIV/AIDS reporting system (eHARS), to which all commercial laboratories operating in the state, including those serving the local jails, submit viral loads. For those in the intervention group only, the study team collected individual medical records at sites of HIV care previously identified through participant interviews.

The present analysis focuses on study participants released from jail directly to the greater Atlanta metropolitan area. We excluded persons who transferred to another correctional facility such as the state prison, relocated to an area beyond 85 miles from Atlanta, or who remained in jail beyond six months after recruitment closed.

### Intervention

Interventionists delivered six sessions of strengths-based case management via face-to-face sessions, beginning in jail and continuing in the community. Our plan was to deliver the initial session and up to one additional session of the intervention in the jail; the remaining 4 to 5 sessions were planned for delivery in the community, such as in the waiting rooms of HIV clinic offices or other locations convenient for the participants. Case managers used phone texting technology through Dimagi CommCare to improve participants’ connections to care in the community.[14] This technology could send appointment reminders, follow-up satisfaction questionnaires, and log case management sessions. In addition to the automated text service, intervention participants communicated with case managers via free-form text.

### Measurements, data collection and analysis

We searched publicly available custody entry logs of Fulton and DeKalb County Jails, and Georgia Department of Corrections to determine if the intervention or comparison participants were in custody at any point during the year after their initial release from Fulton County Jail. Atlanta City Detention Center responded to our request for similar data. For individual outcome data among intervention participants, we sought clinical data in medical records, both in the community, or if they returned to jail, in the jail medical clinic. In total, individual medical records for intervention participants were collected from 3 jail sites, including FCJ, and 3 community clinic sites. Measurements included whether (1) they had linkage, defined as any HIV-associated laboratory studies (CD4 cell count or viral load, VL) during the first year after their initial discharge from jail, (2) more than one test 3 months apart, which we called “retention”, and (3) among those with laboratory studies, if their last known VL within one year of their initial release date was suppressed. In addition to individual-level outcome data collection among intervention participants only, we submitted names, known aliases, and dates of birth to the GDPH eHARS to receive outcome data in the aggregate for both groups.

We used EventFlow software (College Park, MD; http://hcil.umd.edu/eventflow/),[15] a visualization tool to study temporal events and interval sequences in large datasets, to compare jail readmission events between the participant and comparison groups. For those in the intervention group, we used EventFlow to map the sequence of HIV care visits collected by the study team and the administration of follow-up surveys, in addition to jail re-entries. [16] Given that laboratory records were only collected in the aggregate for the comparison group, we were not able to include a temporal visualization of their HIV care visits.

Demographic and baseline HIV-related medical data were collected for all participants from jail custody and medical records. We compared differences in age, gender, and laboratories between the two groups, using Chi-square and t-tests. In addition, intervention participants self-reported information on sexual orientation, housing, health insurance status, previous jail stays, new diagnosis status, and HIV medical adherence on their baseline ACASI surveys. We also captured data on HIV risk behavior and gender of sexual partners. The Texas Christian University Drug Screen II (TCUDS II),[17] WHO Alcohol Use Disorders Identification Test (AUDIT),[18, 19] and the Center for Epidemiology Studies and Depression Scale 10 (CES-D 10)[20] were used to assess substance use and mental health on baseline and follow-up surveys. Drug use was dichotomized, with a score of 3 or greater on the TCUDS indicating relatively severe drug-related problems. Depression was dichotomized with a score of 10 or greater on the CES-D assessment indicating depression. Alcohol use was dichotomized with a score of greater than or equal to 8 on the AUDIT assessment indicating risky or hazardous level drinking habits. We compiled all data in the online database, REDCap.[21] All variables analyzed for associations were dichotomized at means or values suggested by aforementioned literature.

We conducted a bivariate analysis to explore associations of interest between an HIV-1 viral load ≤ 200 copies/mL (suppressed) at baseline and demographic and follow-up data. Aggregate data on linkage and retention in HIV care were compared between intervention and comparison groups using the Chi-Square test of significance. We also used bivariate analysis to calculate the odds ratio and 95% confidence intervals for the following two end-of-study outcomes: 1) retention in care and 2) a suppressed viral load at last known blood draw in the year following release. Significance was assessed at an alpha level of 0.05 using Chi-Square and Fisher’s Exact Tests. All analyses were performed using SAS 9.4 software (Cary NC).

### Human subjects protection

The Emory University Institutional Review Board approved the study (IRB00064852). It was registered in clinicaltrials.gov (NCT02185742).

## Results

**Fig 1:**
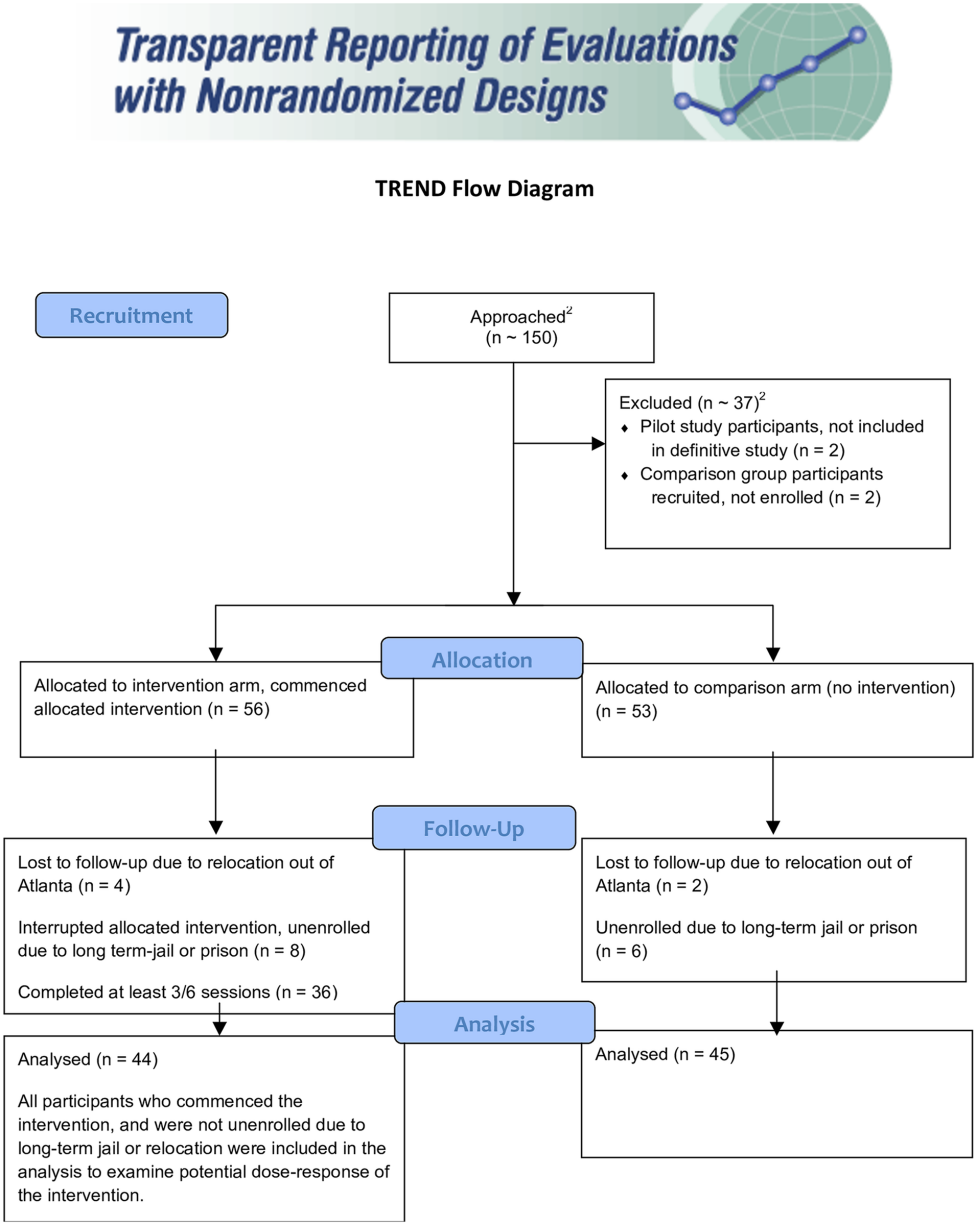
Participant flow diagram.

Participants were recruited at a pace of 14 enrollees per month. A total of 56 persons were assigned to the intervention arm and 53 to the comparison arm of the study. Eight intervention group members and 6 comparison group members began long term stays in either jail or prison, and 4 intervention and 2 comparison group members transferred out of the area. A total of 44 intervention recipients and 45 comparison persons had data on viral load and HIV care retention over the course of 1 year following initial release from jail and were included in the final study (Fig 1). Self-reported survey data were collected at 3 months and 12 months from intervention participants only.

Demographic, medical, and sociodemographic characteristics are detailed for study participants in Table 1. The typical recipient of the intervention was 37 years old, Black, a man who had sex with men (MSM) and already owned a phone. The average length of initial jail stay was 55.5 days and 77.9 days in the intervention group and comparison group, respectively. Participants in the intervention group completed an average of 4.3 case management sessions, and approximately half of participants engaged in some form of two-way texting with the case manager or with the automated messaging system.

**Table 1:** Demographic, psychological, and medical characteristics of SUCCESS^1^ study participants^2^, Atlanta, GA, 2014–2015 (N = 89).

Variable	Category	Intervention Group (n = 44)	Comparison Group (n = 45)	
		Mean (SD) or n (%)	Mean (SD) or n (%)	*p- value*^3^
**Age, years**		36.9 (8.1)	40.5 (9.7)	0.06
**Gender**	Male	38 (86.4)	41 (91.1)	1.00
	Female	4 (9.0)	4 (8.9)	
	Transgender, M to F	2 (4.5)	0	
**Race**	Black, or Black and Other	41 (93.2)	40 (88.9)	0.71
	Non-Black	3 (6.8)	5 (11.1)	
**Self-reported Sexual Orientation**^4^	Not Heterosexual	32 (72.7)	N/A	
**Employment**	Unemployed/Disabled	38 (84.4)	N/A -	
**Education**	Less than HS Grad, or no GED	12 (27.3)	-	
**Homeless 30 days prior to jail or Unstably Housed**^5^	Yes	38 (84.4)	-	
**Past year jail stays**	Yes	24 (54.5)	26 (57.8)	
**Mean length of index stay (i.e. stay when recruited)**		55.5 days (58.6) Median = 30.5	77.9 days (119.0) Median = 42.0	0.28
**First diagnosed on index jail stay**^6^		4 (8.9)	1 (2.2%)	0.16
**Depression (CES-D 10) (≥10 indicates depression)**	≥ 10	36 (81.8)	-	
**Alcohol (AUDIT) (≥8 indicates moderate risk)**	≥ 8	13 (30.0)	-	
**Drug Use (TCUDS) (≥3 indicates relatively severe drug problems)**	≥ 3	19 (43.2)	-	
**Ever prescribed anti-HIV medication**	Yes	30 (68.2)	-	
**HIV Medication Adherence (30 days prior to jail)**	Less than 50% 50% or more Not prescribed	16 (36.4) 14 (31.8) 14 (31.8)	-	-
**Viral Load (1 had no baseline viral load due to insufficient sample**	≤ 200 copies/ml	7 (15.9)	13 (28.9)	0.46
	>200 copies/ml	36 (81.8)	32 (71.7)	
**CD4 Count**	Less than 500 cells/mm^3^	31 (70.5)	31 (68.9)	1.00

^1^ Sustained, Unbroken, Connection to Care, Entry Services, and Suppression; strengths-based case management intervention implemented among individuals released from Fulton County Jail, compared with usual jail discharge services.

^2^ Demographic and medical measures collected from jail custody and medical records for both intervention and comparison groups. Self-reported risk behavior data collected from intervention group only.

^3^ Differences were not found to be statistically significant between the two groups, using Chi-square and t-tests.

^4^ 1 missing, refused to respond

^5^ Unstably housed defined as not owning or renting a home in the 30 days prior to jail

^6^ 6 self-reported new diagnoses, 2 had previous HIV test results in our records, 4 were confirmed as new. Data on newness of diagnosis for participant and comparison groups from jail records, confirmed with state of Georgia HIV registry.

**Fig 2:**
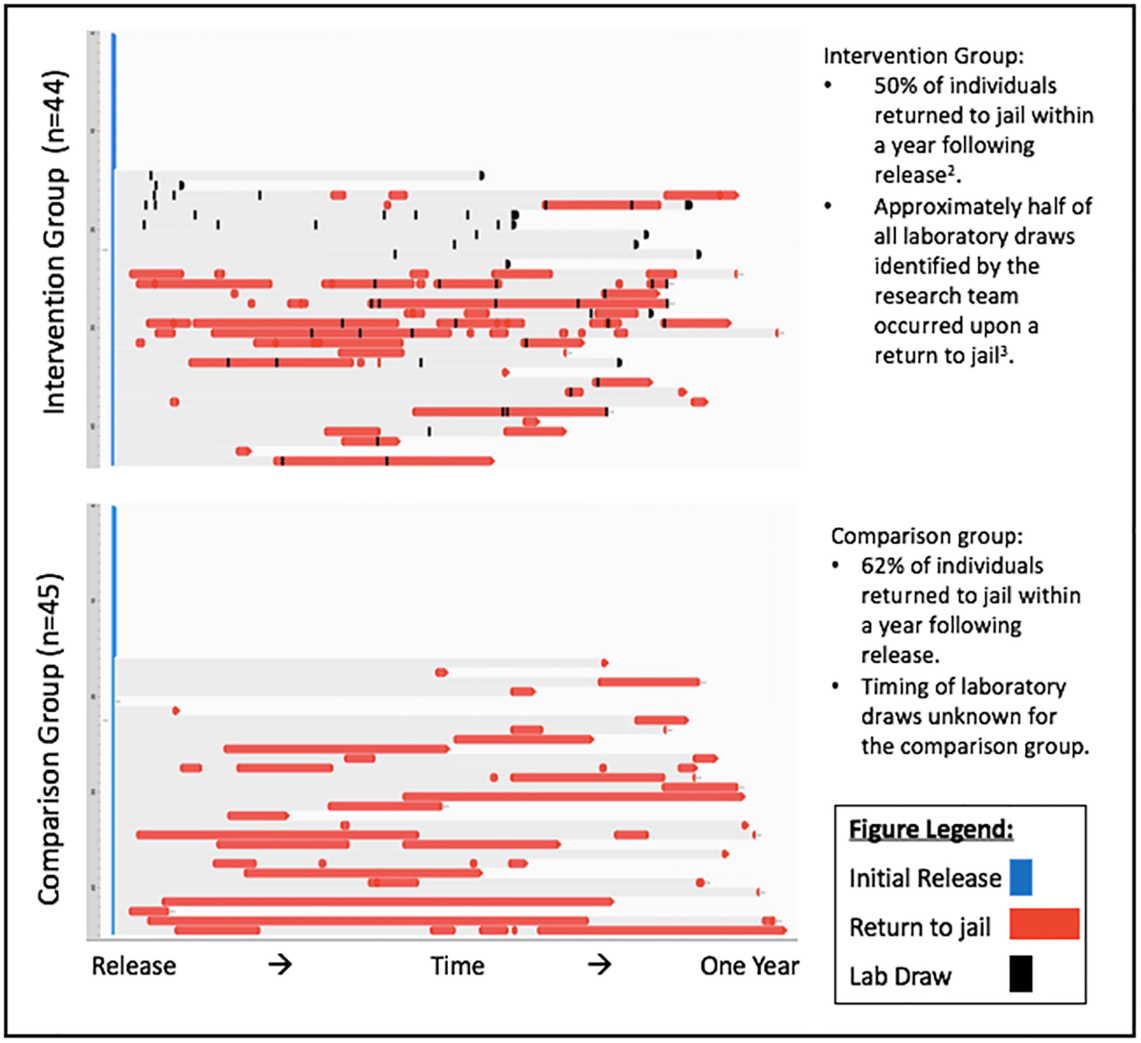
Trajectory of HIV care and recidivism events for SUCCESS^1^ study participants after index jail stay, Fulton County Jail, Atlanta, GA, 2014–2016. • In each panel, the horizontal axis displays time since initial release. • Each subject has a row entry, with red intervals representing re-entries to an area jail after the initial release from the enrollment jail stay. • Laboratory data were not collected by the research team on an individual basis for the comparison group, and are thus not reflected.

**Fig 3:**
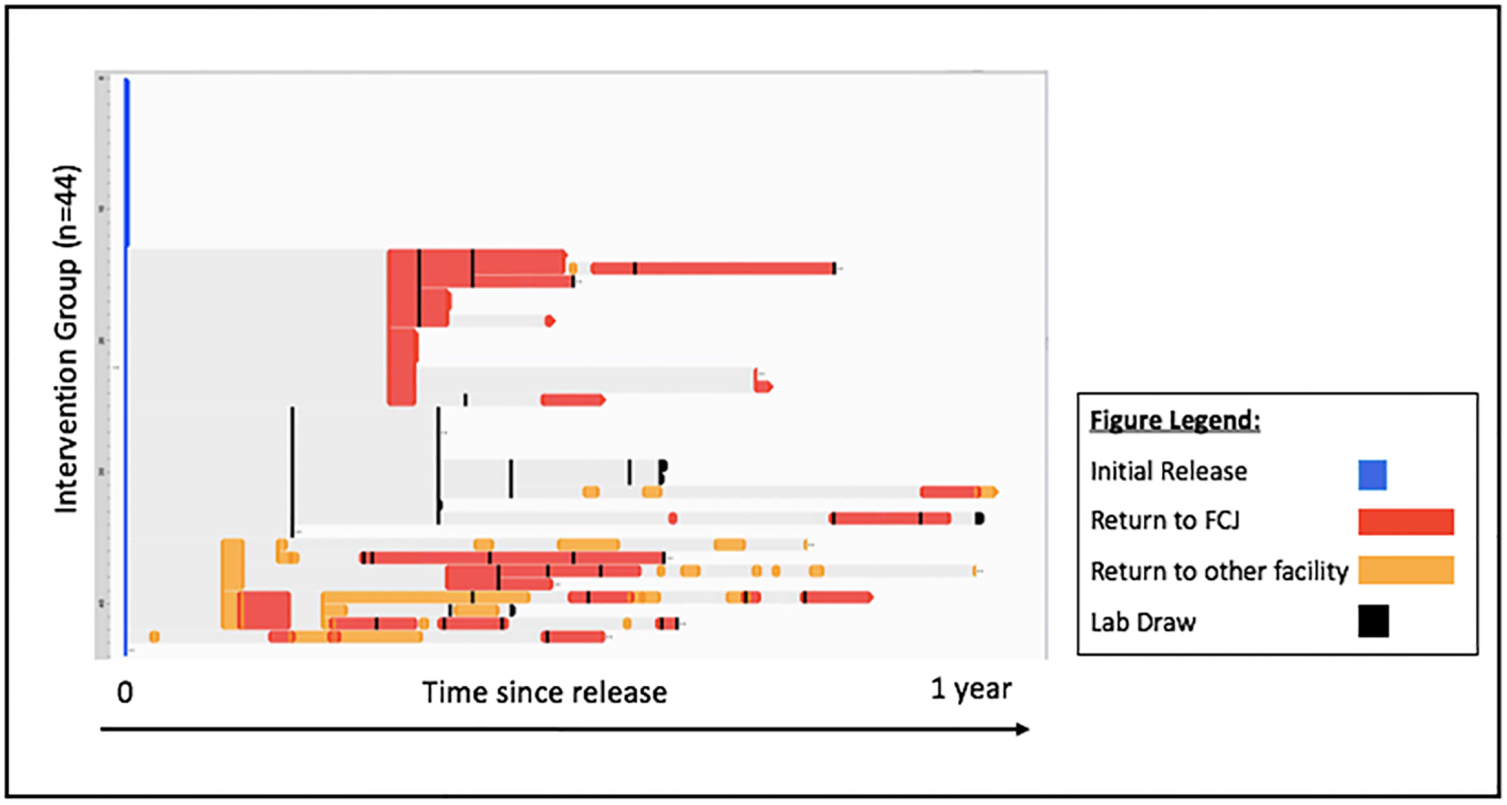
Trajectory of HIV care and follow-up survey events for SUCCESS participants after index release from Fulton County Jail (FCJ), Atlanta, GA, 2014–2016. • SUCCESS is Sustained, Unbroken, Connection to Care, Entry Services, and Suppression; strengths-based case management intervention implemented among individuals released from Fulton County Jail, compared with usual jail discharge services. • This figure demonstrates an overview of returns to jail and lab draws for participants. • Event Flow visualization aligns like events; in this case, releases, return visits, and labs are grouped together based on when they occurred. • Time zero for each intervention participant is their initial release, marked in blue. • Thereafter, like events are grouped together temporally over the course of one year of follow-up. • For interval events such as returns to jail, the length of the intervals represents the mean duration of the grouped events. • For point events such as laboratory draws, periods between the aggregated point events represent the mean length of time from any previous point event. • Our aim was to begin the intervention in jail and complete it after release to the community; however, in practice, the intervention may have been incomplete before a participant returned to jail after their initial release.

Fig 2 details the trajectories of the intervention and comparison groups over one year following initial release. Half of intervention participants and 62% of the comparison group returned to jail within one year after release. The average length of time before the first return to jail for recidivating participants in the intervention group was 108.8 days, and the average duration of their subsequent stays was 35.2 days. Members of the comparison group who returned to jail did so, on average, 135.2 days after their initial release, and stayed in jail an average of 56.3 days. The maximum number of returns to jail in the year following release was 8 and 5 in the intervention and comparison groups, respectively. Sixty-four percent of intervention group participants completed either the 3-month or 12-month follow-up surveys, and 32% completed both (Fig 3). The majority of follow-up surveys were conducted in the community (73%), and the remaining were conducted during a return to jail. Surveys were typically conducted at the same visit as lab draws.

Table 2 shows data on the primary outcomes obtained from eHARS. Following initial release, 86.3% of individuals in the intervention group demonstrated linkage to care, compared with 75.5% in the comparison group. Furthermore, 52.3% of individuals in the intervention group were considered retained in care, compared with 40% in the comparison group. Retention differed by baseline viral load; those who were virally suppressed at baseline were more likely to be retained in care upon follow-up. Suppression at last recorded viral load measure in the year of follow-up was demonstrated by 38.6% of intervention participants and 51.1% of the comparison group. Notably, the viral loads of the intervention group was less often suppressed at baseline than those of the comparison group. Approximately 22% of participants in each group demonstrated a change in viral status from unsuppressed to suppressed over the course of the study.

**Table 2:** Linkage and retention of SUCCESS^1^ study participants, HIV infected persons released from Fulton County Jail, Atlanta, GA, 2014–2016^2^.

Variable	Intervention Group (n = 44) No. (%)	Comparison Group (n = 45) No. (%)	Unadjusted Odds Ratio (95% Confidence Interval)
Linked^3^^,^ ^4^	38 (86.3)	34 (75.5)	2.0 (0.68, 6.14)
Retained^5^	23 (52.3)	18 (40.0)	1.6 (0.71, 3.81)
Last known viral load ≤ 1500 copies/mL	19 (43.2)	25 (55.6)	0.61 (0.26, 1.41)
Last known viral load ≤ 200 copies/mL	17 (38.6)	23 (51.1)	0.60 (0.26, 1.40)

^1^ Sustained, Unbroken, Connection to Care, Entry Services, and Suppression; strengths-based case management intervention implemented among individuals released from Fulton County Jail, compared with usual jail discharge services.

^2^ Results from Georgia Department of Public Health Electronic HIV/AIDS Reporting System; Database housing records of all HIV/AIDS laboratory draws in the state of Georgia: after submission of identities of 44 intervention group and 45 comparison group participants, balanced by age and birth gender.

^3^ Linkage defined as having any labs recorded in eHARS within the year following initial release from jail

^4^ Two persons on whom we obtained laboratory data from their jail stay were not recognized by the eHARS database and retrieved no results at all. In addition, 4 persons with identities recognized by eHARS did not have labs registered in eHARS subsequent to their release.

^5^ Retention defined as having at least 2 labs spaced 3 months apart recorded in eHARS within the year following initial release from jail

**Fig 4:**
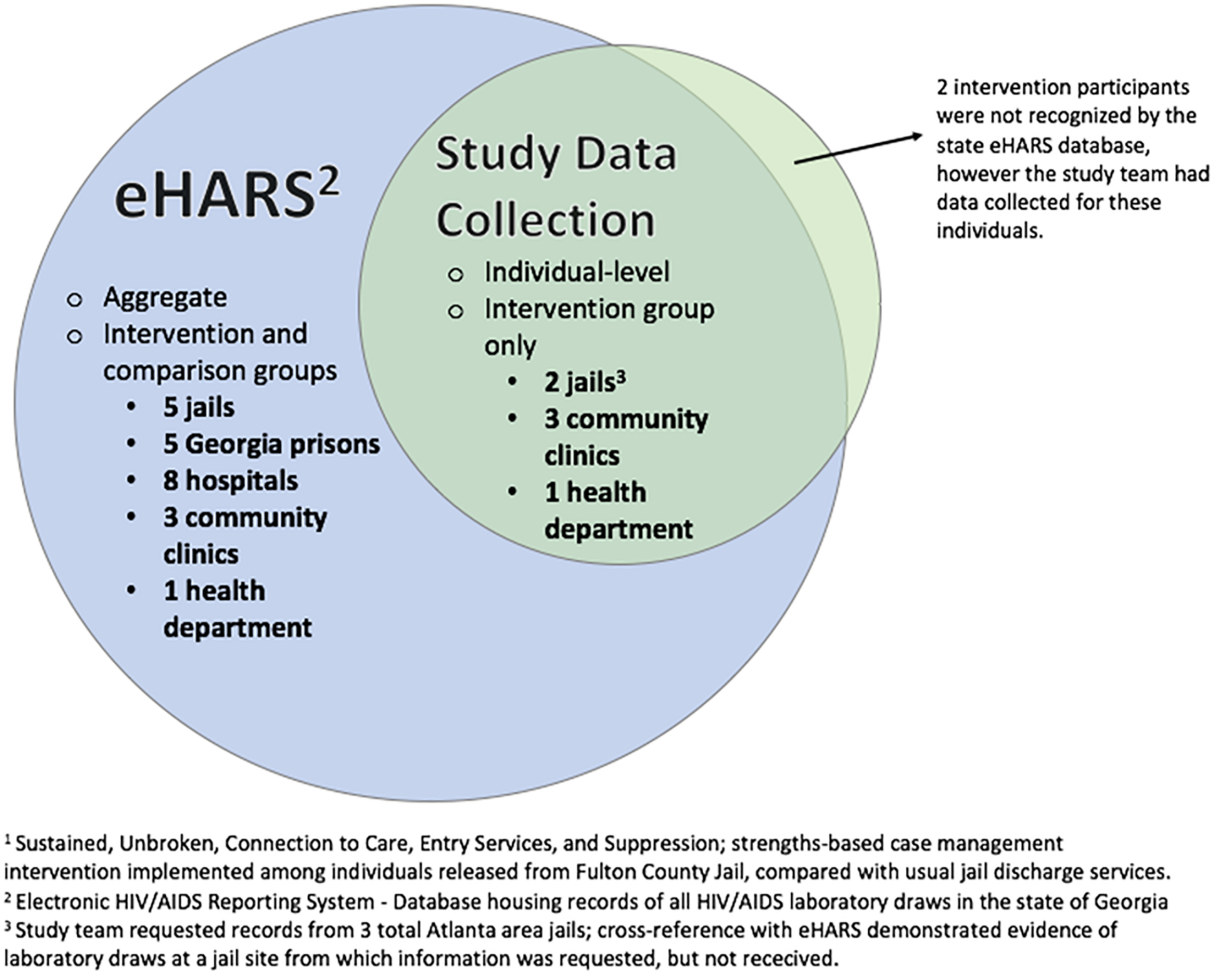
Schematic for SUCCESS study data collection on HIV infected persons released from Fulton County Jail, Atlanta, GA, 2014–2016. • SUCCESS is Sustained, Unbroken, Connection to Care, Entry Services, and Suppression; strengths-based case management intervention implemented among individuals released from Fulton County Jail, compared with usual jail discharge services. • This figure demonstrates the data collection structure and yield from HIV care sites identified by intervention group study participants at enrollment, as compared with the sites where care was truly received over the course of one year following release, according to state HIV care registry data.

As previously mentioned, the study team collected individual laboratory visit data for intervention group participants from locations at which participants had signed releases, including 2 jails in addition to the main site (FCJ), and 3 community clinics. In addition to the aforementioned sites, an in-depth assessment of eHARS results revealed laboratory draws completed in 4 more Atlanta area jails, 5 more Georgia prison facilities, and 12 more hospital or clinic locations among all study participants (Fig 4). Comparison of the individual outcomes data obtained by the study team with eHARS data indicated that eHARS captured an additional 13 individuals as linked to care, an additional 6 individuals as retained, and an additional 3 individuals as suppressed at last known viral load.

Table 3 demonstrates the results of a crude exploratory analysis of factors associated with retention (defined as either care in the community or upon a subsequent jail stay) and HIV viral suppression, as measured by individual-level laboratory data collection by the study team. As detailed in Table 3, we found that among intervention participants who were not homeless or unstably housed (OR = 0.13, [95% CI: 0.02, 0.78]), were more likely to have a suppressed viral load (p = 0.02). However, their retention in care was not substantially improved (OR = 0.41, [95% CI: 0.08–2.10]). Among those who were retained in care, having more than two returns to jail was associated with viral suppression (OR = 4.88, [95% CI: 1.10–21.69]). Among those participants who spent greater than 20% of the year after release back in jail, we found that they were more likely to be retained (OR = 5.60, [95% CI: 1.20, 26.14]) and suppressed (OR = 4.88, [95% CI: 1.10, 21.69]). Completing more of the SUCCESS curriculum (> = 4 sessions completed) was associated with better suppression (OR = 6.96, [95% CI: 1.33, 36.53]) and retention (OR = 11.38, [95% CI: 1.32, 98.31]); this may just represent a proxy for more time in jail. Similarly, texting was associated with greater suppression (OR = 5.53, 95% CI: 1.41, 21.66]), but it is unclear if the association was due to texting itself, or if ability to text represented an indicator of life stability.

**Table 3:** Unadjusted odds ratios for dichotomous variables with retention, suppressed viral load (≤200 copies/mL) for 44 SUCCESS^1^ intervention group participants at end of study^2^.

	Retention			Suppressed Viral Load (≤200 copies/mL)		
Sociodemographic	OR	95% CI	P-value^3^	OR	95% CI	P-value^3^
Age						
>37	4.08	1.11, 15.02	0.06	3.75	0.95, 14.76	0.10
≤ 37	referent					
Gender^4^						
Male	0.58	0.07, 4.61	0.63	1.38	0.13, 14.73	1.00
Female	referent					
Race						
Black, Black and Other	2.00	0.19, 20.97	1.0	No OR	Not estimable	0.30
Non-Black	referent					
Sexual Orientation^5^						
Not Heterosexual	1.20	0.29, 4.94	1.0	0.80	0.19, 3.35	1.00
Education						
High School graduate	1.37	0.34, 5.51	0.74	1.57	0.35, 7.02	0.72
Employment						
Employed	1.71	0.30, 9.68	0.66	No OR	Not estimable	0.15
Housing						
Homeless or Unstably Housed 30 days prior to jail stay^6^	0.41	0.08, 2.10	0.40	0.13	0.02, 0.78	**0.02**
**Health Characteristics**						
Baseline suppression status	1.23	0.24, 6.34	1.00	1.77	0.34, 9.27	0.66
Viral load <200 copies/mL						
Depression						
CES-D 10 Score ≥10	0.57	0.12, 2.65	0.70	0.38	0.08, 1.84	0.24
Alcohol Use						
AUDIT Score ≥8	1.56	0.42, 5.81	0.52	1.53	0.39, 5.95	0.72
Drug Use						
TCUDS Score ≥3	1.29	0.38, 4.39	0.76	3.60	0.95, 13.62	0.10
**Recidivism**						
Past year Jail Stays						
Never Jailed	1.97	0.57, 6.88	0.36	1.17	0.32, 4.19	1.0
Returns to jail						
Any return in the follow-up year	0.61	0.18, 2.08	0.72	2.86	0.73, 11.17	0.18
Number of Returns						
> 2 returns in the follow-up year	4.36	0.92, 20.74	0.07	6.75	1.37, 33.26	**0.02**
Length of Initial Stay						
≥ 2 months	3.11	0.83, 11.59	0.11	2.06	0.54, 7.82	0.32
Percentage of follow-up year in jail						
> 20%	5.60	1.20, 26.14	**0.03**	4.88	1.10, 21.69	**0.05**
**Study Engagement**						
Case Management						
≥ 4 sessions completed	6.96	1.33, 36.53	**0.02**	11.38	1.32, 98.31	**0.02**
Follow-up Engagement						
At least one follow-up survey completed	20.00	2.31, 73.11	**0.001**	5.25	1.00, 27.61	**0.05**
Texting						
Any two-way texting	5.53	1.41, 21.66	**0.01**	3.27	0.83, 12.81	0.11

^1^ Sustained, Unbroken, Connection to Care, Entry Services, and Suppression

^2^ Based only on subset of laboratory results retrieved by study team.

^3^ Significance assessed by Chi-square and Fisher’s exact tests.

^4^ Two male-to-female transgender individuals excluded.

^5^ 1 missing, refused to respond.

^6^ Unstably housed defined as not owning or renting a home 30 days prior to incarceration.

## Discussion

The findings reflect a trend towards increased linkage and retention in clinical care in the year following release from jail among PLWH receiving the SUCCESS strengths-based case management compared to PLWH in a comparison group jailed in a similar period of time. The typical recipients of the intervention, young to middle-aged HIV-infected Black men, constitute one of the groups with greatest challenges in linking to care. [22–24]

The ability to track outcomes using the Georgia Department of Public Health’s eHARS system was an invaluable tool to identify indicators of improved retention. Despite expending considerable effort to capture post-release laboratory results as a marker of engagement in care, our team gathered viral loads for only two-thirds (66%, or 25/38) of the intervention participants who had laboratory results post-release. The eHARS registry delivered data in aggregate and we do not yet have information on whether or not laboratory tests were drawn inside or outside of jail. Nonetheless, our results suggest care based in jail is an important component of follow-up HIV management of the cohort. Because outcomes data were available exclusively in the aggregate, only unadjusted odds ratios alone could be calculated for the main outcomes shown in Table 2. Of the laboratory draws we identified individually, half of the laboratories in the year after release were drawn upon return to jail.

Reincarceration is a known challenge to management of HIV infection among those ever incarcerated and was observed among approximately half of our study participants. Recidivism continues to be a barrier to sustaining exclusively community-based HIV care. Some prison HIV studies treat both lack of engagement in community care and recidivism as intervention failures.[6] Instead, we proposed acknowledging recidivism in our studies, and measured engagement for PLWH both inside and outside of jail. Advocating for decarceration is laudable. But to hold correctional health interventions to a standard that demands they must address both medical and criminal justice goals simultaneously may overlook successful strategies that improve HIV care. As long as interventions do not promote recidivism, success should be assessed by whether they facilitate engagement in care. When assessing outcomes of interventions to promote linkage to care among justice-involved persons, we have advocated disentangling failure to reach a medical goal from failure to attain a criminal justice objective.[25]

Previous studies of engagement in HIV care following release from a correctional setting have concluded that behavioral interventions failed to show an improvement over control conditions,[6] making the results of this study encouraging. The change in retention (52.3% with intervention, 40.0% with comparison group) was in the same direction and magnitude as was shown in the ARTAS study.[11] A jail-based intervention that increases engagement in care on the scale of ARTAS would be cost-effective.[26] In an observational study of jail detention, homelessness and HIV care among New York City residents published last year, a state-wide registry performed a similar function to eHARS.[8] Both the New York study and ours showed that jails, the “big house”, are the “medical home” for many poor urban residents with HIV, or at least an important “home away from home”.

A recent longitudinal analysis among HIV patients seeking care at a large, urban clinic in Atlanta demonstrated the need for granular, patient-centered data on engagement in HIV care.[27] While our feasibility study was not able to follow individuals beyond one year, we focused on presenting holistic, participant-level information regarding care received, whether at community locations, or in jail. Further analysis of the results will give insight into what factors may predict linkage and retention in care. Both the SUCCESS intervention and ARTAS borrowed from theories of empowerment and self-efficacy, and a sense of agency may be key to engagement in care.[11] Retention in care was associated with texting, but it is unclear if texting was a marker of more stability in the life of the participant or if it facilitated linkage.

### Limitations

Interpretation of these results should take into consideration several limitations of the study. First, we defined return to the correctional setting as an entry in the portion of the facility’s electronic reporting system that was available to the investigators. If a stay in an institution was expunged from the record, which sometimes happens in the criminal justice system, then our recidivism results may be biased. Second, not all laboratory data that we retrieved were in the eHARS system. While the eHARS data were helpful in providing an overall picture of linkage and retention, because the data were provided in the aggregate we were not able to locate a substantial portion of the individual laboratory results. This impeded our ability to track accurately which individuals had their virus suppressed during the course of the year after initial release. Furthermore, the calculated measures of association with our outcome variables were also limited based on the data we were able to collect. The retention and last known viral load status assigned to each participant in our study may or may not reflect the true status of the participants given that our data were not complete when compared with eHARS. Neither the eHARS system nor data retrieval by the investigative team can be considered a gold standard.

Third, assignment to the comparison condition was not random and the two groups, while close in age, were not balanced by starting viral load. This difference, and the fact the study was not designed to be adequately powered to show a significant change in linkage and retention, lessens our ability to speculate on whether the intervention had a causal association with the outcome. Because persons in the comparison group did not undergo an interview, we are unaware if the two groups differed in other respects, such as whether they owned a phone. Given that most persons in the intervention had a phone, likely those in the comparison group also owned phones. A recent analysis that included participants from this cohort showed that sending text messages to individuals recently released from jail likely has a negligible effect on linkage to care.[28]

Fourth, our feasibility study is too small to study formally the effect of a higher dose of the intervention. Participants in the intervention group completed an average of 4.3 case management sessions. A larger study would be able to determine if there were an incremental improvement in outcomes for each additional session completed. Furthermore, more complete information on the demographics of participants in both arms would permit adjusting the OR for confounders.

The OR for comparing the two arms on these outcomes crossed the null in this small feasibility study; we believe the lack of significance was due to inadequate power. Nonetheless, we now have data that can guide us for designing an adequately powered, future randomized trial. Given that the prevalence of retention in the exposed arm was 52% and in the unexposed was 40%, with an alpha of 0.05, we would have 80% power to detect a significant difference in arms in a future trial if our sample size is about 540. Our recruitment in this one jail was 14 subjects per month. Atlanta has five mega-jails about the size of Fulton County Jail.[29] Recruiting in two jails at the rate of 14 participants a month, the rate of accrual in this study, in would result in enrollment of 540 participants over 19 months. In this future, larger study, we could conduct more detailed event history analysis, similar to what Fig 2 shows, for both arms, and compare the sequence of events by arm.

## Conclusion

Receipt of SUCCESS ostensibly was associated with improvements in retention in care for PLWH released from jail, compared to usual services. A larger randomized, controlled trial is needed to determine if the difference is significant and cost-effective. Previous studies of linkage post-incarceration only examined care at community clinics. Since recidivism is common, measurement of linkage and retention should include care at all venues, including jail-based clinics.

## Supporting information

S1 File SUCCESS client session guide. (PDF)

S2 File TREND statement checklist. (PDF)

S3 File De-identified SUCCESS participant data. (XLSX)

S4 File IRB protocols and approvals (Compressed/Zip File Archive). (ZIP)
