# Supplementary material for: Jail, an unappreciated medical home: Assessing the feasibility of a strengths-based case management intervention to improve the care retention of HIV-infected persons once released from jail
Source: PLoS One. 2018 Mar 30;13(3):e0191643. doi: 10.1371/journal.pone.0191643 (PMC5877830; doi:10.1371/journal.pone.0191643)
Supplement: S1 File — (PDF) [file pone.0191643.s001.pdf]

SUCCESS

Sustained Unbroken Connections
to Care, Entry Services and Suppression

CLIENT SESSION GUIDE

The Client Session Guide contains a step-by-step guide to facilitate each of the six client sessions, helpful tips, and the forms and documents needed to conduct and track each client session. This guide is written for the Linkage Coordinator implementing SUCCESS.

The goal of SUCCESS is to link people with HIV who are recently incarcerated to medical care. To advocate for linkage to medical care with the client, you will help the client identify benefits and resolve barriers to linkage. Undoubtedly, the client will have other goals s/he would like to address; these goals should be achievable in a short time frame and should not conflict with the Core Elements of SUCCESS. Accomplishing the client's other goals may strengthen the relationship between you and the client and/or eliminate a source of stress for the client.

In addition, many clients identify system-level barriers, such as restrictive service application hours, biases against people with living with HIV/AIDS, and a lack of childcare. Addressing these barriers will help facilitate linkage to medical care. Therefore, you should be skilled in resolving common system-level barriers.

Instructions on How to Use the Client Session Guide

Note: As noted previously, SUCCESS is an individual-level intervention. As such, the content, timing, and structure of each client session will differ greatly depending on the client and his/her needs, barriers, and strengths. You should be prepared to adjust the session content, timing, and structure to the client. The following Client Session Guide provides a basic structure for each client session.

The six client sessions are:
Session One: Developing the Contact Plan
Session Two: Building Rapport
Session Three: Contacting the Medical Clinic

Session Four: Strengthening Linkages
Session Five: Reviewing Progress
Session Six: Completing the Work/Future Transitions

Format of this Guide: A cover page with the overall activities for the session, an agenda, forms and documents needed, and an estimated length of time is provided for each client session. While you should attempt to complete every activity listed for each session, since SUCCESS is client-centered and the sessions are client-driven, you must be flexible in addressing the client's needs. Therefore, that solutions to client's needs are strengths-focused and client-driven is more important than completing all session activities. As such, the agenda, time, content, and forms

must be adjusted to the client's needs. After the cover page, each session is organized by agenda items, which correspond to one or two activities for the session. For each agenda item, you will find the following subheadings:

Purpose: The purpose is the activity or activities you should accomplish by the end of this discussion.
Forms and Documents: These are the forms and documents that should be on hand to review and/or complete with the client. Some forms will be completed after the session ends, and they are listed within the step-by-step procedure. Please remember, you may not use every form or document with each client. It depends on where the client is in his/her decision to link to medical care.
Advanced Preparation: The advanced preparation is a list of activities you should do before starting a client session that typically relate to the key considerations.
Key Considerations: The key considerations are reminders for you. These items may include information on what to expect from the client (e.g., a client may be ambivalent about the first session).
Procedure: The procedure is a step-by-step description of how to conduct each of the session activities. Because this is an individual-level intervention, the structure of this section will differ greatly depending on the client. Within this section you will find guidelines on what to cover and what to skip based on where the client is at that point in the session.

Finally, the content within each of the six sessions is intentionally redundant in places. Because a client may be under a great deal of stress and/or at different stages of decision-making from one session to the next, repeating information and key points are important to ensure s/he understands and retains the information. As you progress from one session to the next with the client, you will also note subtle differences in the step-by-step procedures and key considerations. These differences are reminders for you to check in with the client on unresolved barriers and further explore why s/he is not ready to link to medical care. The differences are noted throughout the Client Session Guide.

As a supplement to individual client sessions, a section called Client Session Guide Forms includes instructions and the forms needed during and after each client session. Some forms will be completed with the client, and others will be completed after the client session. This information will be provided in the instructions and in the step-by-step procedure. Finally, the guide provides an overview of helpful tips to consider when implementing SUCCESS. See Figure 1: SUCCESS Client Session Flow Process on the next page for a visual representation of the client sessions. The figure depicts how a client flows into SUCCESS and through the client sessions, linkage to medical care, and referral to other programs upon completion of SUCCESS.

It also shows how SUCCESS complements long-term/Ryan White case management and CRCS. A client can be referred from long-term/Ryan White case management or CRCS into SUCCES to provide more intensive, individualized work to link him/her to medical care. Or, a client can also be referred to these programs if s/he is not interested in or eligible for SUCCESS, or upon completion of SUCCESS.

Figure 1. Patient Flow in SUCCESS

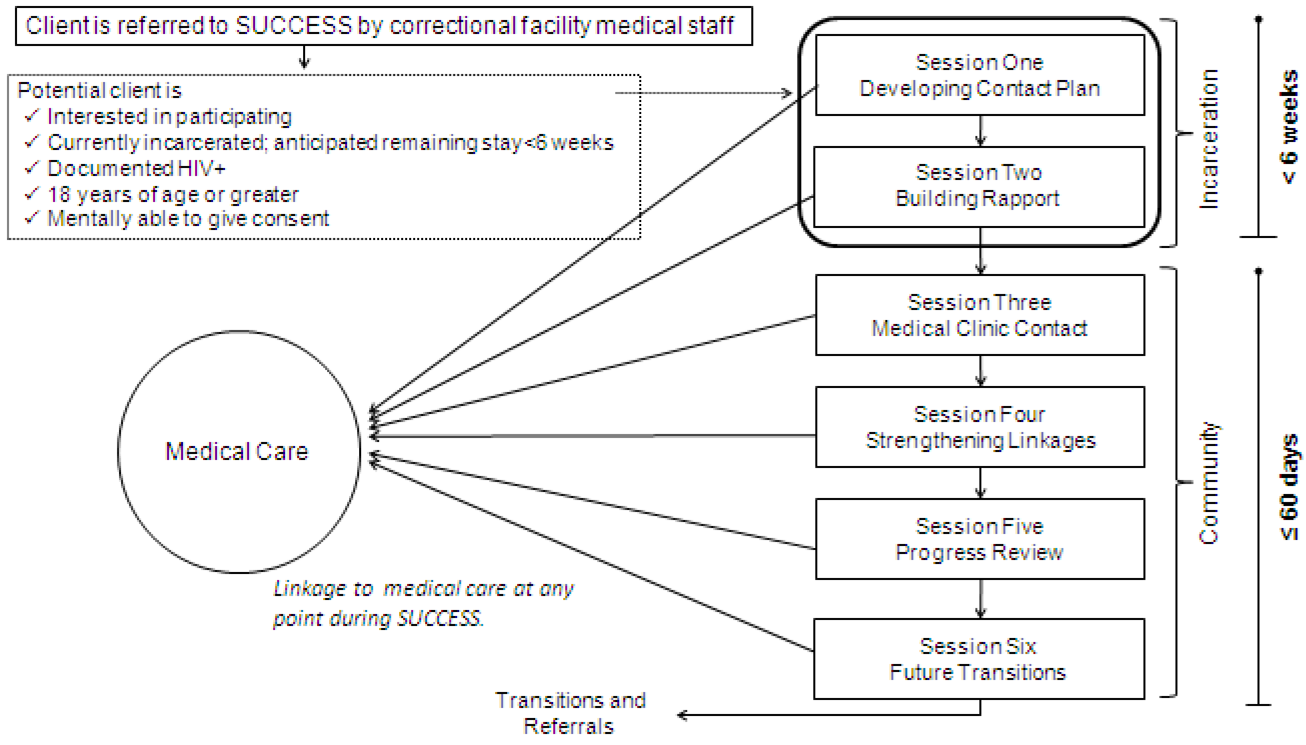

SESSION ONE: DEVELOPING THE CONTACT PLAN

Approximately: 1 ½-2 Hours

Session One Activities

A: Introduce goals of case management and SUCCESS
B: Discuss concerns about recent HIV diagnosis
C: Begin to identify personal strengths, abilities, and skills, and assess others' roles in impeding or promoting access to services
D: Encourage linkage to medical care
E: Summarize the session, the client's strengths and resources, and agreed-upon next steps
F: Plan for the next session(s), with the medical care provider and/or you

Session One Guide Agenda

1A. Introduction
1B. Guided Discussion
1C. Client Assessment

1D. Linkage to Medical Care
1E. Review and Summarize the Session
1F. Schedule Medical Appointment and/or Next Session

Forms and Documents Needed for Session One:

- Overview of SUCCESS document
- Strengths Assessment
- SUCCESS Session Plan
- Resource Directory
  - A Listing of Medical and Psychiatric Service Providers and Local Social Service Providers (e.g., housing, food, insurance)
- Fact Sheet on Current Treatment Options and their Side Effects
- Appointment Cards
- Incentives, if provided
- Session Notes
- Session Notes Summary Sheet
- Case Staffing Form
- Life Domains List

Remember: The sessions are client-driven. As such, <u>the agenda, time, content, and forms must be adjusted to the client's needs.</u>

1A: Introduction

Purpose: Introduce yourself and ensure the client understands the goals of case management, SUCCESS, and the strengths model used to guide the process.

Forms and Documents: - *Overview of SUCCESS Document*
- *Brief SUCCESS Summary*

(Note: During the Pre-Implementation phase, a brief SUCCESS summary should have been created to reach out to community partners. Give the client that document or a modified version).

Advanced Preparation:
- Review the Overview of SUCCESS Document.

Key Considerations: Remember that:
- A client may be ambivalent about the first session.
- Each client begins at a different place. Some may have just learned of their HIV status; others may have been living with HIV for some time.
- The client may have already overcome some barriers by attending this session.
- The client may have experienced a wide range of emotions leading up to this session, including fear, anger, distrust, helplessness, and fatalism.
- The client might have had negative personal experiences with medical providers in the past.
- The client may be dealing with issues related to incarceration and HIV linkage may not be his/her focus or priority.

Caution should be exercised to not self-disclose at this point. At this early stage, it is not possible to know what shared life experiences will enhance or impede your relationship with the client. This applies to issues such as personal faith, HIV status, relationships with others living with HIV or who have died from AIDS, or past substance use.

Procedure:
For all clients:

1. Introduce yourself to the client. Describe your professional background, especially as it applies to working with people living with HIV/AIDS (PLWHA). Emphasize your training, interest in assisting PLWHA, and/or knowledge of HIV/AIDS-specific health care services.

2. Give the client the Overview of SUCCESS document and the brief SUCCESS summary. Either read the overview verbatim or paraphrase its key points. It is important that you confidently convey the key points to the client. The key points for the **SUCCESS Overview Document** can be found on page 72 in the Session Forms section.

3. **Next step, continue to 1B: Guided Discussion.**

1B: Guided Discussion

Purpose: To give the client an opportunity to talk about his/her feelings and thoughts related to his/her recent HIV diagnosis.

Forms and Documents: - *Fact Sheet on Current Treatment Options and their Side Effects*

Advanced Preparation:
- Review the Fact Sheet on Current Treatment Options and their Side Effects.

Key Considerations: You should:
- Possess a comprehensive and in-depth knowledge base about HIV/AIDS (the medical, psychological, and social aspects), and be able to answer the client's detailed questions.
- Refer to current resources to answer the client's questions.
- Promote the personal and partner benefits of risk reduction and the value of seeking medical care early.
- Diminish fears or concerns the client might have about treatment and/or visiting a doctor.
- Be realistic about the limitations of treatment – that there is no cure for HIV; however, instill hope related to healthy outcomes in the client.
- Help the client explore personal resources to help him/her to be successful.
- Be careful to neither directly confront nor reinforce the client's statements at this time.

Procedure:
For all clients:
1. Start the discussion with a statement that lets the client know you understand and are aware that it is natural to have many feelings and unanswered questions after receiving an HIV-positive diagnosis. Start the discussion like this:
   • *"When a person finds out they're HIV-positive, a lot of things go through their mind. How have you been feeling since you found out?"*

   Possible open-ended follow-up questions include:
   - *"What resources did the health department tell you about when you received your test results?"* and
   - *"What were your biggest worries when you received your positive test results?"*

2. Ask the client what materials about HIV/AIDS the testing site gave him/her, if any. Possible open-ended follow-up questions include:
   - *"What did you think about the material you received?"* or
   - *"What additional questions do you have about HIV/AIDS?"*

3. Clarify any questions the client has about HIV (specifically about symptoms, care and treatment options, support services, and counseling).

4. Review the Fact Sheet on Current Treatment Options and Side Effects (see sample on page 73 of the Session Forms section), and offer to give him/her a copy of the document. Possible open-ended follow-up questions include:
    - "What other questions do you have?"
    - "Have you discussed your HIV status with a doctor or nurse since you received your test results? If so, what did you talk about? Do you have additional questions?" and
    - "What are your concerns about seeking treatment or medical care?"

5. Next step, **continue to 1C: Client Assessment.**

1C: Client Assessment

Purpose: Begin to identify personal strengths, abilities, and skills and assess others' role in impeding or promoting access to medical and/or social services.

Forms and Documents: - *Strengths Assessment*

Advanced Preparation:
- Review the state/local legal requirements regarding HIV disclosure.

Key Considerations: You should:
- Use effective communication skills.
- Know the state/local legal requirements regarding disclosing one's HIV status.
- Have natural conversations with the client to identify additional strengths.
- Ask open-ended questions that encourage the client to identify strengths.
- Show the client genuine respect and concern, as this is the starting point of a helping relationship.

Procedure:
For all clients:
1. Explain how identifying the client's strengths, abilities, and skills relates to his/her ability to stay healthy and link to medical care. For example,:

    "Often times, when you see ways that you've been successful in the past, it helps you to be successful again. Knowing how you've been successful helps you plan how to deal with barriers or problems you may have getting the medical care you need or achieving other goals."

2. Ask the client to talk about his/her personal experiences. Guide the client to speak from a strengths perspective and about his/her abilities, rather than putting himself/herself down.

While the Strengths Assessment is formally introduced in **Session Two**, it is important to start talking about strengths from the very beginning.

3. Cite examples of the client's strengths and abilities that have already become apparent in your conversation or during this session. This will help the client think about personal strengths, resources, and skills. Some common examples include:
   - The courage to get tested for HIV
   - The wisdom to come to Session One of SUCCESS
   - The ability or desire to live independently
   - Being punctual, if s/he arrived on time

4. Help the client assess the role others have in supporting or impeding his/her access to medical care. Ask the client:
   - *"Who do you think could support or help you get to the doctor? Think about friends, family, neighbors, significant others, anyone. These are people you feel can take you to appointments, let you borrow their car, provide financial assistance, watch your kids, give emotional support, and other things you might need."*
5. Discuss the advantages or disadvantages of telling a significant other or sexual partner(s) about testing positive.

   If the client is currently involved in a sexual relationship(s), ask him/her:
   - *"Does your significant other/sexual partner(s) know you're HIV-positive or not?"*

   If yes, follow up with:
   - *"How do you think you could get [insert name of significant other/partner] to help you get into medical care?"*

   If no, follow up with:
   - *"What do you think are some of the advantages to telling [insert name of significant other/partner]?"* and
   - *"What are some of the disadvantages?"*

Discuss any important advantages or disadvantages that the client did not mention, including any state laws or legal requirements to disclose one's HIV status to sexual partners (regardless of condom use or other protective measures taken) and/or to health care providers. It is important that you be familiar with these requirements and be able to clearly articulate them to the client.

6. **Next step, continue to 1D: Linkage to Medical Care.**

1D: Linkage to Medical Care

Purpose: Encourage the client to seek medical care, and, if interested, assist him/her in the process to make that linkage.

Forms and Documents: - *SUCCESS Session Plan*
- *Resource Directory*
- *Session Notes*
- *Session Notes Summary Sheet*
- *Case Staffing Form*

Advanced Preparation:
- Review any specific requirements, characteristics/traits, agency policies, and required paperwork of the health care providers.
- Review any local, state, or federal policies such as eligibility requirements for services, Medicaid, ADAP, Ryan White services, wait lists, mandatory disclosure laws.

Key Considerations: Inform the client about the following:
- Care and treatment services provided at your agency and/or your community partners.
- Specific requirements, such as timeliness, rescheduling policies, or paperwork required for health care providers in the area.
- Characteristics or traits of a particular clinic(s) or community partner(s) that match the client's needs. For example, a clinic with bilingual staff or interpreters for a non-English-speaking/limited-English-proficient client.
- Relevant policies (at the agency, local, state, and/or federal levels), issues, or potential barriers, such as eligibility requirements for services, Medicaid, ADAP, Ryan White services, wait lists, mandatory disclosure laws. Focus on policies with the most immediate impact on the client.

Procedure:
For all clients:
1. Ask the client about his/her expectations and concerns about seeking medical care and treatment for HIV. Be sensitive to the client's stated and unstated reasons for not wanting to seek medical treatment. Begin the discussion with these questions:
   - *"What are your thoughts about linking to medical care?"*
   - *"What barriers or problems might get in the way of your going to a doctor or medical clinic?"*

2. Assess the client's tangible and perceived barriers. The client may have a multitude of personal barriers that impede his/her ability to seek services, such as:
   - Homelessness
   - No transportation
   - No financial resources
   - Active drug or alcohol addiction

Some of the perceived barriers could be fears about family, friends, and community members discovering his/her HIV status or health care needs.

3. Engage the client in a discussion about medical options, provide information, and help him/her clarify concerns, issues, and barriers. Remember, it is not your role to make the decision to link to medical care for the client.

4. Demonstrate your thorough knowledge of the medical care environment and requirements and provide information based on available resources. This includes:
    - Providers and their specialties and personalities
    - How to navigate the system to apply for and access Ryan White, Medicaid, or other services
    - In other words, all the background research you did in the Pre-Implementation Section to become familiar with community partners

---

At this point, one of four things is likely to happen. Based on where the client is in his/her decision, follow these instructions:

If the client decides to link to medical care at this point, continue with Step 5.

If the client is not ready to make this decision, skip to 1E: Review and Summarize the Session.

If the client a) wants to drop out of SUCCESS, or b) does not want to link to medical care, skip to Step 8.

For clients who wish to link to medical care at this point:

5. Introduce the SUCCESS Session Plan:

    "Our goal is to help you get connected to a doctor. As you may recall, we will have up to six sessions in 90 days to help you achieve this and other goals by identifying your strengths and overcoming barriers.

    The SUCCESS Session Plan is one of the activities that can guide us to accomplish your goal(s). This plan will help us organize our work together and make sure that we identify everything we need to work on. We'll write down the goals to remind us of what we're doing and you will always have a copy of your most recent SUCCESS Session Plan, if you want it."

6. Follow the **SUCCESS Session Plan Instructions** on page 70 of the Session Forms section. The SUCCESS Session Plan helps the client identify objectives and possible barriers, activities to accomplish the objective(s), the person responsible, target dates to complete each activity, and the related strength. It is recommended that the plan be committed to in writing to allow you and the client to easily track progress and pinpoint activities that may need to be adjusted over time.

7. **Next step, continue to 1E: Review and Summarize the Session.**

For clients who want to drop out of SUCCESS <u>or</u> do not want to link to medical care now or in the near future (<u>if at any point the client decides to link to medical care and/or not drop out of SUCCESS, continue to 1E: Review and Summarize the Session</u>):

8. Keep the conversation positive! Cover the following topics:
   a. Engage the client in a discussion about: (1) his/her reasons for attending the first SUCCESS session; and (2) his/her reasons for deciding not to continue with SUCCESS/seeking medical care.
   b. Let the client know that ambiguity about linking to medical care is normal.
   c. Review the client's strengths discussed during the session.
   d. Discuss his/her accomplishments made during the session and ask how, if at all, the session has been helpful.
   e. Keep the door open. Remind the client that your sessions together can continue as long as s/he thinks it can help clarify and remove barriers to seeking treatment before the end of the 90 days.
   f. Offer the client your business card and end the session.

9. **Next step, end the session and complete paperwork**:
   - Depending on your agency's procedures and/or schedule for the day, you may want to take a few minutes to complete all the required paperwork before moving on to the next client and/or task. Recommended paperwork to complete includes:
     - Session Notes
     - Session Notes Summary Sheet (if the client dropped out)
     - Case Staffing Form

1E: Review and Summarize the Session

Purpose: To review what was discussed with the client during the session and summarize the agreed-upon next steps.

Forms and Documents: - *SUCCESS Session Plan*

Advanced Preparation: None.

Key Considerations: You should:
- Review the SUCCESS Session Plan activities and discuss/revise anything that was documented incorrectly, if a plan was developed.

Procedure: For clients who wish to link to medical care at this point, or clients who are not ready to make this decision:

1. Provide a summary of the session or ask the client to summarize the session and the client's strengths. During the first session, the client may be very emotional and upset, particularly if s/he has been recently diagnosed. Therefore, summarizing the session is extremely important to help the client remember the key points.

2. Review the SUCCESS Session Plan activities, person responsible, and target date to complete the items with the client, if a plan was developed. Remember, some of these are activities you are committing to complete prior to the session.

3. **Next step, continue to 1F: Schedule Medical Appointment and/or Next Session.**

1F: Schedule Medical Appointment and/or Next Session

Purpose: To schedule appointments with you, medical providers, and other support services as needed.

Forms and Documents: - *SUCCESS Session Plan*
- *Resource Directory*
- *Appointment Cards*
- *Incentive, if provided*
- *Session Notes*
- *Session Notes Summary Sheet*
- *Case Staffing Form*

Advanced Preparation:

- Review your availability for the next session and/or medical appointment.
- Review the Resource Directory for medical providers, clinics, and other services as needed.
- Bring the transportation vouchers/tokens/schedules.

Key Considerations: You should:

- Refer the client to services as needed. The client may present with other needs that are related to his/her recent seroconversion or existing HIV status.
- If the client wishes to schedule a medical appointment, provide him/her with detailed information about the clinic hours and services they provide.
- Make sure all paperwork is completed and discuss how client information will be used. Stress privacy and confidentiality.
- Arrange and confirm all appointments with or for the client, medical as well as other services as needed.
- Offer to take the client or provide transportation to all scheduled appointments.
- Work on all identified barriers to following through with scheduled appointments.

Procedure:

For clients who wish to link to medical care at this point:

1. Clarify whether or not the client would like you to accompany him/her to the medical appointment.

2. Discuss the best time and date to schedule the appointment.
   - If the SUCCESS Session Plan has activities that must be completed before the medical appointment that may take some time, schedule the appointment further out or wait until the next session to schedule the medical appointment. Examples of activities that might take more time to complete are arranging for transportation and processing Medicaid enrollment forms.

3. Call the clinic or community partner (or have the client call) to schedule an appointment.
   - If the next time you see the client will be at the medical visit:
     - Give him/her information about the staff and doctor, required documents.
     - Discuss in detail what the client should expect at each stage of the appointment.
     - Help the client write down questions s/he would like to ask the health care provider and/or other clinic staff. Depending on the client, practice asking and answering questions with him/her so s/he feels comfortable with the list of prepared questions.
     - Ask the client if s/he would like for you to call him/her before the medical appointment, as a reminder.

For all clients:

4. Schedule and/or make arrangements for the client to access needed social services, such as temporary housing and food banks.

5. Schedule a day, time, and meeting location for the next SUCCESS session. Make sure accommodations are compatible with agency safety guidelines.
   - If the next session is <u>before the medical appointment or the client is not linking to medical care at this point</u>, offer to write down these details and to call the client before your next session as a reminder. The topics covered will follow the format for **Session Two**.
   - If it is scheduled for <u>after the medical appointment</u>, the next session will be **Session Six** (completing the work with the client).

6. Offer the client an **appointment card** (see samples on page 79 of the Session Forms section) to document the time, location, and agency name.

7. Give the client transportation tokens or vouchers to get home and/or to the next session or appointment. Or offer to pick the client up, if that is an allowable activity at your agency.

8. Gather any contact and/or locator information from the client before s/he leaves. Locator information will allow you to locate the client through family, friends, or other individuals that know how to reach him/her if the client's address changes, phone is disconnected, or the client is not reachable through the means provided in the initial intake (conducted within the agreed-upon rules for communicating with the client). Remind the client that you will attempt to contact him/her through these means only after a missed appointment.

    - To gather this information, discuss how the locator information will be used and be sure to inform the client that none of his/her personal information will be shared with the contacts provided. Information collected from the contact persons includes the following: usual place of residence; telephone number or address of someone who usually knows where the client can be found; places where s/he picks up mail or messages.
    - Ask the client if the contacts are aware of his/her HIV status and assure the client that his/her contacts will not be told the reason for the call. You can say you are a friend trying to reach the client.

9. End the session by thanking the client for coming and congratulate him/her for a productive session. Remind the client that linking to medical care is important to his/her overall health, and that you are there to help him/her attain services needed so that s/he is ready to access medical care and treatment.

10. **Next step, complete paperwork**:
    - Depending on your agency's procedures and/or schedule for the day, you may want to take a few minutes to complete all the required paperwork before moving on to the next client and/or task. Recommended paperwork to complete includes:
        - Session Notes
        - Session Notes Summary Sheet (if the client dropped out)
        - Case Staffing Form

©2017 Center for Health of Incarcerated Persons, Rollins SPH, Emory University

SESSION TWO: BUILDING RAPPORT

Approximately: 1 ½ - 2 hours

Session Two Activities

A: Solicit client issues and questions from the initial session
B: Continue identifying personal strengths, abilities, and skills
C: Encourage linkage to medical care
D: Identify and address personal needs and barriers to linkage
E: Summarize the session, the client's strengths, and agreed-upon next steps
F: Plan for the next session(s), with the medical care provider and/or you

Session Two Guide Agenda

2A. Review of Session One
2B. Client Assessment
2C-D. Linkage to Medical Care
2E. Review and Summarize the Session
2F. Schedule Medical Appointment and/or Next Session

Forms and Documents Needed for Session Two:

- *SUCCESS Session Plan*
- *Strengths Assessment*
- *Resource Directory*

  - *A Listing of Medical and Psychiatric Service Providers and Local Social Service Providers (e.g., housing, food, insurance)*
- *Appointment Cards*
- *Incentive, if provided*
- *Session Notes*
- *Session Notes Summary Sheet*
- *Case Staffing Form*
- *Life Domains List*

Remember: The sessions are client-driven. As such, <u>the agenda, time, content, and forms must be adjusted to the client's needs.</u>

2A: Review of Session One

Purpose: To clarify and address any questions or areas of confusion the client has from the initial contact.

Forms and Documents: - *SUCCESS Session Plan*

Advanced Preparation:
- Review the client's SUCCESS Session Plan, if one was developed in the previous session.

Key Considerations: Remember that the client:
- Needs support and resources to effectively link to medical care. Be sure to review the client's needs and refer him/her to needed services to assist in accessing medical care.
- Often needs assistance to identify personal strengths and abilities to facilitate his/her linkage to medical care.
- May need to reflect on his/her HIV status and barriers encountered in disclosing his/her status to others and in accessing social services.

Procedure: **For all clients:**
1. Welcome the client back for Session Two and congratulate him/her on following up successfully with today's session. Recognize the many demands the client has and state how much you appreciate him/her taking time to meet with you.

2. Ask the client what questions, concerns, or new insights s/he has as a result of the first session. You may also want to ask about his/her thoughts about linking to medical care since your last session and any reactions s/he has to the focus on strengths, which will help you to assess whether or not s/he is starting to adopt the new approach.

3. Summarize any additional points made during the discussion.

For clients who have not decided to link to medical care:

4. **Next step, continue to 2B: Client Assessment.**

For clients who have decided to link to medical care but have not yet attended their appointment:

5. Review the outcomes of all activities listed on the SUCCESS Session Plan for both you and the client. If necessary, revise the plan.

6. Ask the client about any new barriers and/or strengths discovered as a result of completing the SUCCESS Session Plan activities. If necessary, revise the plan.

7. **Next step, skip to section 2E: Review and Summarize the Session.**

2B: Client Assessment

Purpose: To help the client self-identify personal strengths, abilities, and skills.

Forms and Documents: - *Resource Directory*
- *Strengths Assessment*

Advanced Preparation:
- Review the client's Strengths Assessment, if one was started in the previous session.

Key Considerations:
You should:
- Use effective communication skills.
- Know the state/local legal requirements regarding disclosing one's HIV status.
- Have natural conversations with the client to identify additional strengths.
- Ask open-ended questions that encourage the client to provide more substantive information to build on the list of strengths developed in Session One.
- Show the client genuine respect and concern, as this is the starting point of a helping relationship.

Procedure:
For clients who have not yet decided to link to medical care:
1. Remind the client how identifying strengths, abilities, and skills relate to his/her ability to stay healthy and link to medical care. For example:
   "Often times, when you see ways that you've been successful in the past, it helps you to be successful again. Knowing how you've been successful helps you plan how to deal with barriers or problems you may have getting the medical care you need or achieving other goals."

2. Remind the client of examples of his/her strengths and abilities that have already become apparent in the previous session. This will help the client think about personal strengths, resources, and skills. Some common examples include:
   - The courage to get tested for HIV
   - The wisdom to come to Sessions One and Two of SUCCESS
   - The ability or desire to live independently
   - Being punctual, if s/he arrived on time

3. **This is a new activity started in Session Two.** Conduct the **Strengths Assessment** by following the instructions and introduction script starting on page 64 of the Session Forms section.

4. **Next step, continue to 2C-D: Linkage to Medical Care.**

2C-D: Linkage to Medical Care

Purpose: Encourage the client to seek medical care, and, if interested, assist him/her in the process to make that linkage.

Forms and Documents: - *SUCCESS Session Plan*
- *Resource Directory*
- *Session Notes*
- *Session Notes Summary Sheet*
- *Case Staffing Form*

Advanced Preparation:
- Review any specific requirements, characteristics/traits, agency policies, and required paperwork of the health care providers.
- Review any local, state, or federal policies such as eligibility requirements for services, Medicaid, ADAP, Ryan White services, wait lists, mandatory disclosure laws.

Key Considerations: Inform the client about the following:
- Care and treatment services provided at your agency and/or your community partners.
- Specific requirements, such as timeliness, rescheduling policies, or paperwork required for health care providers in the area.
- Characteristics or traits of a particular clinic(s) or community partner(s) that match the client's needs. For example, a clinic with bilingual staff or interpreters for a non-English-speaking/limited-English-proficient client.
- Relevant policies (at the agency, local, state, and/or federal levels), issues, or potential barriers, such as eligibility requirements for services, Medicaid, ADAP, Ryan White services, wait lists, mandatory disclosure laws. Focus on policies with the most immediate impact on the client.

Procedure:
For all clients:

10. Ask the client about his/her expectations and concerns about seeking medical care and treatment for HIV. Be sensitive to the client's <u>stated and unstated reasons</u> for not wanting to seek medical treatment. Begin the discussion with these questions:
    - *"What are your thoughts about linking to medical care?"*
    - *"What barriers or problems might get in the way of your going to a doctor or medical clinic?"*

11. Assess the client's tangible and perceived barriers. The client may have a multitude of personal barriers that impede his/her ability to seek services, such as:

- Homelessness
- No transportation
- No financial resources
- Active drug or alcohol addiction

Some of the perceived barriers could be fears about family, friends, and community members discovering his/her HIV status or health care needs.

12. Engage the client in a discussion about medical options, provide information, and help him/her clarify concerns, issues, and barriers. Remember, it is not your role to make the decision to link to medical care for the client.

13. Demonstrate your thorough knowledge of the medical care environment and requirements and provide information based on available resources. This includes:
    - Providers and their specialties and personalities
    - How to navigate the system to apply for and access Ryan White, Medicaid, or other services
    - In other words, all the background research you did in the Pre-Implementation Section to become familiar with community partners

---

At this point, one of four things is likely to happen. Based on where the client is in his/her decision, follow these instructions:

If the client decides to link to medical care at this point, continue with Step 5.

If the client is not ready to make this decision, skip to 1E: Review and Summarize the Session.

If the client a) wants to drop out of SUCCESS, or b) does not want to link to medical care, skip to Step 8.

For clients who wish to link to medical care at this point:

14. Introduce the SUCCESS Session Plan:

    "Our goal is to help you get connected to a doctor. As you may recall, we will have up to six sessions in 90 days to help you achieve this and other goals by identifying your strengths and overcoming barriers.

    The SUCCESS Session Plan is one of the activities that can guide us to accomplish your goal(s). This plan will help us organize our work together and make sure that we identify everything we need to work on. We'll write down the goals to remind us of what we're doing and you will always have a copy of your most recent SUCCESS Session Plan, if you want it."

15. Follow the **SUCCESS Session Plan Instructions** on page 70 of the Session Forms section. The SUCCESS Session Plan helps the client identify objectives and possible barriers,

activities to accomplish the objective(s), the person responsible, target dates to complete each activity, and the related strength. It is recommended that the plan be committed to in writing to allow you and the client to easily track progress and pinpoint activities that may need to be adjusted over time.

16. **Next step, continue to 2E: Review and Summarize the Session.**

2E: Review and Summarize the Session

Purpose: To review what was discussed with the client during the session and summarize the agreed-upon next steps.

Forms and Documents: - *SUCCESS Session Plan*

Advanced Preparation: None.

Key Considerations: You should:
- Review the SUCCESS Session Plan activities and discuss/revise anything that was documented incorrectly, if a plan was developed.

Procedure:
For clients who wish to link to medical care at this point, or clients who are not ready to make this decision:

1. Provide a summary of the session or ask the client to summarize the session and the client's strengths. During the session, the client may be very emotional and upset, particularly if s/he has been recently diagnosed. Therefore, summarizing the session and the client's strengths are extremely important to help the client remember the key points.

2. Review the SUCCESS Session Plan activities, person responsible, and target date to complete the items with the client, if a plan was developed. Remember, some of these are activities you are committing to complete prior to the session.

3. **Next step, continue to 2F: Schedule Medical Appointment and/or Next Session.**

2F: Schedule Medical Appointment and/or Next Session

Purpose: To schedule appointments with you, medical providers, and support services as needed.

Forms and Documents: - *SUCCESS Session Plan*
- *Resource Directory*
- *Appointment Cards*
- *Incentive, if provided*
- *Session Notes* - *Session Notes Summary Sheet*

- *Case Staffing Form*

Advanced Preparation:

- Review your availability for the next session and/or medical appointment.
- Review the Resource Directory for medical providers, clinics, and other services as needed.
- Bring the transportation vouchers/tokens/schedules.

Key Considerations: You should:

- Refer the client to services as needed. The client may present with other needs that are related to his/her recent seroconversion or existing HIV status.
- If the client wishes to schedule a medical appointment, provide him/her with detailed information about the clinic hours and services they provide.
- Make sure all paperwork is completed and discuss how client information will be used. Stress privacy and confidentiality.
- Arrange and confirm all appointments with or for the client, medical as well as other services as needed.
- Offer to take the client or provide transportation to all scheduled appointments.
- Work on all identified barriers to following through with scheduled appointments.

Procedure:

For clients who wish to link to medical care at this point:

1. Clarify whether or not the client would like you to accompany him/her to the medical appointment.

2. Discuss the best time and date to schedule the appointment.
   - If the SUCCESS Session Plan has activities that must be completed before the medical appointment that may take some time, schedule the appointment further out or wait until the next session to schedule the medical appointment. Examples of activities that might take more time to complete are arranging for transportation and processing Medicaid enrollment forms.

3. Call the clinic or community partner (or have the client call) to schedule an appointment.
   - **If the next time you see the client will be at the medical visit**:
     - Give him/her information about the staff and doctor, required documents.
     - Discuss in detail what the client should expect at each stage of the appointment.
     - Help the client write down questions s/he would like to ask the health care provider and/or other clinic staff. Depending on the client, practice asking and answering questions with him/her so s/he feels comfortable with the list of prepared questions.
     - Ask the client if s/he would like for you to call him/her before the medical appointment, as a reminder.

For all clients:

4. Schedule and/or make arrangements for the client to access needed social services, such as temporary housing and food banks.

5. Schedule a day, time, and meeting location for the next SUCCESS session. Make sure accommodations are compatible with agency safety guidelines.

   - If the next session is before the medical appointment or the client is not linking to medical care at this point, offer to write down these details and to call the client before your next session as a reminder. The topics covered will follow the format for **Session Three**.
   - If it is scheduled for after the medical appointment, the next session will be **Session Six** (completing the work with the client).

6. Offer the client an **appointment card** (see samples on page 79 of the Session Forms section) to document the time, location, and agency name.

7. Give the client transportation tokens or vouchers to get home and/or to the next session or appointment. Or offer to pick the client up, if that is an allowable activity at your agency.

8. Gather any contact and/or locator information from the client before s/he leaves. Locator information will allow you to locate the client through family, friends, or other individuals who know how to reach him/her if the client's address changes, phone is disconnected, or the client is not reachable through the means provided in the initial intake (conducted within the agreed-upon rules for communicating with the client). Remind the client that you will attempt to contact him/her through these means only after a missed appointment.
   - To gather this information, discuss how the locator information will be used and be sure to inform the client that none of his/her personal information will be shared with the contacts provided. Information collected from the contact persons includes the following: usual place of residence; telephone number or address of someone who usually knows where the client can be found; places where s/he picks up mail or messages.
   - Ask the client if the contacts are aware of his/her HIV status and assure the client that his/her contacts will not be told the reason for the call. You can say you are a friend trying to reach the client.

9. End the session by thanking the client for coming and congratulate him/her for a productive session. Remind the client that linking to medical care is important to his/her overall health, and that you are there to help him/her attain services needed so that s/he is ready to access medical care and treatment.

10. **Next step, complete paperwork**:
    - Depending on your agency's procedures and/or schedule for the day, you may want to take a few minutes to complete all the required paperwork before moving on to the next client and/or task. Recommended paperwork to complete includes:

- ○ Session Notes
- ○ Session Notes Summary Sheet (if the client dropped out)
- ○ Case Staffing Form

SESSION THREE: MEDICAL CLINIC CONTACT

Approximately: 1 ½ - 2 hours

Session Three Activities

A: Solicit client issues and questions from Session Two
B: Continue identifying personal strengths, abilities, and skills
C: Encourage linkage to medical care
D: Identify and address personal needs and barriers to linkage
E: Summarize the session, the client's strengths, and agreed-upon next steps
F: Plan for the next session(s), with the medical care provider and/or you

Session Three Guide Agenda

3A. Review of Session Two
3B. Client Assessment
3C-D. Linkage to Medical Care
3E: Using SMS Technology to Enhance Linkages to Community Care for HIV+ Clients (adapted from 3C-D)
3F. Review and Summarize the Session
3G. Schedule Medical Appointment and/or Next Session

Forms and Documents Needed for Session Three:

- *SUCCESS Session Plan*
- *Strengths Assessment*
- *Resource Directory: A Listing of Medical and Psychiatric Service Providers and Local Social Service Providers (e.g., housing, food, insurance)*
- *Appointment Cards*
- *Incentive, if provided*
- *Session Notes*

- *Session Notes Summary Sheet*
- *Case Staffing Form*
- *Life Domains List*

Note: Many of the activities listed below are similar to the activities in previous sessions. However, there are a few slight variations to the step-by-step procedures listed. The variations are a result of this being the third session conducted, and you may need to expand on specific points to clarify why the client is still facing the same barriers, not deciding to link to medical care, or to continue to identify strengths.

Remember: The sessions are client-driven. As such, <u>the agenda, time, content, and forms must be adjusted to the client's needs.</u>

3A: Review of Session Two

Purpose: To clarify and address any questions or areas of confusion the client has from Session Two.

Forms and Documents: - *SUCCESS Session Plan*

Advanced Preparation:

- Review the client's SUCCESS Session plan, if one was developed in the previous session.

Key Considerations: Remember that the client:

- Needs support and resources to effectively link to medical care. Be sure to review the client's needs and refer him/her to needed services to assist in accessing medical care.
- Often needs assistance to identify personal strengths and abilities to facilitate his/her linkage to medical care.
- May need to reflect on his/her HIV status and barriers encountered in disclosing his/her status to others and in accessing social services.

Procedure: **For all clients:**

1. Welcome the client back for Session Three and congratulate him/her on following up successfully with today's session. Recognize the many demands the client has and state how much you appreciate him/her taking time to meet with you.

2. Ask the client what questions, concerns, or new insights s/he has as a result of the first two sessions. You may also want to ask about his/her thoughts about linking to medical care since

your last session and any reactions s/he has to the focus on strengths, which will help you to assess whether or not s/he is starting to adopt the new approach.

3. Summarize any additional points made during the discussion.

For clients who have not decided to link to medical care:

4. **Next step, continue to 3B: Client Assessment.**

For clients who have decided to link to medical care but have not yet attended their appointment:

5. Review the outcomes of all activities listed on the SUCCESS Session Plan for both you and the client. If necessary, revise the plan.
6. Ask the client about any new barriers and/or strengths discovered as a result of completing the SUCCESS Session Plan activities. If necessary, revise the plan.
7. **Next step, skip to section 3F: Review and Summarize the Session.**

3B: Client Assessment

Purpose: To continue to help the client self-identify personal strengths, abilities, and skills.

Forms and Documents: - *Resource Directory*
- *Strengths Assessment*

Advanced Preparation:

- Review the client's Strengths Assessment, if one was started in the previous session.

Key Considerations: You should:

- Use effective communication skills.
- Have natural conversations with the client to identify additional strengths.
- Ask open-ended questions that encourage the client to provide more substantive information to build on the list of strengths developed in Session One.
- Show the client genuine respect and concern, as this is the starting point of a helping relationship.

Procedure:
For clients who have not yet decided to link to medical care:

5. Remind the client how identifying strengths, abilities, and skills relate to his/her ability to stay healthy and link to medical care. For example:

"Often times, when you see ways that you've been successful in the past, it helps you to be successful again. Knowing how you've been successful helps you plan how to deal with barriers or problems you may have getting the medical care you need or achieving other goals."

6. Remind the client of examples of his/her strengths and abilities that have already become apparent in the previous session. This will help the client think about personal strengths, resources, and skills. Some common examples include:
   - The courage to get tested for HIV
   - The wisdom to come to the first three sessions of SUCCESS
   - The ability or desire to live independently
   - Being punctual, if s/he arrived on time

7. Continue the **Strengths Assessment** from Session 2 by following the instructions and introduction script starting on page 64 of the Session Forms section.

8. **Next step, continue to 3C-D: Linkage to Medical Care.**

3C-D: Linkage to Medical Care

Purpose: Encourage the client to seek medical care, and if interested, assist him/her in the process to make that linkage.

Forms and Documents: - *SUCCESS Session Plan*
- *Resource Directory*
- *Session Notes*
- *Session Notes Summary Sheet*
- *Case Staffing Form*

Advanced Preparation:

- Review any specific requirements, characteristics/traits, agency policies, and required paperwork of the health care providers.
- Review any local, state, or federal policies such as eligibility requirements for services, Medicaid, ADAP, Ryan White services, wait lists, mandatory disclosure laws.
- Review the client's SUCCESS Session Plan, if one was developed in the previous session.

Key Considerations: Inform the client about the following:

- Care and treatment services provided at your agency and/or your community partners.
- Specific requirements, such as timeliness, rescheduling policies, or paperwork required for health care providers in the area.

- Characteristics or traits of a particular clinic(s) or community partner(s) that match the client's needs. For example, a clinic with bilingual staff or interpreters for a non-English-speaking/limited-English-proficient client.
- Relevant policies (at the agency, local, state, and/or federal levels), issues, or potential barriers, such as eligibility requirements for services, Medicaid, ADAP, Ryan White services, wait lists, mandatory disclosure laws. Focus on policies with the most immediate impact on the client.

New Considerations for Session Three: Help the client:

- Identify any concerns that the client still has about linking to medical care.
- Share those concerns with you, so you know how best to assist him/her to overcome those barriers and challenges.

Procedure:

For clients who have not decided to link to medical care:

1. Ask the client about his/her expectations and concerns about seeking medical care and treatment for HIV. Be sensitive to the client's stated and unstated reasons for not wanting to seek medical treatment.
    - **This is a new step added to Session Three.** Ask if his/her concerns have changed since the beginning of SUCCESS. Begin the discussion with these questions:
        - *"What are your thoughts about linking to medical care? How, if at all, have they changed since we started working together?"*
        - *"What barriers do you think are still getting in the way? Or, do you have new barriers that you've thought of?"*

2. Continue to assess the client's tangible and perceived barriers. Remember, the client may have a multitude of personal barriers that impede his/her ability to seek services.

3. Engage the client in a discussion about medical options, provide information, and help him/her clarify concerns, issues, and barriers. Remember, it is not your role to make the decision to link to medical care for the client.

4. Demonstrate your thorough knowledge of the medical care environment and requirements and provide information based on available resources. This includes:
    - Providers and their specialties and personalities
    - How to navigate the system to apply for and access Ryan White, Medicaid, or other services
    - In other words, all the background research you did in the Pre-Implementation Section to become familiar with community partners

3E: Using SMS Technology to Enhance Linkages to Community Care for HIV+ Clients

Purpose: Encourage HIV + clients to assess medical care and maintain linkage to care, with utilization of Short Message System (SMS, i.e., text) communication technology

Forms and Documents: (n/a)

Advanced Preparation:

- Purchase and activate cell phones for use and ensure that substantial credit is on each phone

Key Considerations: Inform the client about the following:

- Uses of cell phone technology to enhance the linkage process and services provided.
- Care and treatment services provided at your agency and/or your community partners.
- Specific requirements, such as timeliness, rescheduling policies, or paperwork required for health care providers in the area.
- Characteristics or traits of a particular clinic(s) or community partner(s) that match the client's needs. (For example, a clinic with bilingual staff or interpreters for a non-English-speaking/limited-English-proficient client.)
- Relevant policies (at the agency, local, state, and/or federal levels), issues, or potential barriers, such as eligibility requirements for services, Medicaid, ADAP, Ryan White services, wait lists, mandatory disclosure laws. Focus on policies with the most immediate impact on the client.

Procedure:

For clients who have not decided to link to medical care:

1. Ask the client about his/her expectations and concerns about seeking medical care and treatment for HIV. Be sensitive to the client's stated and unstated reasons for not wanting to seek medical treatment.
   - **This is a new step added to Session Three.** Ask if his/her concerns have changed since the beginning of SUCCESS. Begin the discussion with these questions:
     - *"What are your thoughts about linking to medical care? What are your thoughts about using SMS (texting) cell phone technology to link to medical care?*
     - *"What barriers do you think are still getting in the way? Will a cell phone help with addressing any of these barriers? Are there any barriers that will get in the way of you using the cell phone to link to care?*

2. Continue to assess the client's tangible and perceived barriers. Remember, the client may have a multitude of personal barriers that impede his/her ability to seek services.

3. Engage the client in a discussion about medical options, provide information, and help him/her clarify concerns, issues, and barriers. Remember, it is not your role to make the decision to link to medical care for the client.

4. Demonstrate your thorough knowledge of the medical care environment and requirements and provide information based on available resources. This includes:
    - Providers and their specialties and personalities
    - How to navigate the system to apply for and access Ryan White, Medicaid, or other services
    - In other words, all the background research you did in the Pre-Implementation Section to become familiar with community partners

---

At this point, one of five things is likely to happen. Based on where the client is in his/her decision, follow these instructions:

If the client decides to link to medical care at this point and has not already completed the SUCCESS Session Plan, continue with Step #5.

If the client decides to link to medical care and has a SUCCESS Session Plan, skip to 3F: Review and Summarize the Session.

If the client is not ready to make this decision, skip to 3E: Review and Summarize the Session.

If the client a) wants to drop out of SUCCESS, or b) does not want to link to medical care, skip to Step #8.

For clients who wish to link to medical care at this point and have not already completed the SUCCESS Session Plan:

5. Introduce the SUCCESS Session Plan:

    "Our goal is to help you get connected to a doctor. As you may recall, we will have up to six sessions in 90 days to help you achieve this and other goals by identifying your strengths and overcoming barriers.

    The SUCCESS Session Plan is one of the activities that can guide us to accomplish your goal(s). This plan will help us organize our work together and make sure that we identify everything we need to work on. We'll write down the

goals to remind us of what we're doing and you will always have a copy of your most recent SUCCESS Session Plan, if you want it."

6. Follow the **SUCCESS Session Plan Instructions** on page 70 of the Session Forms section. The SUCCESS Session Plan helps the client identify objectives and possible barriers, activities to accomplish the objective(s), the person responsible, target dates to complete each activity, and the related strength. It is recommended that the plan be committed to in writing to allow you and the client to easily track progress and pinpoint activities that may need to be adjusted over time.

7. **Next step, continue to 3F: Review and Summarize the Session**.

For clients who want to drop out of SUCCESS or do not want to link to medical care now or in the near future (<u>if at any point the client decides to link to medical care and/or not drop out of SUCCESS, continue to 3E: Review and Summarize the Session):</u>

8. Keep the conversation positive! Cover the following topics:
   a. Engage the client in a discussion about: (1) his/her reasons for attending the previous SUCCESS sessions; and (2) his/her reasons for deciding not to continue with SUCCESS/seek medical care.
   b. Let the client know that ambiguity about linking to medical care is normal.
   c. Review the client's strengths discussed during the session.
   d. Discuss his/her accomplishments made during the session and ask how, if at all, the session has been helpful.
   e. Keep the door open. Remind the client that your sessions together can continue as long as s/he thinks it can help clarify and remove barriers to seeking treatment before the end of the 90 days.
   f. Offer the client your business card and end the session.

9. **Next step, end the session and complete paperwork**:
   - Depending on your agency's procedures and/or schedule for the day, you may want to take a few minutes to complete all the required paperwork before moving on to the next client and/or task. Recommended paperwork to complete includes:
     - Session Notes
     - Session Notes Summary Sheet (if the client dropped out)
     - Case Staffing Form

3F: Review and Summarize the Session

Purpose: To review what was discussed with the client during the session and summarize the agreed-upon next steps.

Forms and Documents: - *SUCCESS Session Plan*

Advanced Preparation: None.

Key Considerations: You should:

- Review the SUCCESS Session Plan activities and discuss/revise anything that was documented incorrectly, if a plan was developed.

Procedure: For clients who wish to link to medical care at this point, or clients who are not ready to make this decision:

1. Provide a summary of the session or ask the client to summarize the session and the client's strengths. During the session, the client may be very emotional and upset, particularly if s/he has been recently diagnosed. Therefore, summarizing the session and the client's strengths are extremely important to help the client remember the key points.

2. Review the SUCCESS Session Plan activities, person responsible, and target date to complete the items with the client, if a plan was developed. Remember, some of these are activities you are committing to complete prior to the session.

3. **Next step, continue to 3G: Schedule Medical Appointment and/or Next Session.**

3G: Schedule Medical Appointment and/or Next Session

Purpose: To schedule appointments with you, medical providers, and support services as needed.

Forms and Documents: -*SUCCESS Session Plan*
-*Resource Directory*
-*Appointment Cards*
-*Incentive, if provided*
-*Session Notes*
-*Session Notes Summary Sheet*
-*Case Staffing Form*

Advanced Preparation:

- Review your availability for the next session and/or medical appointment.
- Review the Resource Directory for medical providers, clinics, and other services as needed.
- Bring the transportation vouchers/tokens/schedules.

Key Considerations: You should:

- Refer the client to services as needed. The client may present with other needs that are related to his/her recent seroconversion or existing HIV status.
- If the client wishes to schedule a medical appointment, provide him/her with detailed information about the clinic hours and services they provide.
- Make sure all paperwork is completed and discuss how client information will be used. Stress privacy and confidentiality.
- Arrange and confirm all appointments with or for the client, medical as well as other services as needed.
- Offer to take the client or provide transportation to all scheduled appointments.
- Work on all identified barriers to following through with scheduled appointments.

Procedure:

For clients who wish to link to medical care at this point:

1. Clarify whether or not the client would like you to accompany him/her to the medical appointment.
2. Discuss the best time and date to schedule the appointment.
   - If the SUCCESS Session Plan has activities that must be completed before the medical appointment that may take some time, schedule the appointment further out or wait until the next session to schedule the medical appointment. Examples of activities that might take more time to complete are: arranging for transportation and processing Medicaid enrollment forms.
3. Call the clinic or community partner (or have the client call) to schedule an appointment.
   - **If the next time you see the client will be at the medical visit**:
     - Give him/her information about the staff and doctor, required documents.
     - Discuss in detail what the client should expect at each stage of the appointment.
     - Help the client write down questions s/he would like to ask the health care provider and/or other clinic staff. Depending on the client, practice asking and answering questions with him/her so s/he feels comfortable with the list of prepared questions.
     - Ask the client if s/he would like for you to call him/her before the medical appointment, as a reminder.

For all clients:

4. Schedule and/or make arrangements for the client to access needed social services, such as temporary housing and food banks.

5. Schedule a day, time, and meeting location for the next SUCCESS session. Make sure accommodations are compatible with agency safety guidelines.
   - If the next session is <u>before the medical appointment or the client is not linking to medical care at this point</u>, offer to write down these details and to call the client

before your next session as a reminder. The topics covered will follow the format for **Session Four**.

- If it is scheduled for after the medical appointment, the next session will be **Session Six** (completing the work with the client).

6. Offer the client an **appointment card** (see samples on page 79 of the Session Forms section) to document the time, location, and agency name.

7. Give the client transportation tokens or vouchers to get home and/or to the next session or appointment. Or offer to pick the client up, if that is an allowable activity at your agency.

8. Gather any contact and/or locator information from the client before s/he leaves. Locator information will allow you to locate the client through family, friends, or other individuals who know how to reach him/her if the client's address changes, phone is disconnected, or the client is not reachable through the means provided in the initial intake (conducted within the agreed-upon rules for communicating with the client). Remind the client that you will attempt to contact him/her through these means only after a missed appointment.

    - To gather this information, discuss how the locator information will be used and be sure to inform the client that none of his/her personal information will be shared with the contacts provided. Information collected from the contact persons includes the following: usual place of residence; telephone number or address of someone who usually knows where the client can be found; places where s/he picks up mail or messages.
    - Ask the client if the contacts are aware of his/her HIV status and assure the client that his/her contacts will not be told the reason for the call. You can say you are a friend trying to reach the client.

9. End the session by thanking the client for coming and congratulate him/her for a productive session. Remind the client that linking to medical care is important to his/her overall health, and that you are there to help him/her attain services needed so that s/he is ready to access medical care and treatment.

10. **Next step, complete paperwork**: • Depending on your agency's procedures and/or schedule for the day, you may want to take a few minutes to complete all the required paperwork before moving on to the next client and/or task. Recommended paperwork to complete includes:
    - ○ Session Notes
    - ○ Session Notes Summary Sheet (if the client dropped out)
    - ○ Case Staffing Form

SESSION FOUR: STRENGTHENING LINKAGES

Approximately: 1 ½ - 2 hours

Session Four Activities

A: Solicit client issues and questions from Session Three
B: Initiate the transition process
C: Continue identifying personal strengths, abilities, and skills
D: Encourage linkage to care/Identify and address barriers to linkage
E: Summarize the session, the client's strengths, and agreed-upon next steps
F: Plan for the next session(s), with the medical care provider and/or you

Session Three Guide Agenda

4A. Review of Session Three
4B. Transition Planning
4C. Client Assessment
4D. Linkage to Medical Care
4E. Review and Summarize the Session
4F. Schedule Medical Appointment and/or Next Session

Forms and Documents Needed for Session Three:

- *SUCCESS Session Plan*
- *Strengths Assessment*
- *Resource Directory*
    - *A Listing of Medical and Psychiatric Service Providers and Local Social Service Providers (e.g., housing, food, insurance)*
- *Appointment Cards*
- *Incentive, if provided*
- *Session Notes*
- *Session Notes Summary Sheet*
- *Case Staffing Form*
- *Life Domains List*

Note: As noted throughout this manual, the transition process begins with the very first contact. However, during the fourth client session, the time-limited nature of the relationship should become particularly relevant and explicitly addressed. Many of the activities listed below are similar to the activities in previous sessions. However, there are a few slight variations to the step-by-step procedures listed. The variations are a result of this being the fourth session conducted; you may need to expand on specific points to clarify why the client is still facing the same barriers, not deciding to link to medical care, or to continue to identify strengths.

Remember: The sessions are client-driven. As such, the agenda, time, content, and forms must be adjusted to the client's needs.

4A: Review of Session Three

Purpose: To clarify and address any questions or areas of confusion the client has from Session Three.

Forms and Documents:
- *SUCCESS Session Plan*

Advanced Preparation:
● Review the client's SUCCESS Session Plan, if one was developed in the previous session.

Key Considerations: Remember that the client:
● Needs support and resources to effectively link to medical care. Be sure to review the client's needs and refer him/her to needed services to assist in accessing medical care.
● Often needs assistance to identify personal strengths and abilities to facilitate his/her linkage to medical care.
● May need to reflect on his/her HIV status and barriers encountered in disclosing his/her status to others and in accessing social services.

Procedure:
For all clients:

1. Welcome the client back for Session Four and congratulate him/her on following up successfully with today's session. Recognize the many demands the client has and state how much you appreciate him/her taking time to meet with you.

2. Ask the client what questions, concerns, or new insights s/he has as a result of the previous sessions. You may also want to ask about his/her thoughts about linking to medical care since your last session and any reactions s/he has to the focus on strengths, which will help you to assess whether or not s/he is starting to adopt the new approach.

3. Summarize any additional points made during the discussion.

4. **Next step, continue to 4B: Transition Planning.**

4B: Transition Planning

Purpose: To plan for and review the transition process between you and the client. **You will not actually disengage from the client at this time. This activity serves as a reminder for you and the client that there are only two sessions remaining after today's session.**

Advanced Preparation: None.

Key Considerations: Know that the client:

- May be anxious due to problems s/he experienced when addressing barriers and/or accessing social services.
- May think if s/he identifies more barriers, s/he can continue to meet with you after the sixth session.

Procedure:
For all clients:

1. Emphasize the time-limited nature (only six sessions or within 90 days, whichever happens first) of SUCCESS and that only two additional sessions remain after today's session. Be prepared for possible resistance from the client.

2. Review past sessions with the client by focusing on:
   - His/her strengths
   - Concrete examples of barriers the client overcame to attend the SUCCESS sessions and/or access other social services
   - His/her plans for linking to medical care (with or without you)

3. Normalize any fear or ambivalence the client may have about linking to medical care without your direct support.

4. Help the client identify valuable things about the work s/he has done on his/her own, and the work the two of you have done together. Ask the client how s/he thinks these things can be applied to linking with medical care or other services. Some sample questions include:
   - *"What do you think about the strengths you've identified in the last three sessions?*
   - *"How are you better prepared to visit a doctor, when you choose to do so – as compared to when we first started working together?"*
   - *"What barriers did you work on so far? How can you apply that to other barriers?"*

5. If you think the client will likely need a long-term/Ryan White case manager to access other resources and services after SUCCESS, formally or informally introduce the client to the new case manager with whom s/he will be working.

For clients who have not decided to link to medical care:

6. **Next step, continue to 4C: Client Assessment.**

For clients who have decided to link to medical care but have not yet attended their appointment:

7. Review the outcomes of all activities listed on the SUCCESS Session Plan for both you and the client. If necessary, revise the plan.

8. Ask the client about any new barriers and/or strengths discovered as a result of completing the SUCCESS Session Plan activities. If necessary, revise the plan.

9. **Next step, skip to section 4E: Review and Summarize the Session.**

4C: Client Assessment

Purpose: To continue to help the client self-identify personal strengths, abilities, and skills.

Forms and Documents:
- *Resource Directory*
- *Strengths Assessment*

Advanced Preparation:
- Review the state/local legal requirements regarding HIV disclosure.
- Review the client's Strengths Assessment from the previous session.

Key Considerations: You should:
- Use effective communication skills.
- Know the state/local legal requirements regarding disclosing one's HIV status.
- Have natural conversations with the client to identify additional strengths.
- Ask open-ended questions that encourage the client to provide more substantive information to build on the list of strengths developed in Session One.

- Show the client genuine respect and concern, as this is the starting point of a helping relationship.

Procedure:
For clients who have not yet decided to link to medical care:

1. Remind the client how identifying strengths, abilities, and skills relate to his/her ability to stay healthy and link to medical care. The explanation could be as follows:

    "Often times, when you see ways that you've been successful in the past, it helps you to be successful again. Knowing how you've been successful helps you plan how to deal with barriers or problems you may have getting the medical care you need or achieving other goals."

2. Remind the client of examples of his/her strengths and abilities that have already become apparent in the previous sessions. This will help the client think about personal strengths, resources, and skills.

3. Conduct the **Strengths Assessment** by following the instructions and introduction script starting on page 64 in the Session Forms section. Take the time to really understand what the client's strengths and abilities are, as well as to help the client identify more of his/her positive attributes. Use open-ended questions to stimulate a discussion with the client. Some sample questions include:
    - *"What strengths have we talked about that you hadn't thought of before?"*
    - *"Give me an example of a recent experience where you successfully overcame a barrier, and tell me what you did."*
    - *"Tell me some of the things that you, or other people who know you well, would say you are good at."*
    - *"Think about a time that you felt like most things were going well. What were you doing to make them go well?"*

4. **Next step, continue to 4D: Linkage to Medical Care.**

4D: Linkage to Medical Care

Purpose: Encourage the client to seek medical care, and if interested, assist him/her in the process to make that linkage.

Forms and Documents: - *SUCCESS Session Plan*
- *Resource Directory*

- *Session Notes*
- *Session Notes Summary Sheet*
- *Case Staffing Form*

Advanced Preparation:

- Review any specific requirements, characteristics/traits, agency policies, and required paperwork of the health care providers.
- Review any local, state, or federal policies such as eligibility requirements for services, Medicaid, ADAP, Ryan White services, wait lists, mandatory disclosure laws.
- Review the client's SUCCESS Session Plan, if one was developed in the previous session.

Key Considerations: Inform the client about the following:

- Care and treatment services provided at your agency and/or your community partners.
- Specific requirements, such as timeliness, rescheduling policies, or paperwork required for health care providers in the area.
- Characteristics or traits of a particular clinic(s) or community partner(s) that match the client's needs. For example, a clinic with bilingual staff or interpreters for a non-English-speaking/limited-English-proficient client.
- Relevant policies (at the agency, local, state, and/or federal levels), issues, or potential barriers, such as eligibility requirements for services, Medicaid, ADAP, Ryan White services, wait lists, mandatory disclosure laws. Focus on policies with the most immediate impact on the client.

New Considerations for Session Four:

Help the client:

- Identify any concerns that the client still has about linking to medical care.
- Share those concerns with you, so you know how best to assist him/her to overcome those barriers and challenges.

Procedure:

For clients who have not decided to link to medical care:

1. Ask the client about his/her expectations and concerns about seeking medical care and treatment for HIV. Be sensitive to the client's stated and unstated reasons for not wanting to seek medical treatment. Ask if his/her concerns have changed since the beginning of SUCCESS. Begin the discussion with these questions:

• *"What are your thoughts about linking to medical care? How, if at all, have they changed since we started working together?"*

• *"What barriers do you think are still getting in the way? Or, do you have new barriers that you've thought of?"*

2. Continue to assess the client's tangible and perceived barriers. Remember, the client may have a multitude of personal barriers that impede his/her ability to seek services.
3. Engage the client in a discussion about medical options, provide information, and help him/her clarify concerns, issues, and barriers. Remember, it is not your role to make the decision to link to medical care for the client.
4. Demonstrate your thorough knowledge of the medical care environment and requirements and provide information based on available resources. This includes:
- Providers and their specialties and personalities
- How to navigate the system to apply for and access Ryan White, Medicaid, or other services
- In other words, all the background research you did in the Pre-Implementation Section to become familiar with community partners

---

At this point, one of five things is likely to happen. Based on where the client is in his/her decision, follow these instructions:

If the client decides to link to medical care at this point and has not already completed the SUCCESS Session Plan, continue with Step #5.

If the client decides to link to medical care and has a SUCCESS Session Plan, skip to 4E: Review and Summarize the Session.

If the client is not ready to make this decision, skip to 4E: Review and Summarize the Session.

If the client a) wants to drop out of SUCCESS, or b) does not want to link to medical care, skip to Step #8.

For clients who wish to link to medical care at this point and have not already completed the SUCCESS Session Plan:

5. Introduce the SUCCESS Session Plan:

   "Our goal is to help you get connected to a doctor. As you may recall, we will have up to six sessions in 90 days to help you achieve this and other goals by identifying your strengths and overcoming barriers."

   "The SUCCESS Session Plan is one of the activities that can guide us to accomplish your goal(s). This plan will help us organize our work together and make sure that we identify everything we need to work on. We'll write down the goals to remind us of what we're doing and you will always have a copy of your most recent SUCCESS Session Plan, if you want it."

6. Follow the **SUCCESS Session Plan Instructions** on page 70 of the Client Session Guide Forms section. The SUCCESS Session Plan helps the client identify objectives and possible barriers, activities to accomplish the objective(s), the person responsible, target dates to complete each activity, and the related strength. It is recommended that the plan be committed to in writing to allow you and the client to easily track progress and pinpoint activities that may need to be adjusted over time.

7. **Next step, continue to 4E: Review and Summarize the Session.**

For clients who want to drop out of SUCCESS or do not want to link to medical care now or in the near future (<u>if at any point the client decides to link to medical care and/or not drop out of SUCCESS, continue to 4E: Review and Summarize the Session</u>):

8. Keep the conversation positive! Cover the following topics:
    a. Engage the client in a discussion about: (1) his/her reasons for attending the previous SUCCESS sessions; and (2) his/her reasons for deciding not to continue with SUCCESS/seek medical care.
    b. Let the client know that ambiguity about linking to medical care is normal.
    c. Review the client's strengths discussed during the session.
    d. Discuss his/her accomplishments made during the session and ask how, if at all, the session has been helpful.
    e. Keep the door open. Remind the client that your sessions together can continue as long as s/he thinks it can help clarify and remove barriers to seeking treatment before the end of the 90 days.
    f. Offer the client your business card and end the session.

9. **Next step, end the session and complete paperwork**:
    - Depending on your agency's procedures and/or schedule for the day, you may want to take a few minutes to complete all the required paperwork before moving on to the next client and/or task. Recommended paperwork to complete includes:
        - Session Notes
        - Session Notes Summary Sheet (if the client dropped out)
        - Case Staffing Form

4E: Review and Summarize the Session

Purpose: To review what was discussed with the client during the session and summarize the agreed-upon recommendations and next steps.

Forms and Documents: - *SUCCESS Session Plan*

Advanced Preparation: None.

Key Considerations: You should:
● Review the SUCCESS Session Plan activities and discuss/revise anything that was documented incorrectly, if a plan was developed.

Procedure:
For clients who wish to link to medical care at this point, or clients who are not ready to make this decision:

1. Provide a summary of the session or ask the client to summarize the session and the client's strengths. During the session, the client may be very emotional and upset, particularly if s/he has been recently diagnosed. Therefore, summarizing the session and the client's strengths are extremely important to help the client remember the key points.

2. Review the SUCCESS Session Plan activities, person responsible, and target date to complete the items with the client, if a plan was developed. Remember, some of these are activities you are committing to complete prior to the session.

3. **Next step, continue to 4F: Schedule Medical Appointment and/or Next Session.**

4F: Schedule Medical Appointment and/or Next Session

Purpose: To schedule appointments with you, medical providers, and support services as needed.

Forms and Documents: - *SUCCESS Session Plan*
- *Resource Directory*
- *Appointment Cards*
- *Incentive, if provided*
- *Session Notes*
- *Session Notes Summary Sheet*
- *Case Staffing Form*

Advanced Preparation:
● Review your availability for the next session and/or medical appointment.
● Review the Resource Directory for medical providers, clinics, and other services as needed.

• Bring the transportation vouchers/tokens/schedules.

Key Considerations: You should:

• Refer the client to services as needed. The client may present with other needs that are related to his/her recent seroconversion or existing HIV status.

• If the client wishes to schedule a medical appointment, provide him/her with detailed information about the clinic hours and services they provide.

• Make sure all paperwork is completed and discuss how client information will be used. Stress privacy and confidentiality.

• Arrange and confirm all appointments with or for the client, medical as well as other services as needed.

• Offer to take the client or provide transportation to all scheduled appointments.

• Work on all identified barriers to following through with scheduled appointments.

Procedure:

For clients who wish to link to medical care at this point:

1. Clarify whether or not the client would like you to accompany him/her to the medical appointment.
2. Discuss the best time and date to schedule the appointment.
   - If the SUCCESS Session Plan has activities that must be completed before the medical appointment that may take some time, schedule the appointment further out or wait until the next session to schedule the medical appointment. Examples of activities that might take more time to complete are: arranging for transportation and processing Medicaid enrollment forms.
3. Call the clinic or community partner (or have the client call) to schedule an appointment.
   - **If the next time you see the client will be at the medical visit**:
     - Give him/her information about the staff and doctor, required documents.
     - Discuss in detail what the client should expect at each stage of the appointment.
     - Help the client write down questions s/he would like to ask the health care provider and/or other clinic staff. Depending on the client, practice asking and answering questions with him/her so s/he feels comfortable with the list of prepared questions.
     - Ask the client if s/he would like for you to call him/her before the medical appointment, as a reminder.

For all clients:

4. Schedule and/or make arrangements for the client to access needed social services, such as temporary housing and food banks.

5. **This is a new step added to Session 4.** Remind him/her you have two more sessions together. If you think the client will have a difficult time with the transition process, review 4B: Transition Planning again.

6. Schedule a day, time, and meeting location for the fifth SUCCESS session. Make sure accommodations are compatible with agency safety guidelines.
   - If the next session is <u>before the medical appointment or the client is not linking to medical care at this point</u>, offer to write down these details and to call the client before your next session as a reminder. The topics covered will follow the format for **Session Five**.
   - If it is scheduled for <u>after the medical appointment</u>, the next session will be **Session Six** (completing the work with the client).

7. Offer the client an **appointment card** (see samples on page 79) to document the time, location, and agency name.

8. Give the client transportation tokens or vouchers to get home and/or to the next session or appointment. Or offer to pick the client up, if that is an allowable activity at your agency.

9. Gather any contact and/or locator information from the client before s/he leaves. Locator information will allow you to locate the client through family, friends, or other individuals who know how to reach him/her if the client's address changes, phone is disconnected, or the client is not reachable through the means provided in the initial intake (conducted within the agreed-upon rules for communicating with the client). Remind the client that you will attempt to contact him/her through these means only after a missed appointment.
   - To gather this information, discuss how the locator information will be used and be sure to inform the client that none of his/her personal information will be shared with the contacts provided. Information collected from the contact persons includes the following: usual place of residence; telephone number or address of someone who usually knows where the client can be found; places where s/he picks up mail or messages.
   - Ask the client if the contacts are aware of his/her HIV status and assure the client that his/her contacts will not be told the reason for the call. You can say you are a friend trying to reach the client.

10. End the session by thanking the client for coming and congratulate him/her for a productive session. Remind the client that linking to medical care is important to his/her overall health,

and that you are there to help him/her attain services needed so that s/he is ready to access medical care and treatment.

11. Next step, complete paperwork:
    - Depending on your agency's procedures and/or schedule for the day, you may want to take a few minutes to complete all the required paperwork before moving on to the next client and/or task. Recommended paperwork to complete includes:
        - Session Notes
        - Session Notes Summary Sheet (if the client dropped out)
        - Case Staffing Form

SESSION FIVE: REVIEWING PROGRESS

Approximately: 1 ½ - 2 hours

Session Five Activities

A: Review the transition process for clients linked to medical care

B: Review the transition process for clients not yet linked to medical care

Session Five Guide Agenda

5A. Review the Transition Process: Linked Clients

OR

5B. Review the Transition Process: Non-linked Clients

Forms and Documents Needed for Session Five:

- *SUCCESS Session Plan*
- *Resource Directory*
- *Contact information for long-term/Ryan White case manager and agency*
- *Paperwork for long-term/Ryan White case manager*
- *Session Notes*
- *Session Notes Summary Sheet*
- *Case Staffing Form*
- *Client Satisfaction Questionnaire*

Note: For Session Five, you do not conduct all both agenda items for each client. **For clients linked to medical care, conduct agenda items 5A then move on to Session Six.**

For clients who have not linked to medical care (non-linked clients), conduct agenda item 5B then move on to Session Six.

Remember: The sessions are client-driven. As such, <u>the agenda, time, content, and forms must be adjusted to the client's needs.</u>

5A: Review the Transition Process – Linked Clients

Purpose: To review the client's progress made during SUCCESS and discuss the client's visit with the medical provider.

Forms and Documents: - *SUCCESS Session Plan*
- *Resource Directory*
    - *List of Community Service Providers (e.g., substance abuse, mental health, housing, food, and insurance)*

Advanced Preparation:
- Review the client's SUCCESS Session Plan, if one was developed in the previous session.

Key Considerations: Remember that the client:
- May not be ready to transition from SUCCESS and/or end your relationship.

Procedure:

For all clients:

1. Welcome the client back for the last session and congratulate him/her on following up successfully with today's session. Recognize the many demands the client has and state how much you appreciate him/her taking time to meet with you.

2. Ask the client what questions, concerns, or new insights s/he has as a result of the previous session(s).

3. Summarize any additional points made during the discussion.

For clients who have decided to link to medical care but have not yet attended their appointment:

4. Review the outcomes of all activities listed on the SUCCESS Session Plan for both you and the client. If necessary, revise the plan.

5. Ask the client about any new barriers and/or strengths discovered as a result of completing the SUCCESS Session Plan activities. If necessary, revise the plan.

6. **This is a new step added to Session Five.** Discuss how the client can continue to use this plan to achieve his/her goals after SUCCESS.

7. **This is a new step added to Session Five.** Complete the steps listed under **4F: Schedule Medical Appointment and/or Next Session.** Then, skip to Step #9 in this section.

For clients who attended a medical appointment:

8. Discuss the client's appointment with the medical provider, including his/her reactions and any questions s/he may have. Review with the client what happened during the medical visit and ask what the client thought went well and what could be improved.

For all clients:

9. Discuss the barriers that the client identified and overcame during SUCCESS. Review strategies that s/he identified as successful. Point out any additional strategies that you noticed that s/he may not have noted.

10. Discuss any remaining barriers that could interfere with the client attending his/her next medical appointment or linking to other support services. Strategize with the client to identify ways that s/he can overcome these. If there are items in the SUCCESS Session Plan that the client has yet to complete, obtain a commitment from him/her that s/he will continue to work on these.

11. Ask the client what questions, concerns, or insights s/he has now that s/he completed the intervention. Address any additional issues that arise.

12. Encourage self-help through HIV support groups and linkage to long-term social services. Review the community resources discussed during earlier sessions. Also review the important role the client's family, friends, social groups, and other informal networks can play in supporting his/her continued use of medical care and other services. Provide verbal and written information regarding community services available.

13. **Next step, skip to 6A: Transition to long-term/Ryan White case manager or other providers.**

5B: Review the Transition Process – Non-linked Clients

Purpose: To review the client's progress made during SUCCESS and discuss how the client will link to medical care.

Forms and Documents: - *SUCCESS Session Plan*
- *Resource Directory*
  - ○ *List of Medical Providers*
  - ○ *List of Community Service Providers (e.g., substance abuse, mental health, housing, food, and insurance)*
- *Paperwork for long-term/Ryan White case management*

Advanced Preparation:

● Review the client's SUCCESS Session Plan, if one was developed in the previous session.

Key Considerations: Remember that the client:

● May not be ready to transition from SUCCESS and/or end your relationship.
● May feel discouraged or that s/he has failed by not linking to medical care during SUCCESS.

Procedure:
For all non-linked clients:

1. Welcome the client back for the last session and congratulate him/her on following up successfully with today's session. Recognize the many demands the client has and state how much you appreciate him/her taking time to meet with you.

2. Ask the client what questions, concerns, or new insights s/he has as a result of the previous session(s). You may also want to ask about the client's thoughts about linking to medical care since your last session and any reactions s/he has to the focus on strengths.

3. Summarize any additional points made during the discussion.

4. Using the SUCCESS Session Plan, review the client's progress over the course of the intervention. Discuss the client's strengths and how s/he used these to complete the tasks listed in his/her plan. Emphasize the client's accomplishments during SUCCESS.

5. Discuss the barriers that the client identified and overcame during SUCCESS. Review strategies that s/he identifies as being successful. Point out any additional strategies that you have noticed s/he may not have noted.

6. Discuss the client's hesitance to link to medical care. Review the psychological and/or physical barriers that are preventing the client from accessing medical care. Discuss with the client how s/he can overcome these barriers. If the client desires, revise the SUCCESS Session Plan to reflect concrete steps s/he can take, post-SUCCESS, to link to medical care.

7. Remind the client about the benefits of early entry into medical care. Provide him/her with contact information for community medical providers and promote the client's independent contact with the clinic. Offer him/her the opportunity to call you one additional time following his/her independent clinic visit.

8. Ask the client what questions, concerns, or insights s/he has now that s/he completed the intervention. Address any additional issues that arise.

9. Encourage self-help and linkage to medical and long-term social services. Review the community resources discussed during earlier sessions. Also review the important role the client's family, friends, social groups, and other informal networks can play in supporting his/her linkage to medical care and other services. Provide verbal and written information regarding other community services available.

10. **Next step, continue to 6A: Transition to long-term/Ryan White case manager or other providers.**

SESSION SIX: COMPLETING THE WORK/FUTURE TRANSITIONS

Approximately: 1 ½ - 2 hours

**Session Six Activities**
A: Transition to long-term/Ryan White case management or other providers

6A: Transition to long-term/Ryan White case manager

Purpose: Explain to the client the purpose of long-term/Ryan White case management services and how it differs from SUCCESS. Facilitate the transition to the new case manager.

Forms and Documents: - *Contact information for long-term/Ryan White case manager*
- *Session Notes*
- *Session Notes Summary Sheet*
- *Case Staffing Form*
- *Client Satisfaction Questionnaire*

Advanced Preparation:

- Ask the new long-term/Ryan White case manager to be available during the client session so s/he can meet the client.
- Bring the name and contact information of the long-term/Ryan White case manager.

Key Considerations: Remember:

- The client may be unsure about what to expect from long-term/Ryan White case management or the new case manager.
- The client may be hesitant to connect with a new case manager.
- To ask the long-term/Ryan White case manager to join the session, if the client agrees.

Procedure:
For all clients:

1. Explain what the client can and cannot expect from long-term/Ryan White case management and how it differs from SUCCESS, as follows:
    a. While SUCCESS focused mainly on overcoming short-term barriers to linking to medical care, the long-term/Ryan White case manager can work with the client on more general issues such as housing, employment, other treatment needs.

   b. The relationship with the long-term/Ryan White case manager will not be as intensive as his/her relationship with you. As a result, the case manager may not be able to accompany the client to appointments.
   c. The relationship between the client and long-term/Ryan White case manager will not be restricted to 90 days or six sessions.
   d. The client will still be expected to actively participate in his/her care.

2. Emphasize how the client can use the strengths identified during his/her participation in SUCCESS to overcome barriers to services provided by the new case manager. Validate the client's concerns by saying: *"This kind of case management is different and you won't be working with me. But you can have a similar working relationship with your new case manager."*

3. Answer any questions and address any concerns the client has about this new form of case management.
4. Ask the client if s/he would be open to having the long-term/Ryan White case manager join the session, if s/he is available. Note: <u>Plan in advance with the new case manager and ensure s/he is available.</u>
   a. <u>If the client would like to meet the new case manager,</u> bring him/her into the session and introduce them to each other. Ask the client to tell his/her story, share the work s/he has done in SUCCESS, and the strengths s/he has identified. Ask the client to discuss the barriers that s/he identified through SUCCESS and what s/he has done to overcome them. Review any other barriers to accessing medical care or support services that will need to be addressed. Discuss any other issues that have arisen during SUCCESS that the client will need to address during long-term/Ryan White case management.
   b. <u>If the client is not comfortable having the long-term/Ryan White case manager join the session</u>, discuss his/her reluctance. Discuss how the client will access case management on his/her own and how s/he can overcome barriers or discomfort to doing so. Review the benefits of case management and what the client can gain from participating. Provide the client with the contact information for his/her long-term/Ryan White case manager, and ask his/her permission to give his/her contact information to the new case manager.

5. Complete all paperwork necessary to transfer the client to another agency and/or case manager, if this is in the MOA between your agency and community partner. This could include discharge forms for your agency, intake/referral forms for the partner agency, and updates on client progress/status.

6. Thank the client for coming and congratulate him/her for completing the intervention and working with you. Remind the client that linking to medical care is important to his/her overall health, and that you hope s/he uses the skills you talked about to obtain services needed so s/he can access medical care and treatment.

7. End the session by asking the client to complete the **Client Satisfaction Questionnaire**, on page 92 in the Session Forms section.

8. **Next step, end the session and complete paperwork**:
   ● Depending on your agency's procedures and/or schedule for the day, you may want to take a few minutes to complete all the required paperwork before moving on to the next client and/or task. Recommended paperwork to complete includes:
   ○ Session Notes
   ○ Session Notes Summary Sheet (if the client dropped out)
   ○ Case Staffing Form

Session Forms

This section contains all the forms and additional document you will use to conduct each client session. Each form or document is referenced throughout the Client Session Guide in bold and includes the page number where the form/document can be found in this section. Before each form, you will find the instructions on how to introduce and/or use it with the client.

Table of Contents

Strengths Assessment	Page 64
SUCCESS Session Plan	Page 70
Overview of SUCCESS Document	Page 72
Sample Fact Sheet on Current Treatment Options and their Side Effects	Page 73
Appointment Cards	Page 79
Session Notes	Page 83
Session Notes Summary Sheet	Page 85
Case Staffing Form	Page 88
Life Domains List	Page 90
SUCCESS Client Satisfaction Questionnaire	Page 93

©2017 Center for Health of Incarcerated Persons, Rollins SPH, Emory University

Strengths Assessment Instructions

1. Clearly introduce the intent of the Strengths Assessment. The exact introduction you choose should be tailored to your personal style and the client's reading level or cognitive ability. Below is a sample introduction to the Strengths Assessment process:

   "One of the activities that we will complete together to help you identify your needs is a Strengths Assessment. This assessment is different from past assessments that you may have completed with another case manager. The design of this assessment helps you to recognize your strengths, skills, abilities, and things that you're good at doing. We have found that when people can recognize what they are good at, it helps them accomplish new or difficult goals. By recognizing areas where you've been successful, you can use those examples to put you in a better position to accomplish your personal goals and to take the necessary steps to seek treatment. You may already be aware of these strengths. Or, they may be things you haven't thought about for a long time or things you've never thought about.

   Some people find it hard at first to focus on their personal strengths because most of us were taught that it is bragging to talk about what we've done right. I don't think that at all. I think focusing on our personal strengths reminds each of us how we all have talents and abilities that help us do what we need to do for ourselves."

2. Choose one of two options to complete the assessment with a client. Both options accomplish the same things: building effective relationships, gathering information, and engaging the client.

   Option 1: Simply talk to the client about his/her life. This option occurs in a natural, but guided, conversation designed to help the client think about and identify strengths and abilities. Pick up on the stories told that reflect the

client's *abilities*. Summarize or use open-ended questions to encourage clients to talk about positive rather than negative experiences. For example, *"Earlier you noted something about a job at the corner market. Tell me more about what you did to find that job and to get hired."*

OR

Option 2: Start the conversation about strengths using very general, but direct questions, such as:

- *What strengths do you think you have?*
- *What are your abilities?*
- *When have you successfully faced barriers, and what did you do to overcome them?*
- *What are you good at?*
- *Tell me about a time when you felt like most things were going well. What were you doing to make them go well?*

3. During the conversation (regardless of which option you choose), listen for examples where the client identifies his/her strengths. Focus on what the client says, and remember these examples by using reflection, summarizing, and affirmations to reinforce the ideas for you and the client (if necessary, jot down a quick note). This is important because many of the ideas and examples will apply to the client's goals.

4. Make a list of the strengths, abilities, and skills identified by the client in his/her stories during the conversation. Use the client's own words. What is most important is giving the client an opportunity to see – in writing – a list of his/her personal, positive attributes. Therefore, you can choose a format for the assessment that suits your agency. A suggested format for the **Strengths Assessment** is on page 64.

5. Copy the list for the client, if s/he would like to take it home.

While the above Strengths Assessment exercise is designed to solicit examples of previous successes from the client, you should never view the assessment process as static. That is to say, it is an ongoing process, rather than a one-time, discrete activity. Because the intervention is client-driven, a client may not be ready to share his/her personal stories immediately or may not be able to share strengths right away. S/he will choose to share on his/her terms. Therefore, it is important for you to continually search for strengths, skills, and abilities during each client session, brief phone call, general conversation, or other contact with the client. By doing so, you provide an opportunity for the client to choose the right time to share and to help the client see the day-to-day presence and connection of his/her strengths.

Collecting Information that is not Strengths-Based: While the emphasis of SUCCESS is identifying strengths and abilities, it is always appropriate and necessary to incorporate sound clinical practice into each session. Therefore, it is also essential to collect information that is not strengths-based. Examples of non-strengths information that must be collected include:

- Suicidal ideation or attempts

• Risk to do harm to others
• Physical problems associated with substance abuse, including overdose risk, delirium tremens, or drug withdrawal
• Inherent limitations, such as not being able to read, having a learning disability, or having physical impairments that may affect the client's ability to link to medical care

By having knowledge of and sensitivity to inherent limitations, you will be able to identify valuable resources for the client.

When collecting this non-strengths information, you should remember to treat the client as an individual and never as a member of a group with problems.

Additional key points to remember when conducting the Strengths Assessment are listed below.

- Believe in the power of strengths and abilities, and believe that every client possesses strengths and abilities. Many clients, because of their previous contact with services providers, are adept at spotting someone who is being phony, condescending, or patronizing.
- From time to time, it may be necessary to gently refocus a client on his/her strengths and away from a discussion of problems and deficiencies. A Strengths Assessment stands out as a *significantly different approach to addressing a client's needs*, as many SUCCESS clients have confronted numerous negative events in their lives.
- Remind yourself and your clients that important problems are not being ignored by completing a Strengths Assessment. More accurately, the focus on strengths and abilities prepares the client to deal with barriers to accessing medical care and other challenges s/he might face.
- Be careful about reaching too far to find strengths. For example, suggesting to a client: "*You've been a successful sex worker. Let's talk about your strengths in that area*." While this situation includes some strengths, such as negotiating skills around price, the emphasis should on the specific characteristic – being resourceful – and not on the larger role – being a sex worker. Encourage clients to identify how these characteristics can be readily adapted to a healthier lifestyle.
- Emphasize *the client's role* in making things go right and help him/her explore how s/he personally influenced the positive outcome. A client may attempt to give someone else the credit for his/her strengths and/or for times when things were going well.
- Often you will hear a client discuss certain actions but then not directly describe them as strengths. If you think those actions, thoughts, or feelings are strengths, use responsive listening techniques to encourage the client to consider them as such. Ultimately it is your client's perceptions of something as a positive in his/her life that will enable him/her to mobilize to solve current problems/barriers.

- Periodically summarize strengths that have been identified by the client. This will help him/her identify patterns that exist.
- Avoid acting as an investigator. It is better to assume a facilitator role in the search for abilities.
- Keep the goal of linkage to care as an honest part of the Strengths Assessment and all discussions. Do not try to covertly or overtly steer the client in a desired direction.

©2017 Center for Health of Incarcerated Persons, Rollins SPH, Emory University

STRENGTHS ASSESSMENT FORM

Linkage Coordinator's Copy

Client ID: ____

LC's Name: ____

Date: ____________________________**Session Number:** _______________________________

Is this the 1st assessment completed for the client or is it an amendment? __________________

1. **What strengths, abilities, or skills did the client identify (either directly or indirectly)?**
    a. Strengths:
    b. Skills:
    c. Abilities:
    d. Which items from the Life Domains List, if needed, prompted the client?

2. **What examples did the client give about a time when s/he successfully faced barriers? a.** What did s/he do to overcome the barrier(s)?
3. **What did the client explicitly say s/he was good at?**

4. **What did the client implicitly say s/he was good at, i.e., what did you hear him/her say? a.** Did the client agree with what you heard as something s/he is good at once you repeated it back?

5. **What example(s) did the client give about a time/experience when s/he felt like most things were going well in his/her life? What was s/he doing to make them go well?**

©2017 Center for Health of Incarcerated Persons, Rollins SPH, Emory University

STRENGTHS ASSESSMENT FORM

Client's Copy

Date: ___________________________
Session Number: _________________
New Assessment or Updated Assessment? (Circle one)

1. **My strengths, abilities, or skills identified:**
   a. Strengths:
   b. Skills:
   c. Abilities:
   d. Items from the Life Domains List:

2. **Examples I gave about a time(s) that I successfully faced barrier(s) in my life:**
   a. Examples of barrier(s):
   b. Things I did to overcome the barrier(s):

3. **Things I am good at:**

4. **Example(s) of when I felt like most things in my life were going well:**
   a. Things I did to make them go well:

©2017 Center for Health of Incarcerated Persons, Rollins SPH, Emory University

SUCCESS Session Plan Instructions

Introduce the SUCCESS Session Plan in a way that demonstrates how easy it is to use and provides a way for the client to maintain ownership of the plan.

While the format is less important than the content captured, a sample form to use as a guide for the **SUCCESS Session Plan** is on page 70. It is useful for the plan to be organized by: objectives, activities, related strengths, potential barriers, person responsible, and target dates for each objective and activity.

► **Identifying Goals**: In keeping with a strengths-based perspective, all goals should be a reflection of the client's wishes, not your or the agency's wishes. Take great care not to impose your own goals on a client. Even the goal of linking to medical care should not be imposed on the client. While you advocate for linking medical care, you must be careful not to force this goal or any others onto the client. Advocate for the linkage goal without interfering with the client's other goals, as long as they do not conflict with the Core Elements of SUCCESS.

Goals are written as broad statements, and always in the client's exact words. Using a client's own words decreases the distance between the client and the goal, and places the responsibility for accomplishing the goal squarely on the client. Further, it eliminates the possibility that you inadvertently alter the goal to something you believe is more important. In the end, the client must embrace his/her goals if s/he is to be successful.

► **Creating Objectives and Activities**: Objectives will be appropriate and effective if you follow the SMART technique for writing objectives. The components of a SMART objective are: Specific, Measurable, Achievable, Relevant, and Time-bound.

Activities are the smaller steps to accomplish a client's objectives. Below is an example of a client goal, the objectives, and the activities s/he needs to complete to accomplish his/her goal. The establishment of target dates for each objective and activity allows for periodic review of the client's progress and the opportunity to make adjustments as necessary.

GOAL: Find a job I enjoy.

OBJECTIVE 1: Take the GED exam by the end of the year.

> **ACTIVITIES**: (1) Obtain a GED application by April 1st; (2) Study a GED work guide ten hours each week from April 1st to June 30th; and (3) Schedule an appointment to take the GED exam in July.

OBJECTIVE 2: Complete a course on identifying job interests at Smith Vocational School by August 1st.

> **ACTIVITIES:** (1) Identify courses available, the dates, and the cost; (2) Save $10 a month until I've save enough money; and (3) Sign-up and attend the course.

Creating objectives and activities requires detailed attention and must be taken seriously. Goal-setting is important because it helps the client to:

1. Learn a problem-solving approach that is transferable to other areas of life; and
2. Evaluate progress in very personal and specific terms.

Even if the client does not complete every identified activity, s/he will receive support and feedback allowing the client to learn from the experience. One client from the SUCCESS study described his work with the LC as follows: *"I had a [LC] who had me write every little step down, plan out every day what I was gonna do. I was so used to planning on big things and never seein' 'em get done. It was great to see some progress every day."*

The overall result of the goal-setting process and SUCCESS is to position each client to take responsibility for his/her medical care.

► **Your Role in Developing the SUCCESS Session Plan**: You have multiple responsibilities in developing the SUCCESS Session Plan with each client, and to help his/her accomplish the plan successfully. Help the client to:

- Create SMART (Specific, Measurable, Achievable, Relevant, and Time-bound) objectives.
- Identify activities for each objective.
- Prioritize multiple objectives.
- Identify alternatives to accomplish objectives.
- Weigh the advantages and disadvantages of different actions.
- Connect his/her strengths and assets to the objectives and activities created.
- Become knowledgeable about existing resources to help them achieve their goals.

You or the client should write down the plan. Offer the client a copy of the SUCCESS Session Plan. Make a copy and give it to the client if s/he would like one. While planning could merely be a verbal agreement between you and the client, it is valuable to commit the plan in writing. Doing so provides his/her with a tangible, visual document that identifies his/her goals and the steps necessary to accomplish them. A written plan provides each client with a firm record of his/her accomplishments, and serves as a reminder once the six client sessions are finished.

It is important for you and the client to review the SUCCESS Session Plan during each client session to: (1) assess progress made; and (2) make any necessary adjustments to the plan based on newly identified strengths, goals, or barriers.

General points about the SUCCESS Session Plan are listed below.

- Be attentive to the client's ability to effectively think through a plan, commit to it, and then successfully carry it out. While some clients may be very competent at achieving goals, others may engage in wishful thinking, procrastination, and other thought processes that interfere with moving forward.
- Be precise in helping each client define measurable objectives and the activities necessary to accomplish each objective. The more specific a client is, the more likely s/he is to think through the alternative solutions.
- Maintain professional boundaries. Assume the facilitator role in helping your clients accomplish their objectives and goals.
- Be creative with clients and, when possible, help them to come up with a solution that gets at several barriers at once. The fact that clients frequently have multiple barriers may be overwhelming. Your ability to help them deal with several issues at once will be greatly appreciated.

- Remember to encourage clients to use their strengths as a starting point to accomplish their goals. Periodically summarize strengths you have heard. For instance, if a client has shared that s/he used to deal drugs, you may help the client to see that his/her strengths may be in the areas of talking to people, time management, handling money, and organizational skills. By doing so, the client can use these same strengths to link to care through organizing appointments, seeing the doctor, talking to the pharmacist about medication, and managing money for housing and other expenses.

Check in with clients to ask if they see particular actions, thoughts, or feelings as strengths. Do not impose your view, but assist clients to make those linkages. Ultimately, the client's perception of something as a positive in his/her life will mobilize him/her to solve current problems.

©2017 Center for Health of Incarcerated Persons, Rollins SPH, Emory University

SUCCESS SESSION PLAN

Date: ________________________________

Name: _______________________________

Client ID: ____________________________

Linkage Coordinator: ____________________

Goal 1: Link to Medical Care

Objective 1: ____

Activity		Related Strengths	Potential Barriers	Target Date	Person(s) Responsible
1					
2					
3					

Objective 2: ____

Activity		Related Strengths	Potential Barriers	Target Date	Person(s) Responsible
1					
2					
3					

Goal 2: ______________________________________

©2017 Center for Health of Incarcerated Persons, Rollins SPH, Emory University

Objective 1: ____

**Activity**		**Related Strengths**	**Potential Barriers**	**Target Date**	**Person(s) Responsible**
**1**					
**2**					
**3**					

Objective 2: ____

**Activity**		**Related Strengths**	**Potential Barriers**	**Target Date**	**Person(s) Responsible**
**1**					
**2**					
**3**					

Overview of SUCCESS Document

Instructions: During the first client session, either read the overview verbatim (if your agency has one prepared) or paraphrase its key points. It is important that you confidently convey the key points to the client. Below are the main points that should be conveyed to the client and included in an Overview of SUCCESS document.

- **SUCCESS is time- and session-limited**, up to six sessions over a 90-day period.
- **The goal of SUCCESS is to assist people in linking with medical care soon after receiving a positive test result for HIV**. SUCCESS considers this goal important because people who promptly seek medical care have better health outcomes than those who do not. Promptly linking with medical care can also reduce transmission of HIV to other people.
- **A significant other or important person in the client's life can assist him/her with accessing medical care**. If the client chooses, s/he can bring a significant other or important person to the client sessions and/or medical appointment to assist with the linkage to medical care.
- **SUCCESS can provide practical assistance to the client**, including providing transportation to a clinic, making contact with the clinic, notifying a partner, getting housing, and identifying other barriers to following through with medical care.
- **SUCCESS can help the client identify and overcome barriers to achieve goals – linking to medical care and/or others – by identifying and accessing resources and personal strengths**.
- **The SUCCESS Session Plan will be created to guide the process and track the client's work.** The client can have a copy of any information recorded during these sessions.
- **Sessions can take place at a location, time, and day of the client's choice.**

SAMPLE – Client will be given more updated version

Sample Fact Sheet on Current Treatment Options and their Side Effects

AIDS InfoNet (www.aidsinfonet.org)
A project of the New Mexico AIDS Education and Training Center.
Partially funded by the National Library of Medicine.
Fact Sheet Number 403
WHAT IS ANTIRETROVIRAL THERAPY (ART)

WHAT IS ART? ART means treating retroviral infections like HIV with drugs. The drugs do not kill the virus. However, they slow down the growth of the virus. When the virus is slowed down, so is HIV disease. Antiretroviral drugs are referred to as ARV. ARV therapy is referred to as ART.

WHAT IS THE HIV LIFE CYCLE?
There are several steps in the HIV life cycle. (See Fact Sheet 400 for a diagram.)

1. Free virus circulates in the bloodstream.
2. HIV attaches to a cell.
3. HIV empties its contents into the cell.
4. The HIV genetic code (RNA) is used by the reverse transcriptase enzyme to build HIV DNA.
5. The HIV DNA is inserted into the cell's DNA by the integrase enzyme. This establishes the HIV infection in the cell.
6. When the infected cell reproduces, it activates the HIV DNA, which makes the raw material for new HIV viruses.
7. Packets of material for a new virus come together.
8. The immature virus pushes out of the infected cell in a process called "budding."
9. The immature virus breaks free of the infected cell.
10. The new virus matures: raw materials are cut by the protease enzyme and assembled into a functioning virus.

APPROVED ARV DRUGS Each type, or "class", of ARV drugs attacks HIV in a different way. The first class of anti-HIV drugs was the **nucleoside reverse transcriptase inhibitors** (also called NRTIs or "**nukes**".) These drugs block Step 4, where the HIV genetic material is used to create DNA from RNA. The following drugs in this class are used:

- Zidovudine (Retrovir, AZT)
- Didanosine (Videx, Videx EC, ddI)
- Stavudine (Zerit, d4T)
- Lamivudine (Epivir, 3TC)
- Abacavir (Ziagen, ABC)
- Tenofovir, a nucleotide analog (Viread, TDF)
- Combivir (zidovudine/lamivudine combination)
- Trizivir (zidovudine/lamivudine/abacavir combination)
- Emtricitabine (Emtriva, FTC)
- Truvada (combination of emtricitabine and tenofovir)
- Epzicom (combination of abacavir and lamivudine)

Another class of drugs blocks the same step of the life cycle, but in a different way. These are the **non-nucleoside reverse transcriptase inhibitors**, also called **non-nukes** or **NNRTIs**. Four have been approved:

- Nevirapine (Viramune, NVP)
- Delavirdine (Rescriptor, DLV)
- Efavirenz (Sustiva or Stocrin, EFV)
- Etravirine (Intelence, ETR)

The third class of ARV drugs is the **protease inhibitors or PIs**. These drugs block Step 10, where the raw material for new HIV virus is cut into specific pieces. Ten protease inhibitors are approved:

- Saquinavir (Invirase, SQV)
- Indinavir (Crixivan, IDV)
- Ritonavir (Norvir, RTV)
- Nelfinavir (Viracept, NFV)
- Fosamprenavir (Lexiva, Telzir, FPV)
- Tipranavir (Aptivus, TPV)
- Amprenavir (Agenerase, APV)
- Lopinavir/ritonavir (Kaletra or Aluvia, LPV/RTV)
- Atazanavir (Reyataz, ATZ)
- Darunavir (Prezista, DRV)

A newer class of ARV drugs is **entry inhibitors.** They prevent HIV from entering a cell by blocking Step 2 of the life cycle. Two drugs of this type have been approved:

- Enfuvirtide (Fuzeon, T-20)
- Maraviroc (Selzentry or Celsentri, MVC)

The newest type of ARV drug is the integrase inhibitor. It prevents HIV from inserting its genetic code into the human cell's code in step 5 of the life cycle. The first drug of this type is:

- Raltegravir (Isentress, RGV)

HOW ARE THE DRUGS USED?
Antiretroviral drugs are usually used in combinations of three or more drugs from more than one class. This is called "Combination Therapy." Combination therapy works better than using just one ARV alone, It also helps prevent drug resistance.

Manufacturers of ARVs keep trying to make their drugs easier to take, and have combined some of them into a single pill. See Fact Sheet 409 for more information on combination medications.

WHAT IS DRUG RESISTANCE?
When HIV multiplies, most of the new copies are mutations: they are slightly different from the original virus. Some mutations keep multiplying even when you are taking ARV drugs. When this happens, the drug will stop working. This is called “developing resistance” to the drug.

If only one ARV drug is used, it is easy for the virus to develop resistance. For this reason, using just one ARV drug (monotherapy) is not recommended. But if two drugs are used, a successful mutant would have to “get around” both drugs at the same time. And if three drugs are used, it’s very hard for the right mutations to show up that can resist all three drugs at the same time. Using a triple-drug combination means that it takes much longer for resistance to develop.

CAN THESE DRUGS CURE AIDS?
At present, there is no known cure for HIV infection or AIDS. ARVs reduce the viral load, the amount of HIV in your bloodstream. A blood test measures the viral load. People with lower viral loads stay healthier longer. They are also less likely to transmit HIV infection to others.

Some people’s viral load is so low that it is “undetectable” by the viral load test. This does **not** mean that all the virus is gone, and it does not mean a person is cured of HIV infection. See Fact Sheet 125 for more information on viral load.

WHEN DO I START? There is not a clear answer to this question. Most doctors will consider your CD4 cell count and any symptoms you’ve had. ARV therapy is usually started if your CD4 cell count is dropping to near 350, if you are pregnant, need treatment for hepatitis B, or have symptoms of HIV-related disease. See fact sheet 404 for more information on treatment guidelines. This is an important decision you should discuss with your health care provider.

©2017 Center for Health of Incarcerated Persons, Rollins SPH, Emory University

WHICH DRUGS DO I USE? Each ARV drug can have side effects. Some may be serious. Refer to the fact sheet for each individual drug. Some combinations of drugs are easier to tolerate than others, and some seem to work better than others. Each person is different, and you and your health care provider will have to decide which drugs to use.

The viral load test is used to see if ARV drugs are working. If the viral load does not go down, or if it goes down but comes back up, it might be time to change ARV drugs.

WHAT'S NEXT?
New drugs are being studied in all of the existing classes. Researchers are also trying to develop new types of drugs, such as drugs that will block other steps in the HIV life cycle, and drugs that will strengthen the body's immune defenses. See fact sheets 470 and 480 for more information on newer classes of drugs.

Revised April 24, 2009 *SAMPLE – Client will be given more updated version*

AIDS InfoNet

www.aidsinfonet.org

Fact Sheet Number 550

SIDE EFFECTS

WHAT ARE SIDE EFFECTS?

Side effects are what a drug does to you that you don't want it to do. Medications are prescribed for a specific purpose, such as to control HIV. Anything else the drug does is a side effect.

Some side effects are mild, like a slight headache. Others, like liver damage, can be severe and, in rare cases, fatal. Some go on for just a few days or weeks, but others might continue as long as you take a medication, or even after you stop. Some occur within days or weeks of starting a drug. Others may only show up after months or years of therapy.

WHO GETS SIDE EFFECTS?

Most people taking antiretroviral medications (ARVs) have some side effects. In general, higher amounts of drugs cause more side effects. If you are smaller than average, you might experience more side effects. Also, if your body processes drugs more slowly than normal, you could have higher blood levels and maybe more side effects.

Some side effects become worse if the drug is taken on an empty stomach. Others may increase if the drug is taken with fatty food or drink such as whole milk.

Each medication comes with information on its most common side effects. **Don't assume that you will get every side effect that's listed!** Most people have only minor side effects when they take their ARVs.

HOW TO DEAL WITH SIDE EFFECTS

There are several steps you can take to prepare yourself to deal with side effects. Learn about the normal side effects for the medications you're taking. The InfoNet fact sheets list common side effects for each drug.

- Talk to your health care provider about what side effects to expect. Ask when you should get medical attention because a side effect goes on too long, or has gotten severe.
- Find out if you can treat mild side effects with home remedies or over-the-counter medications.
- In some cases, your health care provider can write you a prescription for something you can take to deal with a side effect if it gets severe.
- Stock up! If you're having stomach problems, make sure you have plenty of food that you like to eat and that's easy on your stomach. Don't run out of toilet paper!

Do not stop taking any of your medications, or skip or reduce your dose, without talking to your health care provider! Doing so can allow the virus to develop resistance (see fact sheet 126), and you might not be able to use some ARVs. ***BEFORE*** **side effects make you skip or reduce doses, talk to your health care provider about changing drugs!**

WHICH SIDE EFFECTS ARE THE MOST COMMON?

When you start antiretroviral therapy (ART), you may get headaches, hypertension, or a general sense of feeling ill. These usually improve and disappear over time.

Fatigue (Fact Sheet 551): People with HIV often feel tired at least part of the time. It's important to find the cause of fatigue and deal with it.
Anemia (Fact Sheet 552) can cause fatigue. Anemia increases your risk of getting sicker with HIV infection. Routine blood tests can detect anemia, and it can be treated.

Digestive Problems: Many drugs can make you feel sick to your stomach. They can cause nausea, vomiting, gas, or diarrhea. Home remedies include:

- Instead of three big meals, eat small amounts, more often.
- Eat mild foods and soups, not spicy.
- Ginger ale or ginger tea might settle your stomach. So can the smell of fresh lemon.
- Exercise regularly.

Don't skip meals or to lose too much weight! Marijuana (see Fact Sheet 731) can reduce nausea. Be careful with over-the-counter or prescription nausea drugs. They can interact with ARVs.

- **Gas and bloating** can be reduced by avoiding foods like beans, some raw vegetables, and vegetable skins.
- **Diarrhea (Fact Sheet 554)** can range from a small hassle to a serious condition. Tell your health care provider if diarrhea goes on too long or if it's serious. Drink lots of liquids.

Lipodystrophy (Fact Sheet 553) includes fat loss in arms, legs and face; fat gain in the stomach or behind the neck; and increases in fats (cholesterol) and sugar (glucose) in the blood. These changes may increase the risk of heart attack or stroke.

High levels of fats and sugar in the blood (Fact Sheet 123), including cholesterol, triglycerides and glucose. This can increase the risk of heart disease (Fact Sheet 652.)

Skin Problems: Some medications cause rashes. Most are temporary, but in rare cases they indicate a serious reaction. Talk to your health care provider if you have a rash. Other skin problems include dry skin or hair loss. Moisturizers help some skin problems.

Neuropathy (Fact Sheet 555) is a painful condition caused by nerve damage. It normally starts in the feet or hands.

Mitochondrial Toxicity (Fact Sheet 556) is damage to structures inside the cells. It might cause neuropathy or kidney damage, and can cause a buildup of lactic acid in the body.

Bone Problems (Fact Sheet 557) have recently been identified in people with HIV. Bones can lose their mineral content and become brittle. A loss of blood supply can cause hip problems. Get enough calcium from food and supplements. Weight-bearing exercise like walking or weight lifting can be helpful.

THE BOTTOM LINE

Most people who take ARVs have some side effects. However, don't assume you will get every side effect you hear about!

Get information on the most common side effects and how to treat them. Read the InfoNet fact sheets on individual drugs and their side effects. Stock up on home remedies and other items that can help you deal with side effects.

Be sure you know when to go back to your health care provider because a side effect may have gone on too long or gotten severe.

Don't let side effects keep you from taking your medications! Do not assume that taking ART means you have to put up with the side effects. If you can't deal with them, if they continue for more than a few months, or they affect your quality of life, talk to your health care provider about changing your drugs.

Revised November 13, 2008

A Project of the New Mexico AIDS Education and Training Center. Partially funded by the National Library of Medicine Fact Sheets can be downloaded from the Internet at http://www.aidsinfonet.org

©2017 Center for Health of Incarcerated Persons, Rollins SPH, Emory University

Appointment Cards

Below are sample appointment cards that you can use to remind your clients of upcoming appointments with you, a medical provider, or other services. At a minimum, the card should include the date, time, and location of the appointment, as well as the agency/provider name.

Appointment

Your appointment is on

____________ at __________a.m/p.m.
(Date) (Time)

____
(Name)

____
(Agency address)

Appointment

Your appointment is on

____________ at __________a.m/p.m.
(Date) (Time)

____
(Name)

____
(Agency address)

Appointment

Your appointment is on

____________ at __________a.m/p.m.
(Date) (Time)

____
(Name)

____
(Agency address)

Appointment

Your appointment is on

____________ at __________a.m/p.m.
(Date) (Time)

____
(Name)

____
(Agency address)

Appointment

Your appointment is on

____________ at __________a.m/p.m.
(Date) (Time)

____
(Name)

____
(Agency address)

Appointment

Your appointment is on

____________ at __________a.m/p.m.
(Date) (Time)

____
(Name)

____
(Agency address)

Session Notes Instructions

The Session Notes form serves as case notes for each client session. You should record all client session information on the form below. One Session Notes form should be completed promptly after meeting with a client and placed in the client's file. You may also find it useful to complete a form following a telephone conversation or when a client cancels or misses an appointment. There are three sections to the Session Notes form: (1) general information about the session; (2) narrative about the session; and (3) the type of referrals made during the session.

Code Listings for the Session Notes form: The following are the suggested codes to use for each of the sections listed on the Session Notes form. These numerical codes will simplify the data entry and data analysis processes, and will allow your agency to input uniform numerical codes rather than words for each field.

Section I

Session Number: Fill in the session number, 1-5. If this is a telephone communication, use numbers starting with "6" for the first phone communication, and then increase the numbering from there. For example, the first phone communication is 6, the second is 7, and so on. If the phone communication is with someone other than the client, e.g., you are contacting a person on the client's Locator Form, do not complete a session form for this communication.

Persons Involved in the Session: Multiple codes may be used to describe who participated in each client session. Note: Not all codes will be used for each session

<u>Section II</u>

Narrative: This section should cover, at a minimum, the following areas:

- Objectives and activities for the session that were or were not accomplished, and why
- Notable client reactions to completing or not completing the objectives and activities
- Client's threats to self or others or pressing medical/psychological problems to be followed-up on immediately Record what parts of the SUCCESS Session Plan were discussed.

<u>Section III</u>

Referrals: This section should include information about all non-medical referrals made during the session. If a referral was made to a community partner(s) (resource, agency, or service provider), enter the code(s) below in the field labeled "Referred to." Note: Please customize the referral categories as your agency sees fit.

Mental Health Treatment – 1	HIV Testing Site -2	Food Pantry – 3
Social Security Admin – 4	State License Bureau – 5	Immigration – 9
Employment – 7	Child/Day Care – 8	Self Help Groups -12
Legal Services – 10	Faith Community – 11	Children's Services -15
Job Center – 13	Vital Statistics Bureau –14	
Clothing/Hygiene – 16	Other – 17(please specify)	
	Housing – 6	

In the "Method of Referral" field, use the following code(s) to record how the referral was handled:

1. LC provided the client with the name and contact information for a referral site(s), and left it up to the client to make the connection.
2. LC called the resource and asked questions on the client's behalf. When appropriate, the LC advocated for the client's involvement with the referral site.
3. LC accompanied the client to the referral site.

Note: Referrals to medical care should not be recorded here.

The code (1, 2, or 3) should be recorded on the "Method of Referral" row under the corresponding "Referral to" column.

Session Notes need not be completed for telephone calls where no new or significant discussions take place. Examples of this might include reminding a client of an appointment or clarifying transportation needs.

Session Notes

Client ID: ___________________ **Date of Session____/_____/_____**

LC Name:
____

Session Start Times:_____:______AM/PM (circle one) **End Time____:_____ /PM (circle one)**

Total Time:_________________ (in minutes)

Session Number (1-6)____________________

Persons Involved in Session: _____, _____, _____, _____, _____ (From Code List above)

Primary Session Location(s): _____, _____, _____, _____, _____ (From Code List above)

Client Transportation to/from Session: _____, _____, _____, _____, _____ (From Code List above)

Narrative:____
____
____
____
____
____
____
____
____

Non-Clinic Referrals

Was a referral(s) made to a community partner (whether another Agency, Resource, or Service Provider) during this Session? Yes (1) or No (2) If yes, where was referral made and how assertive was the referral?

Referred to:						
Method of Referral:						

Linkage Coordinator Signature:____

©2017 Center for Health of Incarcerated Persons, Rollins SPH, Emory University

Session Notes Summary Sheet Instructions

During the Implementation phase, your agency may find it important to summarize non-clinical client information and track a client's overall progress. This Session Notes Summary Sheet is a condensed summary of the all notes recorded on the individual Session Notes forms (Remember: one Session Notes form should be completed per client session).

The information requested in the sheet on the next page can be found in the individual session notes and recorded here. This information can be summarized for the monitoring and evaluation plan (process monitoring) and/or to help to you track clients' progress. The Summary Sheet should be completed **after** the last client session.

Please use the same codes for the Session Notes Summary Sheet as those listed in the instructions for the Session Notes form.

Did the client link to medical care? This question captures information about whether or not a client followed through with the referral to medical care. You may have this information directly because you accompanied the client to the medical appointment. If you did not attend the medical appointment with the client, all efforts should be made to follow up with the client (even if it occurs after the last session). Therefore, this question should be completed based on your personal knowledge, a later personal contact with the client, or from a telephone call from the client or clinic.

Code Listings for the Session Notes Summary form: The code listings for the referrals section of the Session Notes Summary are the same as those suggested codes used on the Session Notes form. These numerical codes will simplify the data entry and data analysis processes, and will allow your agency to input uniform numerical codes rather than words for each field.

©2017 Center for Health of Incarcerated Persons, Rollins SPH, Emory University

Session Notes Summary Sheet

Client ID: _____ _____ ______ Date Summary Sheet Completed _____/______/_____ LC Name: _______________

Contacts	General Information					Referrals		
	Date	Total Time	Persons Involved	Session Location	Transportation (if required, what type was provided)	**Referral Made (Yes or No)?**	**To?**	**Method of Referral**
Session 1								
Session 2								
Session 3								
Session 4								
Session 5								
Session 6								
Phone Contact								
Phone Contact								
Mail Contact								
E-mail Contact								

©2017 Center for Health of Incarcerated Persons, Rollins SPH, Emory University

Total number of scheduled sessions the client missed: No show_______ Canceled_____

Did the client link with a medical care provider? (circle one) Yes (1) No (2) Don't Know (3)

If yes (1), where did the client link?____________________ General Comments (NOT required for data entry):

Case Staffing Form Instructions

The Case Staffing form is designed to encourage you, your Supervisor, and your colleagues to adhere to a strengths approach during the case staffing. Moreover, the form serves as a reminder to always view your clients from a strengths-based perspective, and not only when the client is present.

After each client session, please complete or update the questions below. The *first* question gives you an opportunity to record efforts to meet – both yours and the client's. The *second* question is a brief summary of what encouraged the client to participate in the intervention and follow through with the client session after being diagnosed with HIV. This summary should provide you with insights into the strengths and abilities of the client.

Question 3 asks about the client's strengths. This is not just an exercise to be undertaken. It also serves as a reminder you to view the client from a strengths perspective at all times – regardless if the client is present or not. Furthermore, the discussion will help you understand the client's past or current abilities that will serve the client well in his/her attempt to achieve the goal of linkage to medical care.

Question 4 should include a discussion of the client's barriers – both personal and structural – that s/he sees as interfering with linkage to care. It is important to cast potential deficits or short-comings simply as "barriers" or something that interferes with attaining a goal. The discussion of barriers – rather than problems or pathology – assumes that the client is responsible for and capable of solving them. An emphasis on problems or pathology may create resistance. It is important to check the language you use when speaking to and about your clients – as that may be the source of the problem. This case staffing form is designed to help you get to the source of the problem and assist the client to achieve his/her goal of linkage to care, among others.

Case Staffing Form

Client ID:____
Referred to SUCCESS by:____
Data Assigned to SUCCUSS (month/date/year):____
LC Name:____
LC referred client to:____
Method of Referral:____
Date Referred (month/date/year):____

1. **Describe your early attempts to make contact with the client:**
   ____
   ____
   ____
   ____
   ____

2. **Why did the client decide to participate in SUCCESS?**
   ____
   ____
   ____
   ____
   ____

3. **Please describe at least three (3) of client's most significant strengths:**
   ____
   ____
   ____
   ____
   ____

4. **Describe the client's barriers to medical care linkage (individual- and system-level)**:
____
____
____
____
____

5. **What are the client's goals in addition to linking with medical care?**
____
____
____
____
____

6. **Did the client link to medical care or express a desire to link to medical care?**
____
____
____
____
____

©2017 Center for Health of Incarcerated Persons, Rollins SPH, Emory University

Life Domains List

Introduction: The Linkage Coordinator can use the Life Domains List questions as a refresher about various life domains and ways that clients show strengths in those domains. Use these questions only as a reference or stimulus to help the client when s/he may be having difficulties identifying strengths. You do not want the exercise to become a catalog of past failures. Please note: Using while the client is present, and writing down the "answers" to the life domain list is not appropriate.

General Life Skills

Do you:

- Cook meals for yourself and/or others?
- Help others cook meals?
- Shop for groceries or other necessities?
- Ride public transportation?
- Wash your own clothes?
- Keep up-to-date on current events?
- Arrive to your appointments on time?
- Seek out information using the Internet, phone book, or other resources?
- Read the newspaper?
- Take care of others – maybe your kids, parents?

Relationships

Do you:

- Trust others easily?
- Have relationships with other people (either sexual or non-sexual)?
- Have realistic expectations of relationships?
- Resolve conflicts assertively?
- Have a good relationship with family members?
- Have positive relationships with friends?
- Seek out community groups?
- Have a spouse or significant other?
- Have flexibility in your interactions with others?
- Function independently?
- Generally respect other people?

Living Arrangements

Do you:

- Live by yourself and take of the place (apartment, house) on your own?
- Clean and/or provide maintenance on your place?
- Feel as if your living arrangement supports your overall well-being?
- Take pride in your home/apartment

Health

Do you:

- Generally get enough sleep?
- Exercise regularly? If so, what do you like to do?
- Go for regular medical/dental check-ups? Have you in the past?
- Generally address any health problems as they arise?
- Generally feel comfortable asking questions of your doctor or other health providers?
- Generally take your medicine on time and as prescribed?
- Practice safe sex with your partner(s)?
- Maintain a healthy diet?

What are some things you do to reduce/manage stress in your life?

Have you attempted to change an unhealthy behavior before? If yes, what was it? How did you feel about it?

Internal Resources

Do you:

- Often set goals for yourself?
- Understand how your behavior affects you and others?
- Verbalize your wishes and desires directly?
- Value your strengths and talents?
- Consider the consequences of your actions/behaviors before acting?
- Follow your beliefs and values?
- Value/acknowledge your accomplishments?
- Attend to your spiritual needs (either through church, house of worship, others)?
- Seek help as needed for personal problems?
- Articulate your interests?
- Think you have good decision-making skills?
- Accept responsibility for your actions?
- Express your emotions regularly and appropriately?
- Feel in control of your life?
- Effectively delay gratification or seek instant gratification?
- Generally cope with uncomfortable emotions in a positive way?

For active or past drug users:

Recovery

Have you:

- Sought drug treatment?
- Explored your past/current drug use during treatment?
- Avoided people/places where drug use was prevalent?
- Followed through with aftercare?
- Maintained sobriety in the past?
- Attempted to change drug use behavior in the past?
- Attended support groups?

©2017 Center for Health of Incarcerated Persons, Rollins SPH, Emory University

- Found a sponsor?
- Maintained contact with your sponsor?

SUCCESS Client Satisfaction Questionnaire

Linkage Coordinator's Name: ____
Your Name or Client ID (optional): ____
Please circle the best answer to the questions below:

1. How satisfied are you with your experience participating in the SUCCESS intervention?

Very Satisfied **Satisfied** **Neutral** **Dissatisfied** **Very Dissatisfied**

2. How satisfied are you with the services, if any, you were linked to during SUCCESS?

Very Satisfied **Satisfied** **Neutral** **Dissatisfied** **Very Dissatisfied**

3. How satisfied are you with the skills you learned and/or enhanced by participating in the intervention?

Very Satisfied **Satisfied** **Neutral** **Dissatisfied** **Very Dissatisfied**

4. How satisfied are you with the Linkage Coordinator you worked with over the course of the intervention?

Very Satisfied **Satisfied** **Neutral** **Dissatisfied** **Very Dissatisfied**

Please write in your answers to the questions below.

5. What did you *like most* about participating in the intervention?

6. What would you *change* about the intervention?

7. Would you recommend SUCCESS to anyone you know?
Yes **No** **Please explain why or why not?** ______________

Thank you for your feedback

Client Session Guide Helpful Tips

As a supplement to the Client Session Guide, the following is an overview of additional helpful tips to consider when implementing SUCCESS.

1. **Attending a Medical Appointment with the Client**
   If the client agrees, plan to attend the first medical appointment with him/her. The client should take the lead in the conversation with the health care provider and advocate for himself/herself during the appointment. It is not your role to control the conversation during appointment. Unfortunately, there is no prescribed recipe to balance client involvement with you advocating on his/her behalf. However, two extreme scenarios to be avoided under all circumstances are:
   1. You and the medical staff talk about a client, his/her circumstances, or treatment as if the client was not present.
   2. You allow the client to struggle significantly while dealing with the provider and let critical issues go unaddressed.

While the goal of SUCCESS is to link recently diagnosed individuals to medical care, the desired outcome is for the medical appointment to be successful and for the client to feel comfortable and empowered to continue medical services. The client will judge your status as an effective and trustworthy ally based on the success or failure of the first medical appointment. To be seen as a trustworthy ally, prepare the client to increase the likelihood of a successful outcome.

2. **Preparing the Client for the Medical Appointment** Inform the client in advance about what to expect during the visit. This information should be discussed in detail during the client session prior to the medical appointment. You and the client can write down any questions the client would like to discuss during his/her appointment. Writing questions down in advance will ensure the client does not get anxious and forget to ask an important question.

   Provide the client with detailed information about the clinic/agency and staff. Much of this information will be available on the appointment card and includes, but is not limited to, the clinic name, doctor’s name, address, hours, phone number, and appointment time. Additional information to be provided to the client includes directions to and from the clinic, transportation options provided by your agency, a reminder of where and when to meet, the personalities of various staff, services provided, anticipated wait time, and any other relevant information.

   If you accompany the client, the client should know that s/he is expected to participate fully in the medical appointment and represent himself/herself. You are there for support and will only interject if the client asks for assistance and/or appears to need your assistance.

Tips for a Successful First Medical Appointment:

► Prior to the medical appointment, provide the client with detailed information about the provider s/he selected.

► Help the client prepare a set of questions, such as:
- How often should I come visit you?
- If I feel fine, do I still have to take my HIV medications?

► Discuss potential problems (such as the client forgetting what medications s/he is taking) that may occur during the appointment and brainstorm possible solutions with the client.

► Introduce the client to clinic staff, including screening staff, nurses, pharmacists, physicians, and social workers.

► Participate in all meetings between the client and clinic staff, if requested by the client.

► Explain the rationale behind different processes (administrative) during the appointment to the client.

► Interpret questions and/or information being given to client, as necessary. If necessary, ask clarifying questions of clinic staff.

► After the appointment, review with the client what happened during the visit and how the client felt.

► Schedule a brief follow-up telephone call with the client to process the visit.

► Schedule an in-person session with the client to complete the transition process.

In conclusion, the client must attend the first medical appointment *thoroughly briefed* by you and *empowered to act on his/her own behalf*. During the appointment, you must continually assess the degree to which you should facilitate the client's involvement or act assertively on the client's behalf.

3. **Structure of Client Session**
   You will have to adjust the time of each session to the individual SUCCESS client. Due to the intensive, short-term nature of the intervention and the variation in time needed for each client session, you should schedule only two to three clients in a day until you become more familiar with the client and his/her needs. Because a client session may take a few hours, you may want to schedule one client in the morning and one client in the afternoon to allow sufficient time for the session. During the SUCCESS-II study, the median number of sessions conducted per client was two sessions. The median time spent on all activities per client was 5.8 hours (the mean was 7.2 hours) and the range was from 0 to 36.7 hours per client.

   Your caseload should be kept low (25-30 clients at a given time) to accommodate for long sessions with each client and for extensive follow-up after missed appointments. If the client is late to a session, use it as an opportunity to identify a barrier to effectively linking with medical care as opposed to seeing it as a client weakness. Part of your work with the client can be to help him/her identify the source of his/her lateness and plan to solve the problem.

4. **Meeting Space** While meeting each client in his/her environment and outside the office whenever possible is a Core Element of the intervention, your agency may want to create and train you on general safety guidelines. If you are uncomfortable with a meeting place or

meeting with a client at night, you may bring a colleague (if approved by the client) or change the meeting time or location while still ensuring the client is comfortable. Suggested locations include: your agency's office, a clinic or hospital, a public library, restaurant, community partner's office or clinic.

5. **Telephone Contacts with the Client** Telephone communications between you and client are intended to be used in the following ways:
   ► Initiate the SUCCESS intake process, e.g., to discuss the intervention with the client and determine if s/he is interested and eligible to participate.
   ► Reinforce, review, or modify logistics for an upcoming client session, e.g., the meeting time.
   ► Identify any barriers that arose since the last client session and may impact the client's ability to attend the next session, e.g., child care is now a barrier.
   ► Touch base between client sessions, if it is an extended amount of time or if you feel the client needs a little encourage or a reminder of the next client session.

Telephone communications are **<u>not</u>** intended to:

► Replace in-person client sessions
► Be lengthy
► Be used to identify strengths

If telephone communications between you and the client cover any of the items above, approval by the Program Director/Manager or Supervisor is required in advance.

6. **Approaches to Completing Paperwork** Completing required paperwork for SUCCESS and for your agency's regular administrative and enrollment processes places an additional burden on your time. Moreover, your agency's required paperwork may focus on *inabilities* and, therefore, not adhere to the Core Elements. If your agency's clinical forms are not adjusted in the Pre-Implementation phase to make them more strengths-focused, then the SUCCESS Program Director/Manager will have to negotiate possible solutions with the Clinical Director or Executive Director to ensure all required paperwork is completed. In the case of SUCCESS paperwork, the strengths perspective must be maintained.

   Below are two different options available to complete paperwork with the client. Whenever possible, your agency should try to make the paperwork for SUCCESS and other programs as complementary as possible. Choose whichever option best fits your style and/or your agency's needs. The options are:

   Option 1: Clearly differentiate the agency-required documents from the SUCCESS-required documents and complete the agency-required documents first. Make it a point to differentiate the SUCCESS paperwork from the agency-required paperwork during the first client session. By doing so, you clear up any confusion the client may have if the agency-required documents focus on deficiencies or inabilities.
   When the agency-required paperwork is finished, tell the client you will now move in a new direction. Set aside the paperwork, and continue with the activities listed in Session One.

OR

Option 2: Emphasize the SUCCESS-required documents by addressing them first. Begin the session by very quickly addressing any paperwork that is absolutely essential to complete first, such as consent forms. Continue with the activities listed in Session One. Whenever it feels appropriate, tell the client about the agency-required documents. Since these documents may highlight the client's deficiencies or inabilities, it is important to:

- ► Not overemphasize the client's deficiencies, inabilities, or weaknesses
- ► Maintain good eye contact
- ► Practice effective communication skills
- ► End the session with a summary of the client's strengths

Other than the agency-required documents and forms completed together, complete paperwork after your session with the client.

7. **Significant Others** Explore the role of significant others (partners, family, friends, or someone important in the client's life) in either promoting or interfering with a client's linkage to medical care, and be prepared to discuss this issue with client. Significant others can influence a client following through with his/her medical appointment in many ways. In some instances, significant others can assist with linkage; in others, their involvement could interfere with linkage and follow-through.

8. **Providing Incentives for Clients** Follow your agency's policies when deciding whether to provide incentives for clients or create a SUCCESS-specific policy around this issue. Incentives can be helpful in facilitating client involvement and connecting with him/her. Incentives are a great way to retain clients and keep them involved in the process. Your agency may want to consider creative strategies to get incentives donated. A few examples include: (1) asking a local grocery store to donate gift cards for food purchases; (2) asking the local gas station to donate gas cards; (3) asking a local restaurant to donate gift cards (this could be especially useful if you and the client meet for several hours and it is time for a meal); or (4) asking a phone company to donate calling cards. If the agency does not allow you to transport clients, it may be useful to provide them with transportation money or vouchers to enable clients to travel to each client session and medical appointment.

9. **Finding Clients Who Drop Out** Once you make initial contact with the client, the relationship will last for six sessions or 90 days, whichever comes first. If a client does not follow through with SUCCESS or is unable to be reached, you should attempt to contact the client at least through the 90-day period. After this time, you should decide how and whether to pursue the client on a case-by-case basis. A client who has clearly indicated that s/he does not wish to be contacted should be asked why s/he is dropping out but not pursued further. This section provided in-depth information on the activities and skills that help facilitate effective implementation of SUCCESS.
