# Supplementary material for: Jail, an unappreciated medical home: Assessing the feasibility of a strengths-based case management intervention to improve the care retention of HIV-infected persons once released from jail
Source: PLoS One. 2018 Mar 30;13(3):e0191643. doi: 10.1371/journal.pone.0191643 (PMC5877830; doi:10.1371/journal.pone.0191643)
Supplement: S4 File — (ZIP) [file pone.0191643.s004.zip › SUCCESS_Protocols_IRBApprovalLetters/SUCCESS Protocol Approval Letter 2014.pdf]

EMORY UNIVERSITY | Institutional Review Board

TO: Anne Spaulding, MD
Principal Investigator
Epidemiology

DATE: April 1, 2014

RE: **Continuing Review Expedited Approval**
CR1_IRB00064852

IRB00064852
Planning for Success--The next step of a project to improve the connection to community care for HIV infected persons leaving Fulton County Jail in Atlanta: Phase 1 and Phase 2

Thank you for submitting a renewal application for this protocol. The Emory IRB reviewed it by the expedited process on 03/31/14, per 45 CFR 46.110, the Federal Register expeditable categories F(5), F(7), and/or 21 CFR 56.110. This reapproval is effective from **03/31/14** through **03/30/15**. Thereafter, continuation of human subjects research activities requires the submission of another renewal application, which must be reviewed and approved by the IRB prior to the expiration date noted above. Please note carefully the following items with respect to this reapproval:

- Subpart C 46.306(a)(2), namely (iv): This study has been approved to include prisoners as subjects.
- Partial HIPAA waiver renewed: This study meets the criteria for HIPAA Authorization to be waived for the purposes of determining eligibility or recruiting subjects for this study. As subjects are enrolled, you are required to obtain authorization.

Documents reviewed with this application:

- supplement_protocol_for_ IRB4_06102013_clean.part1
- IRBprotocol_Phase2_r34_92.part2
- Blood_draw_consent_93_CC_Comments_track-changes_09202013.doc_1042013
- CONSENT_success_Phase2_CC_change_comment_09202013_revised 104
- Supplement_Consent-Authorization-Intervention_061013

Any reportable events (e.g., unanticipated problems involving risk to subjects or others, noncompliance, breaches of confidentiality, HIPAA violations, protocol deviations) must be reported to the IRB according to our Policies & Procedures at www.irb.emory.edu, immediately, promptly, or periodically. Be sure to check the reporting guidance and contact us if you have questions. Terms and conditions of sponsors, if any, also apply to reporting.

Before implementing any change to this protocol (including but not limited to sample size, informed consent, and study design), you must submit an amendment request and secure IRB approval.

In future correspondence about this matter, please refer to the IRB file ID, name of the Principal Investigator, and study title. Thank you.

Sincerely,

Scott S. Katz, MS
Research Protocol Analyst
This letter has been digitally signed

CC:	Bowden	Chava	Epidemiology
	Del Rio	Carlos	Global Health
	Frew	Paula	MedInfect
	Johnson	Brent	Biostatistics

Emory University
1599 Clifton Road, 5th Floor - Atlanta, Georgia 30322
Tel: 404.712.0720 - Fax: 404.727.1358 - Email: irb@emory.edu - Web: http://www.irb.emory.edu/
An equal opportunity, affirmative action university
