# Supplementary material for: Jail, an unappreciated medical home: Assessing the feasibility of a strengths-based case management intervention to improve the care retention of HIV-infected persons once released from jail
Source: PLoS One. 2018 Mar 30;13(3):e0191643. doi: 10.1371/journal.pone.0191643 (PMC5877830; doi:10.1371/journal.pone.0191643)
Supplement: S4 File — (ZIP) [file pone.0191643.s004.zip › SUCCESS_Protocols_IRBApprovalLetters/SUCCESS_IRBProtocol_June2013.docx]

**Preliminary Planning for Success--The next step in a project to improve the connection to community care for HIV infected persons leaving Fulton County Jail in Atlanta.**

Principal Investigator’s Name:

Anne Spaulding MD MPH

Assistant Professor, Department of Epidemiology

Rollins School of Public Health, Emory University

1518 Clifton Road NE, Room 3033

Atlanta, GA 30322

aspauld@emory.edu

(404) 727-3369

Co-Investigators: Carlos del Rio MD

Paula Frew PhD

Sponsor: National Institute of Drug Abuse (Award Pending as of Submission)

Date: June 10, 2013

Abstract:

The need to have evidence-based interventions to keep HIV infected persons linked to care as they traverse the maze of the criminal justice system is great. We are refining an intervention that we developed to promote connection to community HIV care at the time of jail discharge. Our ultimate goal is to test this intervention using a randomized clinical trial. Prior to testing the intervention, we need to demonstrate the ability to recruit persons to a linkage intervention at a pace that would support an adequately powered trial. In 12/2012, a pharmaceutical company awarded funding to sustain for 12 months an opt-out, universal HIV testing program that we established at Fulton County (GA) Jail, the proposed site of this pilot project. If a supplement can be put in place this spring, it will allow us to demonstrate that we can recruit and provide linkage services concurrent with the testing program. Having linkage services available is crucial to the success of the testing demonstration project. Success with the testing program this year will ensure that the company will underwrite continued testing in the future, which will benefit our future studies of the linkage intervention.

**1. Background**

**a. Specific aims of the study**

A cohort study recently showed that if persons living with HIV entered a jail, their odds of having their HIV medications disrupted increased 8-fold over baseline.[^1^](#_ENREF_1) Each year, 1 in 7 persons with HIV in the U.S. enters a jail.[^2^](#_ENREF_2) Public health interventions to improve engagement in care for HIV+ persons leaving jail are rare. Most interventions to help incarcerated HIV+ individuals have focused on those in prison; 95% of all U.S. inmates, however, pass only through jails.[^2^](#_ENREF_2) Releasees of jails in Atlanta, GA, where this project will be based, navigate very complicated individual and systems-level barriers to staying in HIV care: one-third of these individuals are homeless or unstably housed, and most suffer from alcohol and/or drug dependence.[^3^](#_ENREF_3) When translated to the nation as a whole, potentially ~150,000 HIV+ releasees per year face similar obstacles to HIV care.[^2^](#_ENREF_2)^,^[^4^](#_ENREF_4)^,^[^5^](#_ENREF_5)

In response, we propose a one-armed feasibility study to develop data demonstrating that jail releasees will be willing to engage in intensive case management beginning in jail and that case management can promote the establishment in care of HIV+ jail releasees in the community. To address the needs of this population, we will implement an adaptation of a CDC-disseminated model[^6-8^](#_ENREF_6) of Strengths-Based Case Management developed in non-correctional settings. Randomized controlled trials have shown that Strengths-Based Case Management promotes lasting linkage of HIV+ persons to community-based HIV care, including access to long-term antiretroviral treatment.[^7^](#_ENREF_7)^,^[^8^](#_ENREF_8) Our adaptation of Strengths-Based Case Management for jails features wrap-around case management (i.e., spanning before and after jail discharge) to promote linkage in HIV healthcare, substance abuse treatment, and mental health services.[^2^](#_ENREF_2)^,^[^9^](#_ENREF_9)In this short feasibility project, we will demonstrate the ability to recruit and link individuals in care, rather than rigorously test the evaluation tools or the intervention itself.

This proposed project, ***Preliminary Plans for SUCCESS*** (**Sustained, Unbroken Connections to Care, Entry Services, and Suppression)**, will test the logistics and acceptance of the intervention. For the present study, we aim to explore if **SUCCESS** recipients will link to care at rates that match nationwide norms for the non-incarcerated. Findings from this feasibility study of **SUCCESS**, if successful, will address a gap in the literature by informing the planning of a novel randomized clinical trial to examine long-term effectiveness and cost of intensive case management, as compared with usual care for the hard-to-treat population of jail detainees.

**Aim 1. Connection to care:** Recruit HIV+ jail detainees at a pace of 14 per month for 3 months, deliver Strengths-Based Case Management in jail and during the first 60 days in the community (reinforced with cell phone contact upon release), and demonstrate that enrolled releasees will link to HIV care by 3 months post release. A successful linkage to HIV medical care will be defined as a confirmed visit to a clinic post release, validated by a recorded HIV viral load and CD4 count in the clinic’s medical records.

*Hypothesis: 75% of* ***SUCCESS*** *participants will link to HIV medical care by 3 months post-release.*

**Aim 2. Sustain a testing and linkage program:** Having linkage services available is crucial to the success of the testing demonstration project. Success with the testing program this year will ensure that the company will underwrite continued testing in the future, which we will benefit our future studies of the linkage intervention. The goal for next year is to conduct a full scale feasibility study that is dependent on continued industry support of testing. The ultimate goal is to prepare a protocol for testing SUCCESS in a randomized controlled trial and to conduct that study.

**b. Preliminary studies in area that support this stage of the research and significance/justification for current study**

**i. Improving linkage and retention in HIV care is urgently needed.** Depicting engagement in care as a cascade, a paper by Gardner, del Rio and colleagues showed that persons with HIV disease are lost at each step along a continuum, from diagnosis to linkage and retention in care to suppression of viral load.[^10^](#_ENREF_10) Among persons in the U.S. diagnosed with HIV, 77% are linked to care, 51% are retained in care, and a mere 35% are virally suppressed.[^11^](#_ENREF_11) Furthermore, men, Blacks, and drug users are less likely to have regular medical care (as measured by obtaining ≥ 2 CD4 or viral load tests ≥ 3 months apart) compared respectively to women, Whites, and those in other HIV risk categories, according to the National HIV Surveillance System.[^12^](#_ENREF_12) These groups with poorer retention in care are the very groups over-represented in the U.S. criminal justice system.[^13^](#_ENREF_13) Concurrent with the HIV epidemic, the U.S. is experiencing an epidemic of hyper-imprisonment,[^14^](#_ENREF_14) where incarceration rates have surpassed comparable rates of all other countries.[^15^](#_ENREF_15) Dr. Spaulding, the PI of this proposal, recently showed that although the share of the HIV epidemic in the U.S. borne by persons released from a correctional facility in a calendar year has dropped from 19.8% in 1997 to 14.0% in 2006, the absolute number of HIV+ persons passing through jails and prisons has remained unchanged since 1997 due to these escalating incarceration rates.[^2^](#_ENREF_2) Collaboration between public health and corrections is vital to efforts to control HIV domestically.

Management of HIV in jail settings is understudied.[**^9^**](#_ENREF_9) Ninety-five percent of the 150,000 HIV+ persons released annually from U.S. correctional facilities leave jails, which are short-term, high throughput correctional facilities usually run by counties or cities for those who are awaiting trial or serving brief sentences.[^2^](#_ENREF_2) However, most interventions have dealt with prison populations. Rapid population turnover differentiates jails from prisons, suggesting that a fast-paced, flexible intervention would be needed in a jail setting. A reproducible intervention for increasing linkage to and retention in HIV care for jail releasees has not yet been established. Despite the wide use of case management in HIV care,[^16^](#_ENREF_16) a published randomized control trial (RCT) demonstrating its efficacy in jails is lacking; an expert panel has recently called for evaluation of case management in HIV care, specifically strategies for assisting the incarcerated upon release.[^17^](#_ENREF_17)

The ultimate goal of our research is to conduct a trial of Strengths-Based Case Management (SBCM) for jail releasees versus usual care, coupled with a cost analysis. Most jails do not provide linkage services for HIV+ releasees; our nation is in a state of uncertainty where jurisdictions running jails are not convinced that providing such services merits the cost. However, if we showed the SBCM intervention to be efficacious, scalable, and cost effective, then jails could provide a highly structured environment where the intervention might be delivered routinely to HIV+ persons. Our feasibility study will implement the protocol and gather preliminary data necessary for a power analysis before conducting an RCT and cost analysis. We seek to contribute to the development of an evidence base for interventions to optimize the number of persons at the “fully engaged/retained” end of the HIV care spectrum among those leaving jails.[^10^](#_ENREF_10)^,^[^18-20^](#_ENREF_18)

**ii. Evidence-based methods for linkage and retaining HIV+ persons to care are needed for high-risk jail releasees*.*** Access to HIV medical care in correctional facilities can be tenuous, especially for the short-term jail detainee.[**^21^**](#_ENREF_21) The number of HIV+ persons attaining viral suppression while incarcerated varies by institution and length of stay (LOS). While suppression can be high during incarceration, as a function of the efficacy of antiretroviral therapy (ART), releasees may cease to seek care for their medical conditions upon leaving a correctional facility.[^22^](#_ENREF_22)^,^[^23^](#_ENREF_23) Disengagement can be due to real or perceived financial and logistical barriers,^39^ misunderstanding of the need for care, competing needs,[^24^](#_ENREF_24) mistrust of community providers,[^25^](#_ENREF_25) and sometimes, inhospitable outside healthcare systems.[^25^](#_ENREF_25) For those on ART, a short incarceration is more disruptive than a prison sentence.[^1^](#_ENREF_1) A San Francisco study showed that 76% of 512 HIV+ jail detainees with multiple incarcerations had interruptions in their ART upon release.[^26^](#_ENREF_26) Jail stays are often long enough to interrupt scheduled appointments with usual community providers, but too short to apply discharge planning strategies that have been developed for prisons. For example, the North Carolina Prison System implemented a program of intensive HIV discharge planning, where nurses met with HIV+ inmate-patients repeatedly over their last 3-6 months of imprisonment to develop a plan for post-release medical care.[^27^](#_ENREF_27) This strategy would not work in jails, where the mean LOS is just 2 to 3 weeks. Furthermore, treatment gaps pose a danger to uninfected sex and drug use partners as well; many releasees may be prone to behavior associated with HIV transmission after being released, such as substance abuse and resultant unprotected sex.[^28^](#_ENREF_28)^,^[^29^](#_ENREF_29) Studies have shown that effective treatment with ART can reduce the likelihood of HIV transmission to seronegative sexual partners.[^30-32^](#_ENREF_30) For all of these reasons, current efforts to expand HIV testing in jails must be coordinated with efforts to link and retain persons in community care post discharge.[^33-35^](#_ENREF_33) Retention among jail releasees that is better than the 24% seen in San Francisco can help the U.S. achieve the aim of the National HIV/AIDS Strategy on retention: ***Increase the proportion of Ryan White HIV/AIDS Program clients who are in continuous care (≥2 visits for routine HIV medical care in 12 months ≥ 3 months apart).***[***^36^***](#_ENREF_36)

**iii. Models for improving post-release healthcare linkages.** Para-professional “peer” navigators have been used to help promote linkage of HIV+ persons to care in many settings.[^17^](#_ENREF_17) However, peers of inmates often have criminal records themselves; without passing a criminal background check, these peers cannot work in the criminal justice setting. As a result, an intervention using professional case managers (CMs) is more appropriate for a jail-based study. Strengths-Based Model: Case management for HIV+ persons[^37^](#_ENREF_37) can employ different models. The two most common are the Broker model, where a CM assertively guides care, and the Strengths model, where a CM draws upon the strengths of the client.[^38^](#_ENREF_38)^,^[^39^](#_ENREF_39) The language of mental health rehabilitation, social work, and counseling (including offender rehabilitation) has incorporated the “Strengths-Based” model of concentrating on human potential rather than deficits by “looking at a glass as half-full rather than half-empty.”[^6^](#_ENREF_6)^,^[^38-40^](#_ENREF_38) Incorporating the concept of self-efficacy from Social Cognitive Theory,[^41^](#_ENREF_41) the SBCM approach includes three principles: (i) establishing a trusting relationship with the CM, (ii) drawing on any past successes and a sense of self-efficacy in addressing current challenges (or barriers), and (iii) enhancing the participant’s sense of control in critical areas of their life. SBCM has consistently demonstrated its value in substance abuse treatment,[^42^](#_ENREF_42)^,^[^43^](#_ENREF_43) specifically in linking cocaine users with treatment[^44^](#_ENREF_44) and retaining them in aftercare.[^45^](#_ENREF_45)^,^[^46^](#_ENREF_46) SBCM has become one of the dominant models in HIV medical case management.[^37^](#_ENREF_37) SBCM without formative research was used in a 2-arm comparison trial among HIV+ North Carolina state prisoners: SBCM versus Broker-style, nurse-led discharge planning. This study showed statistically equivalent linkage between the two arms (65% and 54% respectively).[^27^](#_ENREF_27) Outcomes in both arms exceeded the anecdotally-reported, historical rate of 20% linkage (personal communication, D. Wohl).

**iv. Efficacy of SBCM in HIV care—the *ARTAS* study.** SBCM was the basis for *ARTAS* (Antiretroviral Treatment Access Study), a 4 city CDC-funded study which randomized recently diagnosed non-incarcerated HIV+ subjects to brief SBCM and care planning versus usual care. Dr. del Rio (co-investigator of this study) was PI for *ARTAS*’ Atlanta site. The outcome of interest was self-reported attendance (confirmed by medical record abstraction) at an HIV clinic twice in a 12-month period, an endpoint chosen because it was determined to be the minimum frequency of care required for any HIV+ person, even at early stages. Participants in the intervention arm received up to 5 sessions with a professional CM to facilitate linkage to HIV care. An important component of case management in *ARTAS* was that CMs and the client began by devising a plan to overcome barriers to HIV treatment, which included filling out paperwork for benefits and planning appointments for ancillary support services. Participants in the usual care arm received information about HIV and local care resources, along with a referral to a provider. Follow-up data were available on 91% of participants at 6 months and 86% at 12 months. Those who received the intervention were significantly more likely than controls to have had at least 1 HIV primary care visit in each of 2 consecutive 6-month follow-up periods. At 6 months follow up, 78% of participants receiving the intervention had linked to care, compared to 60% among controls. At 12 months follow up, 64% of subjects in the intervention arm had linked to *and were retained* in care compared to 49% in the control arm (RR(adj.): 1.41; p=0.006).[^7^](#_ENREF_7)

**v. Reproducibility of *ARTAS.*** An implementation study, *ARTAS*-2, followed the original *ARTAS* study and showed similarly high rates of linkage (79%).[^8^](#_ENREF_8) Dr. del Rio recently adapted *ARTAS* for hospitalized HIV+ users of crack cocaine: Hospital Visits as Opportunity for Prevention and Engagement (*HOPE*).[^47^](#_ENREF_47)^,^[^48^](#_ENREF_48) The CDC has selected *ARTAS* for national diffusion in the Diffusion of Effective Behavioral Interventions (DEBI) program; a procedures manual is available.[^49^](#_ENREF_49) Between April and July 2012, we adapted this manual to produce a 102-page Client Services Guide specifically for **SUCCESS**.

**vi.. Preliminary data. *EnhanceLink*.** Dr. Spaulding has demonstrated that HIV testing in jails represents an opportunity to find previously undiagnosed persons early in their disease course and link them to care, and that a jail intervention can re-establish linkage for those who have fallen out of care**.**[^50-52^](#_ENREF_50) *EnhanceLink*, a 10-site, 6-year, $21 million demonstration project funded by HRSA, evaluated the feasibility and outcomes of non-standardized models of HIV testing and linkage in jails.[^53^](#_ENREF_53) Many on our current study team previously worked together successfully on *EnhanceLink*: Dr. Spaulding served as PI of the *EnhanceLink* Evaluation and Support Center and Dr. Frew (co-investigator on this present study) served as the Center co-investigator. Dr. Rapp (consultant on this study) served as evaluator for the Columbia, SC site and Ms. Bowden (project coordinator on this study) served as a case manager for the Atlanta site. The Center collaborated with grantees to design common, project-wide instruments for evaluation based on validated instruments and operated a web-based data management system into which sites entered data. The Center took the lead in interim and final analyses of the data. In collaboration with Center and site investigators, Dr. Spaulding authored a dozen papers reporting study findings,[^50^](#_ENREF_50)^,^[^52-62^](#_ENREF_52) many of which will be published in an upcoming supplement to *AIDS and Behavior*, which the Center organized for the *EnhanceLink* initiative*.*

In *EnhanceLink***,** no standardization of interventions was attempted; HRSA allowed grantees to design any model of service to enhance linkage to care, and the study did not have a control arm. No site developed a manualized intervention as specific as the **SUCCESS** Client Services Guide prepared for this application. Nonetheless, of 9,837 HIV+ detainees offered various linkage services, 8,056 (82%) accepted, although 10% later disenrolled because they were transferred to prison. Among releasees, 59% made at least 1 visit to an HIV provider in the community (linkage); 53% had 2 or more visits to community HIV services by 6 months (retention); and 26% demonstrated viral suppression at 6 months, even when persons with missing data were considered unsuppressed.[^55^](#_ENREF_55)^,^[^57^](#_ENREF_57) Having a CM review the need for medical care post discharge was highly predictive of an undetectable viral load at 6 months.[^55^](#_ENREF_55) We anticipate that with a standardized intervention of using SBCM in **SUCCESS**, rates of linkage and retention will be 75% and 50%, respectively, i.e., essentially the national norm. While these rates are less ambitious than rates achieved in ARTAS because of the multitude of challenges that releasees face, they would exceed those of *EnhanceLink,* since the intervention would be systematically applied.

**Local jail data.** In 2010, Dr. Spaulding began a CDC-funded study, Integrating Infectious Disease Detection at Entry and Linkage to care (*IIDDEAL*). *IIDDEAL* expanded HIV testing at Fulton County Jail (one of the five largest jails in metro Atlanta and the site for this proposed project—see letter of support). The focus of this study was testing and linkage to care within the jail. HIV prevalence at the jail is between 2.2 and 3.2%.[^63^](#_ENREF_63) Our group trained jail nurses in rapid testing by mucosal swab using an opt-out strategy (default action is to test, unless participant objects). The working relationships developed with the nursing staff at Fulton County Jail will facilitate ***Preliminary Planning for SUCCESS***. The turnover of the population at the jail during the study period was rapid: the median LOS for all persons booked was 5 days and the mean was 22 days (see Figure 1A). Nonetheless, our team was able to successfully implement HIV testing. The mean number of persons booked per night was 114 (range 18-220). One in six persons released returned within the year (16.1%; see Figure 1.B.), which indicates that a jail intervention needs to account for people cycling in and out of jail in the follow-up period. Between December 2010 and March 2012, 21,486 offers of HIV testing were made to entrants of the jail, 13,827 offers (64%) were accepted, and 54 people (0.39% of tested persons) were diagnosed with HIV for the first time. The success of the pilot program has resulted in a grant from industry to sustain the routine, opt-out, rapid testing program beyond the IIDDEAL grant period. We anticipate that new cases will be found at Fulton County Jail during our ***Preliminary Planning for*** ***SUCCESS*** project. Both newly and previously diagnosed HIV+ detainees will be invited to participate in our feasibility study.

**Diversity in duration of detainment.** Prior research in jails suggests that flexibility in the timing of enrollment in SBCM with respect to release from incarceration will maximize recruitment into the proposed study. For jail detainees in EnhanceLink, time before release was highly unpredictable; LOS for clients ranged from <5 hours to >1 year. Similar observations in IIDDEAL (see Figure 1A) suggest that interventions designed to link releasees to medical care must implement quick capture and aggressive follow through for those with extremely short stays. At the same time, interventions must be flexible to account for persons with unexpectedly prolonged stays. Timing interventions to occur when release is imminent may work for sentenced inmates in prison, but will not work for detainees in jail.

**
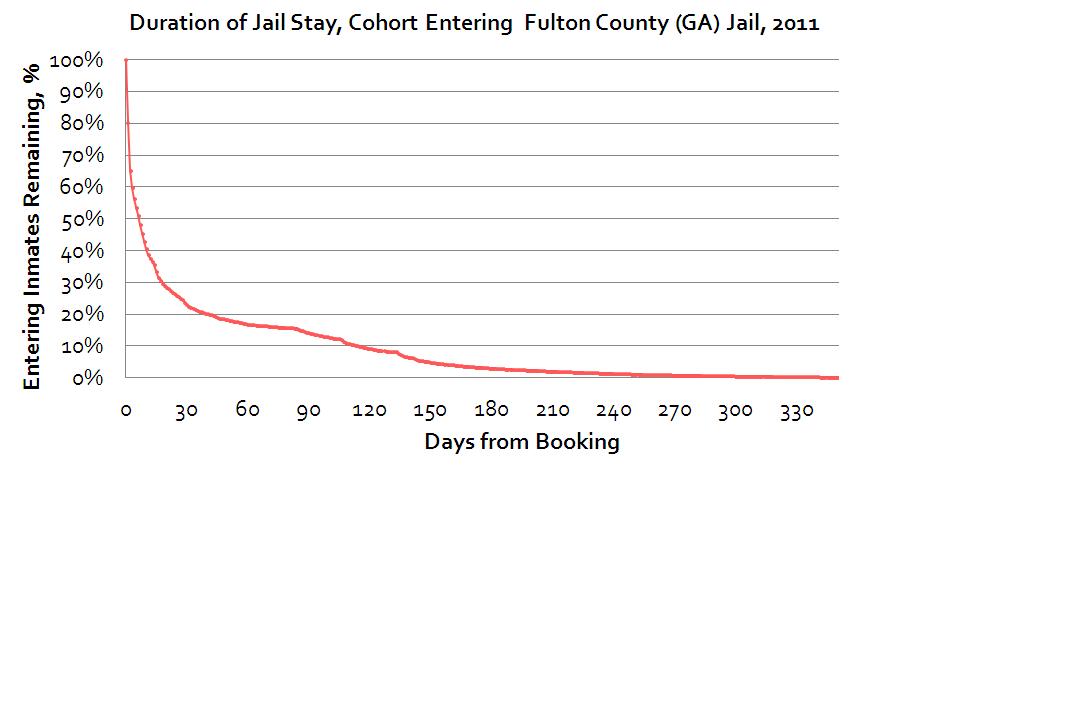
**
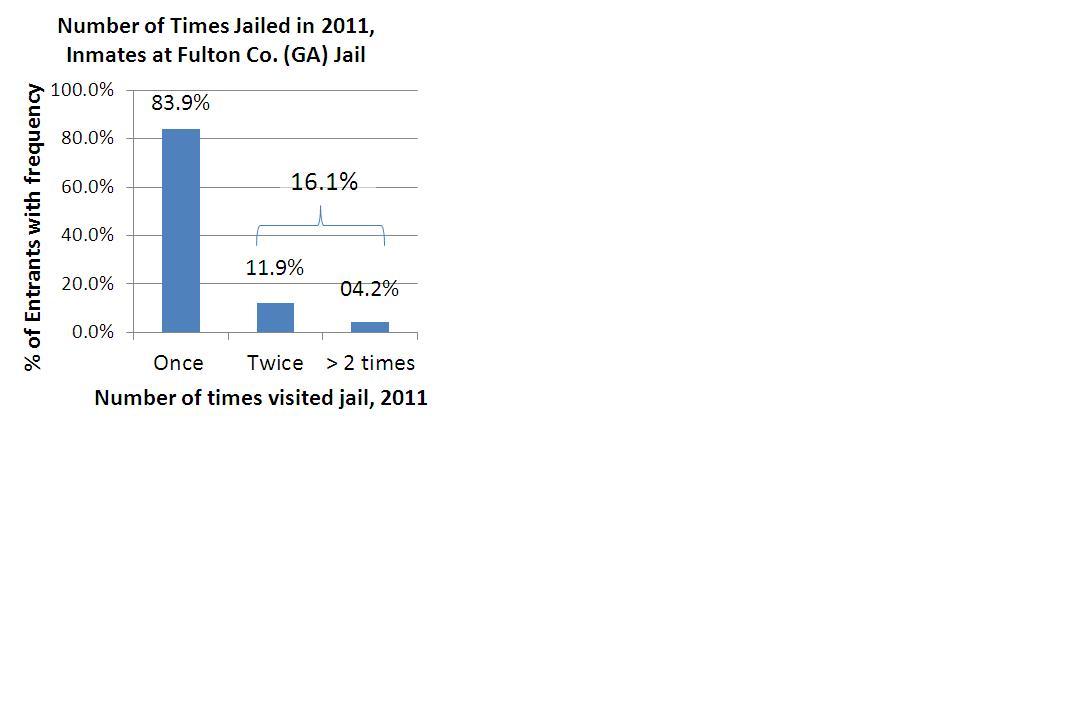


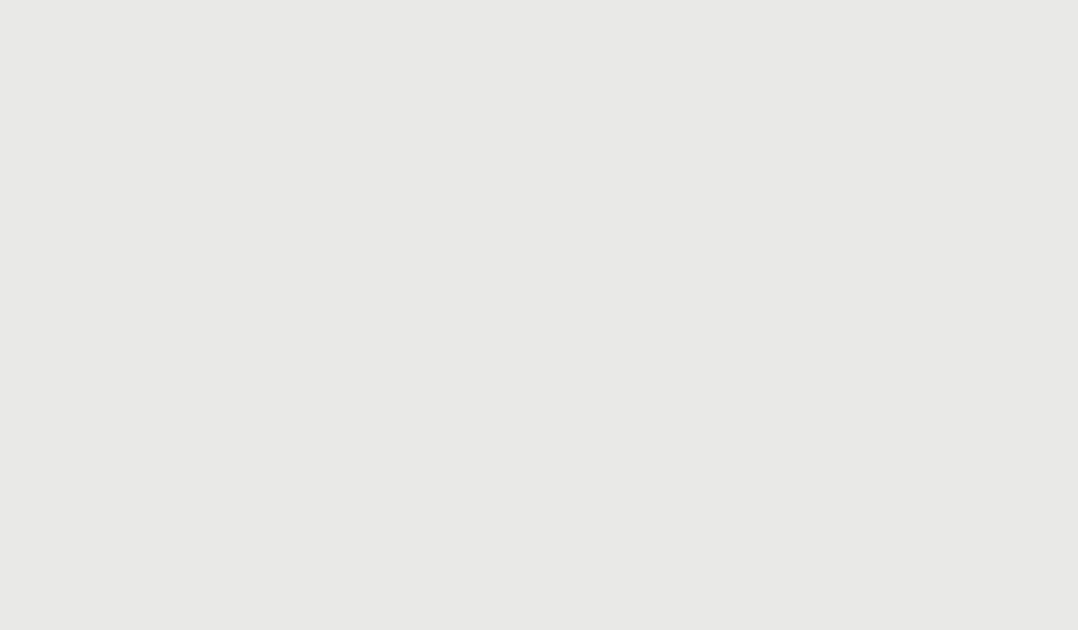


Jail Population Dynamics.

A. Length of Stay

B. Frequency of Return during One Calendar Year

A.

B.

**Figure 1**

**vii. Development of SUCCESS.** To modify *ARTAS* for the target population, Dr. Spaulding has worked with behavioral scientist and Emory faculty member Dr. Wingood to implement her adaptation protocol, which modifies interventions that worked in one setting for use in another.[^64^](#_ENREF_64) Part of this adaptation involved holding focus groups with four groups of former jail detainees in Atlanta who were HIV+. Former participants of the *HOPE* study composed focus group #1. Focus group #1 discussed adherence to ART in jail and barriers that hinder engagement in medical treatment post jail; they judged that the adaptation of an *ARTAS*-like intervention to engage jail releasees in care would be warranted. Complexities associated with their post-jail experiences suggested a need for an extra session of an *ARTAS*-like program and improved communication after release, a time of high mobility. Our study team decided to modify *ARTAS* by increasing the intensity and including cell phones to improve communication. Focus group #2 dealt with cell phones; participants endorsed the use of cell phones to facilitate linkage by improving their ability to call clinics. All participants had owned a cell phone at one time; 60% knew how to text. Participants indicated that airtime was valued as compensation. Focus Group #3 watched a dramatization of an enhanced *ARTAS* intervention and provided feedback. Based on their feedback, we brought Dr. Rapp, an expert in SBCM who helped develop *ARTAS*, to Atlanta to help us draft the Client Services Guide specifically for **SUCCESS** .To improve the dialogue between CM and client when discussing complex issues, Dr. Rapp helped us develop visual aids—low literacy diagrams of the concepts of **SUCCESS.** Focus group #4 watched a dramatization of **SUCCESS** sessionsand provided ample feedback. They endorsed the CM bringing visual aids and said that CMs need to be “persistent but not overwhelm” the client. Feedback from all four focus groups helped us finalize the Client Services Guide**.**

**Feasibility of recruiting.** Some previous studies of HIV+ persons released from incarceration have suffered from low enrollment.[^27^](#_ENREF_27) In order to demonstrate the feasibility of recruiting soon-to-be-released Atlanta detainees in research, we conducted a 2-week pilot study in August 2011 among HIV+ persons transitioning out of Atlanta jails.This pilot, *Text2Link*, provided cell phones and 1 week’s worth of airtime at the time of release if the participants consented to be contacted by study staff. Texting with our staff was compensated with a second week of airtime. We were able to recruit 3-4 persons a week for a total of 7 persons in 10 days of recruiting. All texted with study staff once out; 100% received the additional compensation. Our proposed study will build on this *Text2Link* pilot and demonstrate persons can be recruited specifically for an intervention to improve linkage to medical care.

.

**2. Design**

**a. Sample**

- Population:
  - We anticipate recruiting 42 persons age 18 years or older, HIV infected, and recently entering Fulton County Jail. Based on our prior work at the jail, we anticipate that likely 85% of participant will be male, 90% Black, and highest educational attainment will be less than a GED or high school diploma for 69%.
- Safeguards in place for this vulnerable population:
- Study will be introduced by usual healthcare staff; they will make referral of a potential subject to the study team only if the potential participant is interested and signs a release of information to permit forwarding of contact information to the study staff. Study staff will visit jails and will speak with interested detainees referred by the healthcare staff.
  - Informed consent process (see below) will let persons know benefits and risks of participating and that disenrollment will be possible at any step.
  - Intervention will take place in settings where the study staff can maintain auditory and visual privacy.
  - Study data (e.g., demographic survey) will be kept confidential.
- Inclusion criteria:
  - HIV infected (HIV+); age of or over 18 years;
  - Mentally able to give consent; understand spoken English;
  - Detained or sentenced in the Fulton County, GA jail; and
  - Likely to leave within 6 weeks.
- Exclusion criteria:
  - Unable to give consent because of mental illness or inebriation;
  - A recent participant in a randomized trial conducted by the investigators of an intervention to increase retention in HIV care (e.g., ARTAS)

**b. Setting**

Location of study procedures and/or data collection: Recruitment will occur in Fulton County Jail. Delivery of the first part of the intervention will be in this jail; the remaining steps will occur in the community, such as in the waiting rooms of HIV clinic offices or other locations convenient for the participants. The study staff will take care that confidentiality of health information will be maintained.

**c. Recruitment: site, procedure, methods**

Jail healthcare staff can approach HIV positive inmates and ask if they would like to have study staff members talk to them about a potential project. If so, they will have potential participants sign a release of information form that will permit them to convey the name of the inmate to study staff. Jail healthcare staff will have a dedicated phone number to make participant referrals to the study staff. A member of the study staff will go to the jail to evaluate the potential participant for eligibility for participation in the study, explain the program, and obtain written informed consent to participate in the study and to release medical records from the jail and from future sites of HIV care. The study staff member will determine if the potential participant has a cell phone on the outside and inform him/her that study incentives will preferentially be paid in cell phone minutes.

All potential participants, regardless of whether they consent to participate in the study, will be given information about where to obtain HIV healthcare near where they plan to settle after release, such as a HRSA/ Ryan White supported clinic. Any written information on how to obtain benefits, medications, and substance abuse care will be written at a sixth grade reading level.

- Plans to monitor equitable recruitment of subjects: N/A for this demonstration project

**d. Study Procedures**

- Study design is a longitudinal cohort study of individuals receiving a behavioral intervention. We will obtain baseline data (demographical data, data extracted from jail medical chart review); deliver an intervention designed to improve linkage to medical care; and then extract data from medical records in the community.
- The research material will be responses to self-report questionnaires, and abstracted data from participants’ medical charts. Records maintained at Emory University will be kept electronically on a strong password protected computer in a locked room. Each record will be assigned a unique code that is, in and of itself, non-identifying. The link to the code will be on a separate sheet of paper without reference to HIV, and will be kept separately in a folder protected by a password known only to the PI and the project coordinator.
- Access to medical records of participants will be limited to those staff responsible for performing chart abstractions. Data from chart abstractions will be stored at Emory and will be identified only by a unique code.
- Procedures for subjects
  - Data collection procedures: the case manager will administer a baseline demographic survey using pen and paper. If the participant has difficulties with reading, the case manager will read the questions to the subject.
  - Other study related interactions:

The **SUCCESS** intervention that has been adapted for the target population is based on ARTAS (see above.). The four core elements of *ARTAS* (1. developing working relationship with client, 2. identifying client’s goals, 3. encouraging client’s strengths, 4. meeting in the client’s environment) will be the core elements of **SUCCESS**.[^65^](#_ENREF_65) Together, a CM and client will navigate through a fragmented and potentially confusing system of diverse resources and agencies. The philosophy of SBCM is to have clients set goals because, while the ultimate goal of **SUCCESS** is to link HIV+ persons to medical care, the subjects

**
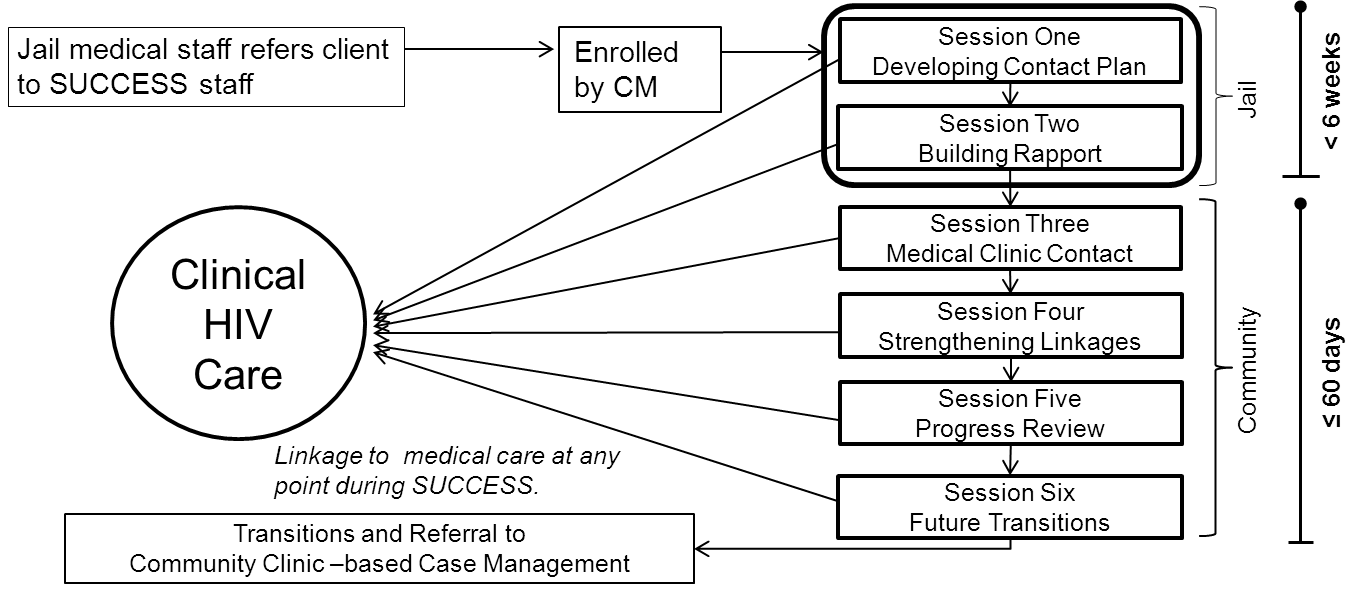
**initially may not share this goal. The *ARTAS* intervention showed that, through engagement with CMs, clients were drawn to greater participation in medical care.[^7^](#_ENREF_7) The **SUCCESS** intervention will follow the objectives and strategies of the *ARTAS* manual distributed by the CDC,[^66^](#_ENREF_66) with 3 adaptations: 6 rather than 5 *ARTAS*-like SBCM sessions, the use of mobile phones and texting, and the use of low literacy audio-visual aids in the sessions. The **SUCCESS** intervention will be delivered by the study’s three CMs, with the first 2 sessions (or “contacts”) designed to occur in jail, pre-release. Release dates in a jail can be unpredictable. If a study participant is released prior to completion of the first 2 contacts, those contacts will occur in the community. Subsequent contacts are designed to occur in the community post release, but they can take place in jail if release is delayed. **Session 1** will begin with development of a “contact plan”, a systematic and structured plan to help a participant access medical services. A CM will spend approximately 1.5 to 2 hours face-to-face with a participant for the provision of three initial services: 1) Completion of forms so that HIV drug benefits can be initiated/continued including AIDS Drug Assistance Program (ADAP) or indigent drug assistance programs, insurance and Medicaid forms, and forms to obtain a non-correctional picture ID and a birth certificate; 2) Making referrals to local substance abuse treatment programs if drug or alcohol dependency is an issue for a participant and to mental health care if the mental health screen was positive; and 3) Confirming a participant’s choice of a clinic where high quality, specialty HIV care can be obtained, tailored to a participant’s needs or preferences (e.g. the choice between a neighborhood clinic to facilitate access versus a clinic across town to facilitate privacy). A discussion of how to arrange transportation and engage in services after jail discharge will also be held. A participant can always choose later to change medical homes. Rapport building and a personal strength assessment will take place at **Session 2** which will be ideally scheduled before or on the day of release. A key element of the intervention is the social support that will be offered to a participant to help him or her cope with illness and other stressful situations including having his or her CM meet a detainee at the jail’s exit door, since such meetings have been associated with improved linkage outcomes..[^57^](#_ENREF_57)^,^[^67^](#_ENREF_67) At the end of Session 2, a participant and his or her CM will decide where to meet upon release.

Figure 2. Client Flow in **SUCCESS**

Post-release sessions will ideally take place in the medical clinic (such as the Ponce Center or the AID Atlanta medical clinic) but can occur at another, mutually agreed upon, safe, public location, such as a coffee shop; public transportation to the meetings will be reimbursed. **Session 3** will occur around the time of a participant’s release from jail, preferably on the day of release. It will focus on reviewing how to make contact with the medical clinic if no medical visit has yet occurred. In addition, a CM will confirm that a participant has access to a cell phone. Many releasees will have personal cell phones available to them; we will provide basic cell phones for those without mobile phones. A CM will encourage a participant to set reminders on his or her phone to assist with keeping appointments or taking medications. Unless a participant opts out, his or her CM will arrange for the delivery of both automated and personalized messages to help with the achievement of goals.[^68^](#_ENREF_68) From the focus groups, we learned releasees may want text messages to remind them of clinic appointments, to hold them accountable in challenging situations such as dealing with negative peer pressure, to serve as periodic check-ups for an update on status, and for general words of encouragement. Using the expertise of Dr. Ann Kurth, we will design these messages tailored to a participant’s stated needs and literacy level. For example, illiterate participants may receive text-to-phone audio messages. The study team will initiate message once per week for the 60 days of the post release period. **Session 4** will focus further on linkage to services that can reduce risk in problematic areas, such as treatment for substance abuse that hinders medication adherence or mental health treatment that might interfere with maintaining employment. With the help of his or her CM, a participant will map out the steps needed to gain entry into treatment programs. At **Session 5,** a participant and his or her CM will review progress on how well the contact plan was achieved. **Session 6** will focus on disengagement and transition to the clinic’s usual case management. The typical participant will receive 6 sessions, all of which should be completed within the first 60 days post-release. Missed sessions can be rescheduled, as soon as is possible. After the 6 initial sessions, a participant may informally contact his or her CM by phone or in person for review of the contact plan, but if more intense case management is needed, the study CM will make a referral to case management through a participant’s medical home. Although sessions 1 and 2 are designed to be delivered in jail and sessions 3 through 6 in the community, the program includes the necessary flexibility so that most sessions could be delivered in or out of the jail setting. This is necessary given the unpredictability of jail release dates and the potential for re-incarceration. Text messaging and phone calls to a CM will occur throughout the period of SBCM delivery.

2. Describe total respondent burden: the baseline demographic data will take less than 30 minutes to complete. The intervention will involve 6-20 hours of face time with a case manager.

**e. Measures**

***Process measurement*:** we will determine if 42 participants can be enrolled in three months and that SBCM can be delivered according to the SUCCESS intervention protocol. Dr. Frew, assisted by Ms. Bowden, will oversee evaluation of the CMs. We anticipate the 2 CMs will each be assigned 6-8 new clients a month (a total of 14 assigned per month); clients will stay in the “active phase” for 2 months after discharge. At bi-weekly meetings, Ms. Bowden will determine if there is balance between the caseloads. Ms. Bowden will review data on: (1) the number of intervention sessions that each client receives, and (2) any make-up sessions if initial visits are missed. We will monitor fidelity to the intervention protocol. With permission of the client, Ms. Bowden will periodically observe sessions and afterwards discuss her observations with the CMs. CMs will document each contact with clients on their caseload, recording specific information about a session including: date, location and length of contact, presence of other persons or not, client use of cell phones, referrals to clinical and social services that are followed through and intensity of referral (e.g., phone call only or went with client to referral). Further, CMs document what barriers to linking with medical care were discussed during each session.

Dr. Frew will oversee the conduction of independent observation of staff-client interactions. The basis for these observations will be the ARTAS-like Case Management Fidelity Scale consisting of five areas that are central to the delivery of strengths-based interventions such as SUCCESS. These areas include: focus on strengths, encouragement of client-driven activities, relationship building, assertive outreach, and use of resources. She will rate CMs on 29 specific tasks in the five areas using a 5 point Likert-like scale that asks whether she Disagree Strongly (1) to Agree Strongly (5) that a CM has delivered a task with full fidelity. Using a random number generator to select sessions, we will audiotape a random sample of 10% of sessions in the community (and in the jail when policy permits audiotaping—jail policy can change with time). Dr. Frew and Project Coordinator Bowden, assisted by Dr. Rapp, will monitor the conduction of the intervention activities by reviewing the audio-taped sessions on a weekly basis. Ms. Bowden will provide feedback to CMs based on their scores on the fidelity scale. Prompt discussion of fidelity scores has been demonstrated to be an effective tool in improving adherence to intervention protocols. Additionally, the CMs will present cases at bi-weekly supervisory sessions. These sessions will allow Ms. Bowden the opportunity to monitor adherence to SUCCESS, provide a teaching opportunity in areas she feels need additional attention, address and resolve any problems that may arise during the intervention, and ensure fidelity to the protocol.

***Outcomes measurement:*** The primary outcome will be whether a participant linked to community HIV care and, if it occurs, how long before a linkage occurred, with the clock starting at the initial release from jail. Linkage to care will be defined as a visit to an HIV provider that entails the acquisition of an HIV viral load and CD4 cell count. This outcome will be assessed at 3 months and will be measured in days between initial jail discharge and laboratory draw. This measurement will be adjusted for the number of days at liberty, since participants may return to jail one or more times in those 3 months post initial release. The interval of 3 months post initial release, rather than a shorter period, is warranted because participants who had a medical visit within a month prior to incarceration and, upon release, returned to the clinic within 3 months of their last visit, would still be considered linked to care by national performance guidelines[^69^](#_ENREF_69) **Chart review.** The study team will review the jail medical records and the community HIV medical clinic records at 3 months in order to gather data on whether a face to face visit occurred with a provider and whether laboratory tests (CD4 and HIV RNA) were ordered.

1. **Risks to Participation**

Potential discomfort or risk to a subject includes 1) psychological distress if a participant feels coerced to participate, and 2) breach of confidentiality of health information. This research does not adversely affect a participant’s rights and welfare, provided that the confidentiality of identifying information is maintained. The two potential risks listed above will be addressed in the following manner:

1. Subjects may experience psychological stress if they feel coerced to participate. However, the likelihood of this risk is small. When the study team solicits informed consent, potential participants will be told that their enrollment is voluntary. Furthermore, enrolling in the study will not affect the conditions of any parole or probation. Participant incentives in this study are nominal and will be given to participants only after reentry to the community. Participants may separate from the study at any point without any penalty.
2. To minimize the risk of a breach of confidentiality of any medical data (including HIV status and other sensitive information), participants will be assigned study ID numbers. These numbers, rather than personally-identifiable information, will be associated with data. Confidential information, including health information, will be stored in a locked drawer in a locked study office. Only study staff will have access to the keys. The audio recordings of the focus group will be destroyed at the completion of the study. Standards for privacy of individually identifiable health information under the Health Insurance Portability and Accountability Act of 1996 will be met. HIPAA Authorization documentation will be incorporated into the consent forms that will be obtained from participants. The only information that will not be kept confidential is if volunteers or members of the study staff believe that program participants are a danger to themselves or others; or if minor children are under control of the participant, and there is reasonable evidence that the children are at risk.
3. **Benefits to Subject**

The findings of this study will help inform future adaptations of the intervention to jails in urban settings. These future adaptations could help inform policy makers of structural interventions to improve linkage to care for HIV-positive jail releasees. Population subgroups disproportionately affected by incarceration will be the greatest beneficiaries. In addition to receiving a small cash incentive, individual study participants may benefit from improved linkage to care, as facilitated by the SBCM intervention sessions.

1. **Data Analysis**

***Sample Size Determination***

No sample size calculations have been performed because this pilot study has not been powered to achieve outcomes that are statistically significant. We have chosen a sample size of forty-two participants because it is a reasonable number of persons to pilot the intervention/new protocol, SUCCESS, to show feasibility.

***Endpoint Definitions***

Time to linkage to care will be defined as the time from release to visit with an HIV provider, as evidenced by laboratory results indicating measurement of an HIV viral load and/or CD4 count.

***Statistical Analyses***

Linkage will be assessed for each individual at 3 months post-release from their index incarceration—how much time passed after release before the participant kept an appointment in an HIV clinic. Subjects will be followed from the day they are released, and the number of releasees who have linked to care every 30 days will be assessed at 3 months. The day of the first laboratory draw post-release will be considered the time point at which linkage occurs. A Kaplan-Meier curve with associated 95% confidence interval will be used to estimate the linkage rates for the participants. The proportion of persons retained in care will be presented with its associated 95% confidence interval.

1. **Training**

Dr. Rapp has conducted 2 days of face-to-face training with the project coordinator, Ms Bowden, and Ms. Mustaafaa. The newly hired study CM, trained at the minimum at a Bachelors level in an appropriate field, and with case management experience, will undergo training by Dr. Rapp (via Skype) and Ms. Bowden to ensure that they understand the basic principles of the *ARTAS* approach and can implement the intervention with fidelity to the conceptual model and the protocols. All CMs will also receive training in administration of the informed consent process and a brief baseline survey. Having the intervention staff, rather than a separate, independent assessment team, administer the baseline survey is a concession that reflects the complexity of having multiple staff members connect with clients within the jail. At the end of the training period, all intervention staff will demonstrate comprehension of the three principles of SBCM (and how they relate to the factors inherent in the conceptual model. CMs will have a refresher training session once during the intervention period.

1. **Data Safety and Monitoring Plan**

A formal DSMP is not applicable for this demonstration project.

1. **Confidentiality**

All participant information will be kept private. Upon enrollment in the study, the participant will be assigned a study identification number in which all information will be tracked. Participant information will be stored in locked cabinets and on password protected computers. Access to the project data management system will only be given study staff/researchers with individual, private logins and passwords.

1. **Informed Consent**

Upon successful completion of an eligibility assessment, study participants will be asked by study staff to participate in the study. Study participants will be given a written Informed Consent Form which will be read to them by the study staff person at which time if the participants agree, will sign. Study participants may opt out of the study at any time. Site staff has been instructed not to recruit inebriated inmates. Once enrolled into the study, participants will be assigned to the case manager who will provide all case management services.

1. **Plans to Inform Participants of New Findings**

The study researchers will disseminate findings from this study in both scientific and lay formats. The study staff persons or community base partners will attempt to contact clients about results that might affect their health.

| Table. Project Timeline: ***Preliminary Planning for SUCCESS*** | Q1 | Q2 | Q3 |
| --- | --- | --- | --- |
| Finalize IRB Approval & Certificate of Confidentiality. Hire/train staff. | √ |  |  |
| Recruit 14 subjects/month, start SBCM in jail x 3 months (N=42). |  | √ |  |
| Deliver SBCM for 60-day post jail release. |  | √ | √ |
| Conduct 3 month chart review of participants. |  |  | √ |

REFERENCES

**1.** Westergaard RP, Kirk GD, Richesson DR, Galai N, Mehta SH. Incarceration Predicts Virologic Failure for HIV-Infected Injection Drug Users Receiving Antiretroviral Therapy. *Clinical Infectious Diseases.* 2011;53(7):725-731.

**2.** Spaulding AC, Seals RM, Page MJ, Brzozowski AK, Rhodes W, Hammett TM. HIV/AIDS among inmates of, and releasees from, US correctional facilities, 2006: declining share of epidemic but persistent public health opportunity. *PLoS ONE [Electronic Resource].*4(11):e7558.

**3.** U.S. Preventative Services Task Force. Screening for gonorrhea. <http://www.ahrq.gov/clinic/uspstf/uspsgono.htm>. Accessed 07/22/2008.

**4.** Maruschak LM, Beavers R. HIV in prisons, 2007-8. *Bulletin of the Bureau of Justice Statistics.* 2009. <http://bjs.ojp.usdoj.gov/content/pub/pdf/hivp08.pdf>. Accessed 01/16/2010.

**5.** Harrison PM, Beck AJ. Prison and jail inmates at midyear 2005. *Bulletin of the Bureau of Justice Statistics.* 2006. <http://bjs.ojp.usdoj.gov/content/pub/pdf/pjim05.pdf>. Accessed 01/02/2007.

**6.** Rapp CA, Goscha RJ, eds. *The strengths model: case management with people with psychiatric disabilities, 2nd ed* New York City: Oxford University Press 2006.

**7.** Gardner LI, Metsch LR, Anderson-Mahoney P, et al. Efficacy of a brief case management intervention to link recently diagnosed HIV-infected persons to care. *AIDS.* Mar 4 2005;19(4):423-431.

**8.** Craw JA, Gardner LI, Marks G, et al. Brief strengths-based case management promotes entry into HIV medical care: results of the antiretroviral treatment access study-II. *JAIDS.* Apr 15 2008;47(5):597-606.

**9.** Flanigan TP, Zaller N, Beckwith CG, et al. Testing for HIV, Sexually Transmitted Infections, and Viral Hepatitis in Jails: Still a Missed Opportunity for Public Health and HIV Prevention. *JAIDS.* 2010;55:s78-s83.

**10.** Gardner EM, McLees MP, Steiner JF, Del Rio C, Burman WJ. The spectrum of engagement in HIV care and its relevance to test-and-treat strategies for prevention of HIV infection. [Review]. *Clinical Infectious Diseases.* 2011;52(6):793-800.

**11.** Cohen SM, van Handel MM, Branson BM, et al. Vital Signs: HIV Prevention Through Care and Treatment—United States *MMWR.* 2011;60(47):1618-1623.

**12.** Hall HI, Gray KM, Tang T, Li J, Shouse L, Mermin J. Retention in Care of Adults and Adolescents living with HIV in 13 U.S. Areas. *JAIDS Journal of Acquired Immune Deficiency Syndromes.*

**13.** Guerino P, Harrison PM, Sabol WJ. Prisoners in 2010 (revised). *Bulletin of the Bureau of Justice Statistics.* 2011. <http://bjs.ojp.usdoj.gov/content/pub/pdf/p10.pdf>.

**14.** Spaulding A, Stephenson B, Macalino G, Ruby W, Clarke JG, Flanigan TP. Human immunodeficiency virus in correctional facilities: a review. *Clinical Infectious Diseases.* Aug 1 2002;35(3):305-312.

**15.** World Prison Brief. <http://www.prisonstudies.org/info/worldbrief/>. Accessed 12/17/2011.

**16.** US Department of Health and Human Services. Recommendations for Case Management Collaboration and Coordination in Federally Funded HIV/AIDS Programs. 2008. <http://www.cdcnpin.org/scripts/features/CaseManagement.pdf>.

**17.** Thompson MA, Mugavero MJ, Amico KR, et al. Guidelines for Improving Entry Into and Retention in Care and Antiretroviral Adherence for Persons With HIV: Evidence-Based Recommendations From an International Association of Physicians in AIDS Care Panel. *Annals of Internal Medicine.* 2012;156(11):817-833.

**18.** Malitz FE, Eldred L. Evolution of the special projects of national significance prevention with HIV-infected persons seen in primary care settings initiative. *AIDS & Behavior.* Sep 2007;11(5 Suppl):S1-5.

**19.** Cheever LW. Engaging HIV-infected patients in care: their lives depend on it. *Clinical Infectious Diseases.* 1500;44(11):1500-1502.

**20.** Fleishman JA, Yehia BR, Moore R, Korthuis PT, Gebo KA, For the HIV Research Network. Establishment, retention, and loss to follow-up in outpatient HIV care. *JAIDS.* 2012;60(3):249-259.

**21.** Rosen DL, Golin CE, Schoenbach VJ, et al. Availability of and access to medical services among HIV-infected inmates incarcerated in North Carolina county jails. *Journal of Health Care for the Poor & Underserved.* Aug 2004;15(3):413-425.

**22.** Baillargeon J, Giordano TP, Rich JD, et al. Accessing antiretroviral therapy following release from prison. *JAMA.* Feb 25 2009;301(8):848-857.

**23.** Clements-Nolle K, Marx R, Pendo M, et al. Highly active antiretroviral therapy use and HIV transmission risk behaviors among individuals who are HIV infected and were recently released from jail. *American Journal of Public Health.* Apr 2008;98(4):661-666.

**24.** Nunn A, Cornwall A, Fu J, Bazerman L, Loewenthal H, Beckwith C. Linking HIV-positive jail inmates to treatment, care, and social services after release: results from a qualitative assessment of the COMPASS Program. *Journal of Urban Health.*87(6):954-968.

**25.** Gamble VN. Under the shadow of Tuskegee: African Americans and health care. *American Journal of Public Health.* Nov 1997;87(11):1773-1778.

**26.** Pai NP, Estes M, Moodie EE, Reingold AL, Tulsky JP. The impact of antiretroviral therapy in a cohort of HIV infected patients going in and out of the San Francisco county jail. *PLoS ONE [Electronic Resource].* 2009;4(9).

**27.** Wohl D, Scheyett A, Golin C, et al. Intensive Case Management Before and After Prison Release is No More Effective Than Comprehensive Pre-Release Discharge Planning in Linking HIV-Infected Prisoners to Care: A Randomized Trial. *AIDS and Behavior.* 2011;15(2):356-364.

**28.** Stephenson BL, Wohl DA, McKaig R, et al. Sexual behaviours of HIV-seropositive men and women following release from prison. *International Journal of STD & AIDS.* Feb 2006;17(2):103-108.

**29.** Grinstead OA, Zack B, Faigeles B, Grossman N, Blea L. Reducing Postrelease HIV Risk among Male Prison Inmates: A Peer-Led Intervention. *Criminal Justice and Behavior.* December 1, 1999 1999;26(4):453-465.

**30.** Cohen MS, Chen YQ, McCauley M, et al. Prevention of HIV-1 Infection with Early Antiretroviral Therapy. *New England Journal of Medicine.* 2011;365(6):493-505.

**31.** Prevention CfDCa. Zidovudine for the prevention of HIV transmission from mother to infant. *MMWR.* 1994;43(16):285-287.

**32.** Sullivan P, Kayitenkore K, Chomba E, et al. Reduction of HIV transmission risk and high risk sex while prescribed ART: results from discordant couples in Rwanda and Zambia. *16th Conference on Retroviruses and Opportunistic Infections*. Montreal, Canada. 2009.

**33.** Kitahata MM, Gange SJ, Abraham AG, et al. Effect of early versus deferred antiretroviral therapy for HIV on survival. *New England Journal of Medicine.* Apr 30 2009;360(18):1815-1826.

**34.** May M, Sterne JA, Sabin C, et al. Prognosis of HIV-1-infected patients up to 5 years after initiation of HAART: collaborative analysis of prospective studies. *AIDS.* May 31 2007;21(9):1185-1197.

**35.** Scott KC, Philip S, Ahrens K, Kent CK, Klausner JD. High prevalence of gonococcal and chlamydial infection in men who have sex with men with newly diagnosed HIV infection: an opportunity for same-day presumptive treatment. *Journal of Acquired Immune Deficiency Syndromes: JAIDS.* May 1 2008;48(1):109-112.

**36.** National HIV/AIDS Strategy for the United States. 2010; <http://www.whitehouse.gov/sites/default/files/uploads/NHAS.pdf>. Accessed 12/03/2010.

**37.** Fleisher P, Henrickson M. Towards a Typology of Case Management. <ftp://ftp.hrsa.gov//hab/Typology.pdf>. Accessed 02/01/2010.

**38.** Healey KM. Case management in the criminal justice system- reseach in action. 1999. <http://www.ncjrs.gov/pdffiles1/173409.pdf>. Accessed 03/20/2010.

**39.** Weick A, Rapp C, Sullivan WP, Kisthardt W. A Strengths Perspective for Social Work Practice. *Social Work.* 1989;34(4):350-354.

**40.** Ward T, Brown M. The good lives model and conceptual issues in offender rehabilitation. Routledge2004.

**41.** Bandura A. Self-efficacy:toward a unifying theory of behavioral change. *PsychRev.* Mar 1977;84(2):191-215.

**42.** Vaughan-Sarrazin MS, Hall JA, Rick GS. Impact of case management on use of health services by rural clients in substance abuse treatment. *Journal of Drug Issues.* 2000;30(2):435-463.

**43.** Strathdee SA, Ricketts EP, Huettner S, et al. Facilitating entry into drug treatment among injection drug users referred from a needle exchange program: Results from a community-based behavioral intervention trial. *Drug Alcohol Depend.* Jul 27 2006;83(3):225-232.

**44.** Rapp RC, Otto AL, Lane DT, Redko C, McGatha S, Carlson RG. Improving linkage with substance abuse treatment using brief case management and motivational interviewing. *Drug Alcohol Depend.* Apr 1 2008;94(1-3):172-182.

**45.** Rapp RC, Siegal HA, Li L, Saha P. Predicting postprimary treatment services and drug use outcome: a multivariate analysis. *Am J Drug Alcohol Abuse.* Nov 1998;24(4):603-615.

**46.** Siegal HA, Li L, Rapp RC. Case management as a therapeutic enhancement: impact on post-treatment criminality. *J Addict Dis.* 2002;21(4):37-46.

**47.** Bell C, Metsch LR, Vogenthaler N, et al. Never in care: characteristics of HIV-infected crack cocaine users in 2 US cities who have never been to outpatient HIV care. *JAIDS* 2010;54(4):376-380.

**48.** Metsch LR, Bell C, Pereyra M, et al. Hospitalized HIV-infected patients in the era of highly active antiretroviral therapy. *American Journal of Public Health.* Jun 2009;99(6):1045-1049.

**49.** Stoll P, Duncan T, Marr O. The Diffusion of ARTAS: What CDC Plans to Do. Paper presented at: CDC National HIV Prevention Conference; August 14-17, 2011; Atlanta GA.

**50.** Spaulding AC, Arriola KR, Ramos KL, et al. Enhancing linkages to HIV primary care in jail settings. *Journal of Correctional Health Care.* 2007;12(2):93-128.

**51.** Spaulding AC, Arriola KRJ, Hammett T, Kennedy S, Tinsley M. Rapid HIV testing In rapidly released detainees: next steps. *Sexually Transmitted Diseases* 36(suppl 2):s34-s36.

**52.** DeVoux A, Beckwith C, Avery A, et al. Early Identification of HIV: Empirical Support for Jail-Based Screening. *PLoS ONE [Electronic Resource].* 2012;7(5):e37603.

**53.** Draine J, Ahuja D, Altice FL, et al. Strategies to enhance linkages between care for HIV/AIDS in jail and community settings. *AIDS Care.* 2011;23(3):366-377.

**54.** Spaulding AC, Booker CA, Freeman SH, et al. and The EnhanceLink Study Group. Jails, HIV testing and linkage to care services: an overview of the EnhanceLink project. *AIDS and Behavior (in press).*

**55.** Spaulding AC, Messina LC, Kim BI, et al. Planning for Success Predicts Virus Suppressed: Results of a Non-Controlled, Observational Study of Factors Associated with Viral Suppression among HIV-positive Persons Following Jail Release. *AIDS and Behavior (in press).*

**56.** Stein MS, Spaulding AC, Cunningham M, et al. HIV-positive and in jail: race, risk factors, and prior access to care. *AIDS and Behavior (in press).*

**57.** Booker CA, Flygare CT, Solomon L, et al. and The EnhanceLink Study Group. Linkage to HIV care for jail detainees: findings from the first 30 days after release. *AIDS and Behavior (in press).*

**58.** Chen N, Meyer J, Avery A, et al. Adherence to HIV treatment and care among previously homeless jail detainees. *AIDS and Behavior.* 2011:1-13.

**59.** Willers DM, Peipert JF, Allsworth JE, Stein MD, Rose JS, Clarke JG. Prevalence and predictors of sexually transmitted infection among newly incarcerated females. *Sexually Transmitted Diseases.* Jan 2008;35(1):68-72.

**60.** Kahn RH, Voigt RF, Swint E, Weinstock H. Early syphilis in the United States identified in corrections facilities, 1999-2002. *Sexually Transmitted Diseases.* Jun 2004;31(6):360-364.

**61.** Weinstock H, Berman S, Cates W, Jr. Sexually transmitted diseases among American youth: incidence and prevalence estimates, 2000. *Perspectives on Sexual & Reproductive Health.* Jan-Feb 2004;36(1):6-10.

**62.** Spaulding AC, Pinkerton S, Superak H, et al. Cost Analysis of Enhancing Linkages to HIV Care Following Jail: A Cost-Effective Intervention. AIDS and Behavior.(in press).

**63.** Spaulding A, Cook J, Bowden C, et al. There’s a need and a way; where’s the will? rapid HIV testing for jail entrants. *CDC HIV Prevention Conference*. Atlanta, GA. 2011.

**64.** Wingood GM, DiClemente RJ, Wingood GM, DiClemente RJ. The ADAPT-ITT model: a novel method of adapting evidence-based HIV Interventions. *JAIDS.* Mar 1 2008;47 Suppl 1:S40-46.

**65.** Anti-Retroviral Treatment and Access to Services (ARTAS): An individual-level, multi-session intervention for people who are recently diagnosed with HIV. *Implementation Manual.* <http://www.cdc.gov/hiv/topics/cba/pdf/artas_implementation_manual.pdf>. Accessed 08/20/2011.

**66.** Anti-Retroviral Treatment and Access to Services (ARTAS) An individual-level, multi-session intervention for people who are recently diagnosed with HIV Implementation Manual. Published May 2011. Available: <http://www.cdc.gov/hiv/topics/cba/pdf/artas_implementation_manual.pdf> Accessed: 20 August 2011.

**67.** Jacob Arriola KR, Braithwaite RL, Holmes E, Fortenberry RM. Post-release case management services and health-seeking behavior among HIV-infected ex-offenders. *Journal of Health Care for the Poor & Underserved.* Aug 2007;18(3):665-674.

**68.** Dunbar PJ, Madigan D, Grohskopf LA, et al. A two-way messaging system to enhance antiretroviral adherence. *Journal of the American Medical Informatics Association.* 2003;10(1):11-15.

**69.** US Department of Health and Human Services. Health Resources and Services Administration HIV/AIDS Bureau Performance Measures, 2011. Available: <http://hab.hrsa.gov/deliverhivaidscare/clinicalguide11/>. Accessed: 28 August 2012.
