# Supplementary material for: Jail, an unappreciated medical home: Assessing the feasibility of a strengths-based case management intervention to improve the care retention of HIV-infected persons once released from jail
Source: PLoS One. 2018 Mar 30;13(3):e0191643. doi: 10.1371/journal.pone.0191643 (PMC5877830; doi:10.1371/journal.pone.0191643)
Supplement: S4 File — (ZIP) [file pone.0191643.s004.zip › SUCCESS_Protocols_IRBApprovalLetters/SUCCESS_IRBProtocol_March2015.docx]

**Planning for SUCCESS (Sustained, Unbroken Connections to Care, Entry Services, and Suppression) – Phase II of a project to improve the connection to community care for HIV infected persons leaving jail in Atlanta.**

Principal Investigator’s Name:

Anne Spaulding MD MPH

Associate Professor, Department of Epidemiology

Rollins School of Public Health, Emory University

1518 Clifton Road NE, Room 3033

Atlanta, GA 30322

aspauld@emory.edu

(404) 727-3369

Co-Investigators: Carlos del Rio MD

Paula Frew PhD

Brent Johnson PhD

Sponsor: National Institute of Drug Abuse

Date: March 2, 2015

**1. Background**

**1.A. Specific aims of the study**

Each year, 1 in 7 persons with HIV in the U.S. enters a jail.[^1^](#_ENREF_1) Unimpeded access to care, with adequate support services to promote adherence to antiretroviral therapy, is key to reducing viremia and infectiousness. Dr. Spaulding, the PI of this proposal, has worked in jails for many years and has shown that jails represent a critical site for reaching individuals with no or sporadic HIV care and for delivering various public health interventions designed to enhance linkage to outpatient HIV care after release.[^2-9^](#_ENREF_2) Jails offer a unique point of interaction to seek and test those at high risk for HIV,[^10-13^](#_ENREF_10) identify new cases of HIV infection, and link the newly diagnosed to care.[^14^](#_ENREF_14) Jails also provide an opportunity to engage HIV+ individuals who are aware of their status but who are intermittently in care or whose care has been disrupted by incarceration. In the absence of a public health intervention, going to jail increases the odds 8-fold that HIV care will be disrupted.[^15^](#_ENREF_15)^,^[^16-19^](#_ENREF_16).

Public health interventions to improve engagement in care for HIV+ persons leaving jail are rare. Most interventions to help incarcerated HIV+ individuals have focused on those in prison, which are long-term correctional facilities; 95% of all U.S. inmates, however, pass only through jails, short–term facilities.[^1^](#_ENREF_1) Releasees of jails in Atlanta, GA, where this project will be based, navigate very complicated individual and systems-level barriers to link and stay in HIV care: one-third of these individuals are homeless or unstably housed, and most suffer from alcohol and/or drug dependence.[^20^](#_ENREF_20) When translated to the nation as a whole, potentially ~150,000 HIV+ releasees per year face similar obstacles to HIV care.[^1^](#_ENREF_1)^,^[^21^](#_ENREF_21)^,^[^22^](#_ENREF_22)

In response, we propose a feasibility study to develop data demonstrating that an innovative approach to intensive case management beginning in jail is acceptable and can promote the engagement in care of HIV+ jail releasees in the community. We want to build evidence for a manualized intervention, novel in its specificity for jail populations, which, if successful, can be scaled up and systematically applied to enhance linkage to HIV healthcare. To address the needs of this population, we will test the feasibility of an approach that builds on an empirically tested and now CDC-disseminated model[^23-25^](#_ENREF_23) of Strengths-Based Case Management developed in non-correctional settings. Randomized controlled trials have shown that Strengths-Based Case Management promotes lasting linkage of HIV+ persons to community-based HIV care, including access to long-term antiretroviral treatment.[^24^](#_ENREF_24)^,^[^25^](#_ENREF_25) Our adaptation of Strengths-Based Case Management for jails features wrap-around case management (i.e., spanning before and after jail discharge) to promote linkage and retention in HIV healthcare, substance abuse treatment, and mental health services.[^1^](#_ENREF_1)^,^[^26^](#_ENREF_26) The intervention is informed by extensive formative research, including focus groups which we have successfully conducted in preparation for this proposal. The feasibility study proposed in this application will explore how the jail setting and the post-release environment may facilitate or impede the systematic application of Strengths-Based Case Management. If the setting creates obstacles to the intervention, qualitative evaluation data gathered in this study will give insight into ways to mitigate the obstacles.

This proposed feasibility project, ***Planning for SUCCESS*** **(Sustained, Unbroken Connections to Care, Entry Services, and Suppression)**, will test the logistics and acceptance of an intervention and its evaluation tools against “usual care” conditions in preparation for a future randomized controlled trial. For the present study, we aim to explore if ***SUCCESS*** recipients will link to care at rates that match nationwide norms for the non-incarcerated. The specific aims of this proposal are:

**Aim 1. Demonstrate that recruitment and delivery of the intervention are feasible:** Recruit HIV+ jail detainees and deliver Strengths-Based Case Management in jail and during the first 60 days in the community, reinforced with cell phone contact upon release.

- *Hypothesis 1: HIV infected detainees can be recruite*d *at a pace of 14 per month on a bimonthly recruitment basis*.

**Aim 2. Connection to care:** Demonstrate that enrolled releasees will link to HIV care by 3 months post release as compared to those in usual care. A successful linkage to HIV medical care will be defined as a confirmed visit to a clinic post release, validated by a recorded HIV viral load and CD4 count in the clinic’s medical records.

- *Hypothesis 2: 75% of* ***SUCCESS*** *participants will link to HIV medical care by 3 months post release.*

**Aim 3. Establishment and retention in care:** Document retention in care; defined here as a minimum of 2 HIV clinical visits occurring within 12 months post release, with at least 2 clinical visits spaced a minimum of 3 months apart. Related retention outcome measures will include proportion with viral load suppression and, as needed, attendance at substance abuse rehabilitation and mental health treatment.

- *Hypothesis 3: 50% of* ***SUCCESS*** *participants will remain in HIV care at 12 months following release as compared to those in usual care.*

**1.B. Significance/justification for current study**

The ultimate goal of our research is to conduct a randomized control trial (RCT) of Strengths-Based Case Management (SBCM) for jail releasees versus usual care, coupled with a cost analysis. Most jails do not provide linkage services for HIV+ releasees; jurisdictions running jails are not convinced that providing such services merits the costs involved. However, if we showed this SBCM intervention to be efficacious, scalable, and cost-effective, then jails could provide a highly structured environment where the intervention might be delivered routinely to HIV+ persons. Our feasibility study will implement the protocol and gather preliminary data necessary for a power analysis before conducting an RCT and resulting cost analysis. We seek to contribute to the development of an evidence base for interventions to optimize the number of persons at the “fully engaged/retained” end of the HIV care spectrum among those leaving jails.[^27-30^](#_ENREF_27)

*** Rationale for using professional case managers to promote linkage:** Para-professional “peer” navigators have been used to help promote linkage of HIV+ persons to care in many settings.[^31^](#_ENREF_31) However, peers of inmates often have criminal records themselves; without passing a criminal background check, these peers cannot work in the criminal justice setting. As a result, an intervention using professional case managers (CMs) is more appropriate for a jail-based study.

*** The Strengths-Based Model:** Case management for HIV+ persons[^32^](#_ENREF_32) can employ different models. The two most common are the Broker model, where a CM assertively guides care, and the Strengths model, where a CM draws upon the strengths of the client.[^33^](#_ENREF_33)^,^[^34^](#_ENREF_34) The language of mental health rehabilitation, social work, and counseling (including offender rehabilitation) has incorporated the “Strengths-Based” model of concentrating on human potential rather than deficits by “looking at a glass as half-full rather than half-empty.”[^23^](#_ENREF_23)^,^[^33-35^](#_ENREF_33) Incorporating the concept of self-efficacy from Social Cognitive Theory,[^36^](#_ENREF_36) the Strengths-Based Case Management (SBCM) approach includes 3 principles: (i) establishing a trusting relationship with the CM, (ii) drawing on any past successes and a sense of self-efficacy in addressing current challenges (or barriers), and (iii) enhancing the participant’s sense of control in critical areas of their life. SBCM has consistently demonstrated its value in substance abuse treatment,[^37^](#_ENREF_37)^,^[^38^](#_ENREF_38) specifically in linking cocaine users with treatment[^39^](#_ENREF_39) and retaining them in aftercare.[^40^](#_ENREF_40)^,^[^41^](#_ENREF_41) SBCM has become one of the dominant models in HIV medical case management.[^32^](#_ENREF_32) SBCM without formative research was used in a 2-arm comparison trial among HIV+ North Carolina state prisoners: SBCM versus Broker-style, nurse-led discharge planning). This study showed statistically equivalent linkage between the two arms (65% and 54% respectively).[^42^](#_ENREF_42) Outcomes in both arms exceeded the anecdotally-reported, historical rate of 20% linkage (personal communication, D. Wohl).

*** Efficacy of SBCM in HIV care—the *ARTAS* study:** SBCM was the basis for *ARTAS* (Antiretroviral Treatment Access Study), a 4 city CDC-funded study which randomized recently diagnosed non-incarcerated HIV+ subjects to brief SBCM and care planning versus usual care. Dr. del Rio (co-investigator of this study) was PI for *ARTAS*’ Atlanta site. The outcome of interest was self-reported attendance (confirmed by medical record abstraction) at an HIV clinic twice in a 12-month period, an endpoint chosen because it was determined to be the minimum frequency of care required for any HIV+ person, even at early stages. Participants in the intervention arm received up to 5 sessions with a professional CM to facilitate linkage to HIV care. An important component of case management in *ARTAS* was that CMs and the client began by devising a plan to overcome barriers to HIV treatment, which included filling out paperwork for benefits and planning appointments for ancillary support services. Participants in the usual care arm received information about HIV and local care resources, along with a referral to a provider. Follow-up data were available on 91% of participants at 6 months and 86% at 12 months. Those who received the intervention were significantly more likely than controls to have had at least 1 HIV primary care visit in each of 2 consecutive 6-month follow-up periods. At 6 months follow up, 78% of participants receiving the intervention had linked to care, compared to 60% among controls. At 12 months follow up, 64% of subjects in the intervention arm had linked to *and were retained* in care compared to 49% in the control arm (RR(adj.): 1.41; p=0.006).[^24^](#_ENREF_24)

**Reproducibility of *ARTAS:*** An implementation study, *ARTAS*-2, followed the original *ARTAS* study and showed similarly high rates of linkage (79%).[^25^](#_ENREF_25) The CDC has selected *ARTAS* for national diffusion in the Diffusion of Effective Behavioral Interventions (DEBI) program; a procedures manual is available.[^43^](#_ENREF_43)^,^[^44^](#_ENREF_44)

*** Development of *SUCCESS*.** To modify *ARTAS* for the target population, Dr. Spaulding worked with behavioral scientist and Emory faculty member Dr. Wingood to implement her published protocol for adapting interventions, which modifies interventions that worked in one setting for use in another.[^45^](#_ENREF_45) Part of this adaptation involved holding focus groups with 4 groups of former jail detainees who were HIV+.

Focus group members judged that an adaptation of an *ARTAS*-like intervention specifically designed to engage jail releasees in care would be warranted. Complexities associated with their post-jail experiences suggested a need for an extra session of an *ARTAS*-like program and improved communication after release, a time of high mobility. Our study team decided to modify *ARTAS* by increasing its intensity and including cell phones to improve communication. Focus group participants advised having a multi-modal intervention (e.g., both case management and texting) to increase the likelihood of linkage—a multi-component approach that has also been recently suggested by expert panels.[^31^](#_ENREF_31)

**2. Design**

**2.A. Sample Population**

We anticipate recruiting 56 matched pair cases age 18 years or older, HIV infected, and who are incarcerated in Fulton County Jail or Atlanta City Detention Center. In the first month, we will recruit 2 study participants for whom we will pilot the intervention. Matching will be based on race/ethnicity, age, and gender. Based on our prior work, we anticipate that likely 85% of the participants will be male, 90% Black, and highest educational attainment will be less than a GED or high school diploma for 69%.

*** Safeguards in place for this vulnerable population:**

- Study will be introduced by usual jail healthcare staff; they will make referral of a potential subject to the study team only if the potential participant is interested and signs a release of information to permit forwarding of contact information to the study staff. Study staff will visit the jail and will speak with interested detainees referred by the healthcare staff.
- Informed consent process (see below) will let persons know benefits and risks of participating and that disenrollment will be possible at any step.
- Intervention will take place in settings where the study staff can maintain auditory and visual privacy.
- Study data (e.g., demographic survey, laboratory data) will be kept confidential.

*** Inclusion criteria:**

- HIV infected (HIV+); age of or over 18 years;
- Mentally able to give consent; understand spoken English;
- Detained or sentenced in either the Fulton County Jail or the Atlanta City Detention Center; and
- Likely to leave within 6 weeks.
- Able to read

*** Exclusion criteria:**

- Unable to give consent because of mental illness or inebriation;
- A recent participant in a randomized trial conducted by the investigators of an intervention to increase retention in HIV care (e.g., ARTAS)

**2.B. Setting**

Location of study procedures and/or data collection: Recruitment will occur in Fulton County Jail and Atlanta City Detention Center. Delivery of the first part of the intervention will be in this jail; the remaining steps will occur in the community, such as in the waiting rooms of HIV clinic offices or other locations convenient for the participants. Importantly, interventionalists will be available to meet with clients as needed including accompanying them to court The study staff will take care that confidentiality of health information will be maintained.

**2.C. Recruitment: site, procedure, methods for referrals, eligibility determination and enrollment**

i. REFERRALS

a) STANDARD REFERRAL METHOD: Jail healthcare staff will approach HIV positive inmates and ask if they would like to have study staff members talk to them about a potential project. If so, they will have potential participants sign a release of information form that will permit them to convey the name of the inmate to study staff. Jail healthcare staff will have a dedicated phone number to make participant referrals to the study staff.

b) ALTERNATIVE REFERRAL METHOD—SELF-REFERRAL: We will also recruit participants by distributing flyers to inmates in the housing unit dedicated to those with chronic medical conditions. Medical staff who distribute HIV medications and otherwise assist HIV infected detainee-patients will distribute flyers to HIV infected persons. The flyers will briefly describe the SUCCESS program goals and structure. Detainees interested in being contacted to participate in the study will be invited to write their booking number (which is written on each inmate’s wristband) on the back of the flyer and to submit the flyer to a member of the medical staff (*not* a correctional officer). Using a procedure employed by AIDS Service Organizations in the jail, the jail medical staff will deposit the slip in a secured area in a dedicated mailbox for the SUCCESS project. Medical staff will be instructed to fold the flyer such that the booking number is not visible on the outside. SUCCESS study staff will pick up the flyers and contact those inmates who submitted their booking numbers.

ii) ELIGIBILITY DETERMINATION: A member of the study staff will go to the jail to evaluate each potential participant’s eligibility for to participate in the study and explain the program. The study staff will begin an assessment by administering a REALM literacy test[^46^](#_ENREF_46) as used in *ARTAS*.[^47^](#_ENREF_47)^,^[^48^](#_ENREF_48) The study staff member will determine if the potential participant has a cell phone on the outside and inform him/her that study incentives will preferentially be paid in cell phone minutes.

Recruitment for the intervention arm will be conducted in waves of up to 20 participants, until an adequate sample size is achieved. Between periods of recruitment for the intervention group, we will recruit HIV infected detainees for a comparison group. Individuals recruited for the comparison group who undergo the informed consent process to participate as controls will receive a $10 gift card compensation mailed to an address of their choice.

All potential participants, regardless of whether they consent to either arm of participate in the study, will be given information about where to obtain HIV healthcare near where they plan to settle after release, such as a HRSA/ Ryan White supported clinic. Any written information on how to obtain benefits, medications, and substance abuse care will be written at a reading level of sixth grade or lower.

**2.D. Study Procedures**

**2.D.i. Study design**

- Study design is a longitudinal cohort study of individuals receiving a behavioral intervention. We will obtain baseline data (demographical data, psychological data, data extracted from jail medical chart review); deliver an intervention designed to improve linkage to medical care; and then administer surveys around months 3 (15 days before or up to 3 months after) and 12 (15 days before or up to 3 months after) to the participants after their release date. We will also conduct monthly check-ins with participants via text messaging to achieve a ≥ 85% retention rate at 12 months. Those who are recruited to the intervention and comparison condition will complete a consent form allowing the study team to extract data from medical records at the jail and, for those in the intervention group, at their HIV provider’s office in the community.
- The research material will be responses to self-report questionnaires, and abstracted data from participants’ medical charts. Records maintained at Emory University will be kept electronically on a strong password protected computer in a locked room. Each record will be assigned a unique code that is, in and of itself, non-identifying. The link to the code will be on a separate file without reference to HIV, and will be kept separately in a folder protected by a password known only to the PI and the project coordinator.
- Access to medical records of participants will be limited to those staff responsible for performing chart abstractions. Data from chart abstractions will be stored at Emory and will be identified only by a unique code.

CONTROLS

- Between the periods when we are recruiting participants who will receive the intervention, we will accept referrals of HIV infected jail detainees from the healthcare staff of the jail who can serve as controls. We will determine if the referred, potential subject matches a currently enrolled participant by gender and age, within five years above or below that of a participant who is receiving the intervention. We will verify HIV infection, either by past medical history or a rapid HIV test on site. If the potential control matches an already enrolled participant and is HIV-positive, the individual will be considered eligible to enroll as a control. We will then ask the person to undergo the informed consent process.
- We will continue the matching process until all currently enrolled participants are matched or a month has passed, whichever occurs first. If we are unable to find a suitable match we will ease the age requirement by two years to seven years above or below the age of the participant. In the event that there we are still unable to find a suitable match the age requirement will be eased by two more years to nine years above or below the age of the participant. We will then resume enrolling participants, 14-20 participants a month, one month at a time. When all 56 participants are enrolled, we will continue to recruit matched controls.

**2.D.ii. Data Collection Procedures**

The CM will begin an assessment by administering a REALM literacy test[^46^](#_ENREF_46) as used in *ARTAS*.[^47^](#_ENREF_47)^,^[^48^](#_ENREF_48) The CM will also determine if the study participant has a cell phone on the outside and will inform him or her that the study will ensure that all participants have cell phones. The CM will assess if the participant has mental health issues by administering a 3-minute Brief Jail Mental Health Screen.[^49^](#_ENREF_49)^,^[^50^](#_ENREF_50) Substance abuse disorder will be assessed using the Texas Christian University Drug Screen (TCUDS),[^51^](#_ENREF_51) and the World Health Organization’s Alcohol Use Disorders Identification Test (AUDIT). [^52^](#_ENREF_52) These will be administered verbally while giving instructions on how to use an audio/computer assisted survey instrument (ACASI)—these will constitute practice questions. The CM will stress that they will not have access to responses on the baseline evaluation, a 30-45 minute survey using ACASI which will include questions on substance abuse; if the verbal responses to the practice substance abuse screens were not quite truthful for any reason, the subjects can respond more honestly on the ACASI.

**2.D.iii.** **The Intervention**

The ***SUCCESS*** intervention that has been adapted for the target population is based on *ARTAS* (see 1.B.). The 4 core elements of *ARTAS* (1. developing working relationship with client, 2. identifying client’s goals, 3. encouraging client’s strengths, 4. meeting in the client’s environment) will be the core elements of ***SUCCESS***. Together, a CM and client will navigate through a fragmented and potentially confusing system of diverse resources and agencies. The philosophy of SBCM is to have clients set goals because, while the ultimate goal of ***SUCCESS*** is to link HIV+ persons to medical care, the subjects initially may not share this goal. The *ARTAS* intervention showed that, through engagement with CMs, clients were drawn to greater participation in medical care.[^24^](#_ENREF_24) The ***SUCCESS*** intervention will follow the objectives and strategies of the *ARTAS* intervention promoted by the CDC,[^44^](#_ENREF_44) with 3 adaptations: 6 rather than 5 *ARTAS*-like SBCM sessions, the use of mobile phones and texting, and the use of low literacy audio-visual aids in the sessions. The ***SUCCESS*** intervention will be delivered by the study’s 3 CMs, with the first 2 sessions (or “contacts”) designed to occur in jail, pre-release. Release dates in a jail can be unpredictable. If a study participant is released prior to completion of the first 2 contacts, those contacts will occur in the community. Subsequent contacts are designed to occur in the community post release and via text messaging, but they can take place in jail if release is delayed.

Figure. Client flow in ***SUCCESS*** intervention.


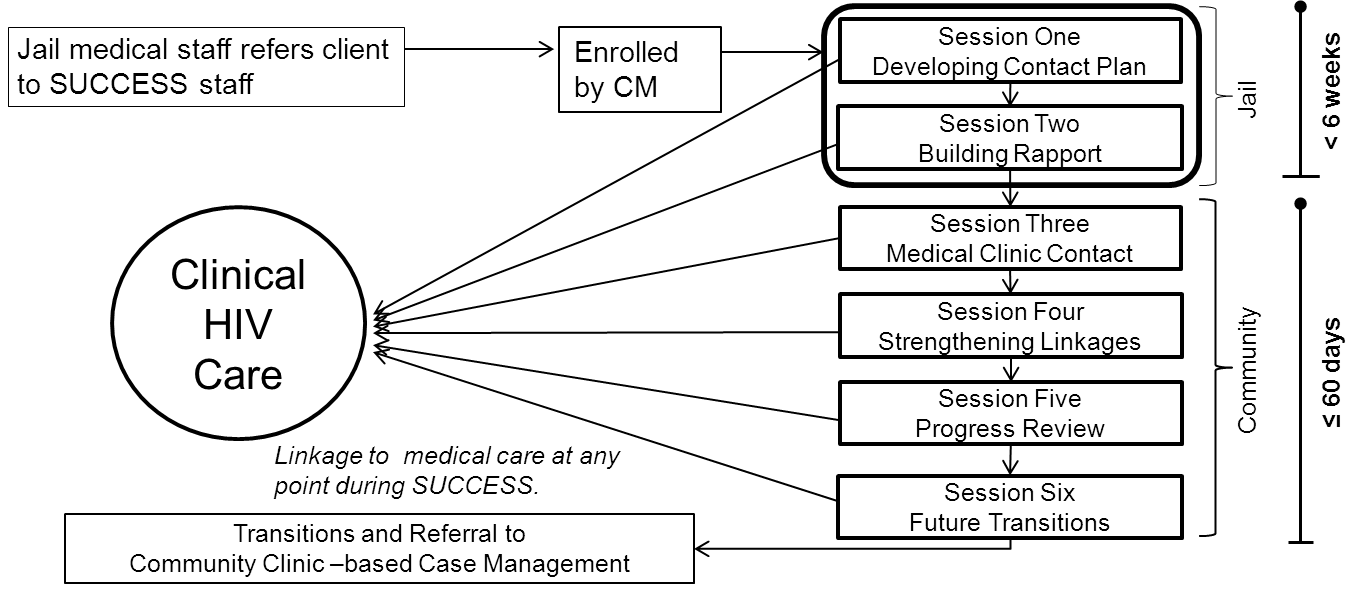


**Session 1** will begin with development of a “contact plan”, a systematic and structured plan to help a participant access medical services. A CM will spend ~1.5 to 2 hours face-to-face with a client for the provision of 3 initial services: 1) Completion of forms so that HIV drug benefits can be initiated or continued including AIDS Drug Assistance Program (ADAP) or indigent drug assistance programs, insurance and Medicaid forms, and forms to obtain a non-correctional picture ID and a birth certificate; 2) Making referrals if needs were identified during enrollment--to local substance abuse treatment programs if drug or alcohol dependency was identified as an issue based on the substance abuse screen, and to mental health care if the mental health screen was positive for serious mental illness; and 3) Confirming a participant’s choice of a clinic where high quality, specialty HIV care can be obtained, tailored to a participant’s needs or preferences. A participant can always choose later to change medical homes. The CM will also discuss how to arrange transportation and engage in services after jail discharge in this first session. The CM will also discuss plans to accompany participants to court visits. The philosophy of SBCM will be briefly introduced using low-literacy visual aids.

Rapport building and a personal strength assessment will take place at **Session 2** which will be ideally scheduled before the day of release. A key element of the intervention is the social support that will be offered to a participant, including having his or her CM meet a detainee at the jail’s exit door, since such meetings have been associated with improved linkage outcomes.[^7^](#_ENREF_7)^,^[^53^](#_ENREF_53) At the end of Session 2, a participant and his or her CM will decide where to meet upon release*.* Post-release sessions will ideally take place in a medical clinic (such as the Ponce Center or the AID Atlanta medical clinic—see letters of support) but can occur at another, mutually agreed upon, safe, public location, such as a coffee shop; public transportation to the meetings will be reimbursed. **Session 3** will occur around the time of a participant’s release from jail, preferably on the day of release. It will focus on reviewing how to make contact with the chosen medical clinic if no medical visit has yet occurred. In addition, a CM will confirm that a participant has access to a cell phone. Many releasees will have personal cell phones; we will provide basic cell phones for those without mobile phones at the first outside visit either with the CM or evaluation team post release. A CM will encourage a participant to set reminders on his or her phone to assist with keeping appointments or taking medications. Unless a participant opts out, his or her CM will arrange for the delivery of both automated and personalized messages to help with the achievement of goals.[^54^](#_ENREF_54) From the focus groups, we learned releasees may want text messages to remind them of clinic appointments, to hold them accountable in challenging situations such as dealing with negative peer pressure, to serve as periodic check-ups for an update on status, and for general words of encouragement. We will tailor these messages to a participant’s stated needs and literacy level. For example, illiterate participants may receive text-to-phone audio messages. The study team will initiate text message once per week for the 60 days of the post-release period. We will contract with a specialized text messaging vendor, who will help push out messages at pre-designated intervals. Transmission times can be individualized for the recipients, based on work schedules, rehabilitative therapy, etc. Our contracted partner will also receive text messages, such as responses concerning adherence, and will store the replies in their centralized database for retrieval.

**Session 4** will focus further on linkage to services that can reduce risk in problematic areas, such as treatment for substance abuse that hinders medication adherence or mental health treatment that might interfere with maintaining employment. (St. Jude’s Recovery Center, Positive Impact, and other agencies have signed support letters indicating their willingness to take referrals of ***SUCCESS*** participants). With the help of his or her CM, a participant will map out the steps needed to gain entry into treatment programs.

At **Session 5,** a participant and his or her CM will review progress on how well the contact plan was achieved. **Session 6** will focus on disengagement and transition to the clinic’s usual case management. A typical participant will receive 6 sessions, all of which should be completed within the first 60 days post release. Missed sessions can be rescheduled, as soon as is possible. After the 6 initial sessions, a participant may informally contact his or her CM by phone or in person for review of the contact plan, but if more intense case management is needed, the study CM will make a referral to case management through a participant’s medical home. Although sessions 1 and 2 are designed to be delivered in jail and sessions 3 through 6 in the community, the program includes the necessary flexibility so that most sessions could be delivered in or out of the jail setting. This is necessary given the unpredictability of jail release dates and the potential for re-incarceration. Text messaging and phone calls to a CM will occur throughout the period of SBCM delivery.

**2.D. iv. Total Respondent Burden**

The baseline demographic data should take 30-45 minutes to complete. The intervention will involve 6-12 hours of face time with a case manager. The subsequent surveys, at 3 and 12 months, should each take about 30 minutes to complete. Responding to text messages should take minimal time to complete.

**2.E. Measures**

*** Process measurement**

Dr. Frew, assisted by Ms. Bowden, will oversee evaluation of the CMs. We anticipate the 3 CMs will each be assigned 4-5 new participants per month (a total of 14 assigned per month); participants will stay in the “active phase” for 2 months after discharge. At bi-weekly meetings, Ms. Bowden will review data on: (1) the number of intervention sessions that each participant receives, and (2) any make-up sessions if initial visits are missed. She will adjust the assignment of participants to CMs so that there is balance between the caseloads.

With permission of the participant, Ms. Bowden will periodically observe sessions and afterwards discuss her observations with the CMs. Independently, the CMs will collect information documenting the fidelity with which ***SUCCESS*** is implemented. CMs will document each contact with participants on their caseload, recording specific information about a session including: date, location and length of contact, presence of other persons or not, participant use of cell phones, referrals to clinical and social services that are followed through and intensity of referral (e.g., phone call only or went with participant to referral), and court appearances. Further, CMs will fill out a brief form that will permit a listing of what barriers to linking with medical care were discussed during each session.

Dr. Frew will implement independent observation of staff-participant interactions. Using a random number generator to select sessions, we will audiotape a random sample of 10% of sessions in the community (current jail policy precludes audiotaping in the facility). We will also capture responses given to text messaging prompts to track factors associated with retention. Dr. Frew and Project Coordinator Bowden, assisted by Dr. Rapp, will monitor the conduction of the intervention activities by reviewing the audio-taped sessions on a weekly basis. The basis for these observations will be the *ARTAS*-like Case Management Fidelity Scale consisting of 5 areas that are central to the delivery of strengths-based interventions such as ***SUCCESS***. These areas include: focus on strengths, encouragement of participant-driven activities, relationship building, assertive outreach, and use of resources. Auditors will rate CMs on 29 specific tasks in the 5 areas using a 5 point Likert-like scale that asks whether auditors Disagree Strongly (1) to Agree Strongly (5) that a CM has delivered a task with full fidelity. Ms. Bowden will provide feedback to CMs based on their scores on the fidelity scale. Prompt discussion of fidelity scores has been demonstrated to be an effective tool in improving adherence to intervention protocols.[^55^](#_ENREF_55) Additionally, the CMs will present cases at bi-weekly supervisory sessions. These sessions will allow Ms. Bowden the opportunity to monitor adherence to ***SUCCESS***, provide a teaching opportunity in areas she feels need additional attention, address and resolve any problems that may arise during the intervention, and ensure fidelity to the protocol.

*** Outcomes measurement**

The primary outcome will be whether a participant linked to community HIV care and, if it occurs, how long before a linkage occurred since the initial release from jail. Linkage to care will be defined as a visit to an HIV provider that entails the acquisition of an HIV viral load and CD4 cell count. We will hire a staff Research Assessor (RA, to be named), who will be assisted by part-time graduate students to serve as the assessment team. All will be trained in phlebotomy. A member of the assessment team will have an introductory meeting with a participant within 30 days of release. Additionally, an assessor will gather extensive psychosocial data at 3- and 12-month assessments and will collect an HIV viral load at month 12 on those participants in the intervention group not being followed by an HIV medical provider.

We will submit the list of controls to the state department of public health. The state will inform us what percentage had a CD4 count, viral load, or both drawn in the 12 months following release from their index incarceration. They will tell us what percentage of releasees had detectable viral loads and the range of viral loads of those with detectable virus. We will compare the outcomes of those with the intervention versus the controls.

The state department of public health (Georgia Department of Public Health) also maintains a death registry which they use to periodically update their electronic HIV /AIDS Reporting System (eHARS) used for the check of HIV labs—the two offices are in close communication. In order to determine whether any participants and controls are not linked to care due to death since the last periodic update of the HIV registry, we will submit a list of names of study subjects to the Death Registry of the Georgia Department of Public Health. The names will be on a password protected encrypted spreadsheet. The Georgia Department of Public Health will report to us the vital status of the individuals on the list during the one year follow-up period.

**2.F. Risks to Participation**

Potential discomfort or risk to a subject includes 1) psychological distress if a participant feels coerced to participate; 2) breach of confidentiality of health information; and 3) participants who are not linked to HIV primary care at 12 months will be asked to undergo a blood draw for the study, which carries risk of pain and bruising at the site of venipuncture. This research does not adversely affect a participant’s rights and welfare, provided that the confidentiality of identifying information is maintained. The potential risks listed above will be addressed in the following manner:

1. Subjects may experience psychological stress if they feel coerced to participate. However, the likelihood of this risk is small. When the study team solicits informed consent, potential participants will be told that their enrollment is voluntary. Furthermore, enrolling in the study will not affect the conditions of any parole or probation. Participant incentives in this study are nominal and will be given to participants only after reentry to the community. Participants may separate from the study at any point without any penalty.
2. To minimize the risk of a breach of confidentiality of any medical data (including HIV status and other sensitive information), participants will be assigned study ID numbers. These numbers, rather than personally-identifiable information, will be associated with data. Confidential information, including health information, will be stored in a locked drawer in a locked study office. Only study staff will have access to the keys. The audio recordings of the focus group and text message logs will be destroyed at the completion of the study. Standards for privacy of individually identifiable health information under the Health Insurance Portability and Accountability Act of 1996 will be met. HIPAA Authorization documentation will be incorporated into the consent forms that will be obtained from participants. The only information that will not be kept confidential is if volunteers or members of the study staff believe that program participants are a danger to themselves or others; or if minor children are under control of the participant, and there is reasonable evidence that the children are at risk.
3. Phlebotomy—for those participants who are not in HIV primary care at 12 months and have no current HIV associated laboratory studies from an HIV clinic, a separate consent form for a blood draw will be administered. The evaluation staff drawing blood will be trained in proper technique for phlebotomy, to minimize pain and bruising that can be associated with the procedure.

**2.G. Benefits to Subject or further benefits**

The findings of this study will help inform future adaptations of the intervention to jails in urban settings. These future adaptations could help inform policy makers of structural interventions to improve linkage to care for HIV-positive jail releasees. Population subgroups disproportionately affected by incarceration will be the greatest beneficiaries. In addition to receiving a small cash incentive, individual study participants may benefit from improved linkage to care, as facilitated by the SBCM intervention sessions.

**2.H. Data Analysis**

**Rationale for proposed number of subjects:**

No sample size calculations have been performed because this pilot study has not been powered to achieve outcomes that are statistically significant. We have chosen a sample size of 56 matched pair cases because it is a reasonable number of persons to pilot the intervention/new protocol, SUCCESS, to show feasibility.

*** Management of quantitative data.**

We will hire a part-time data manager. With her/his help, we will develop ACASI forms for data collection, using templates already designed for other STTR-CJ studies. We will use REDCap, a Data Management System with a low-priced user’s license fee, for electronic storage of data. Emory’s Health Science Information Technology Office will work with the team to develop a password-protected project website. The website will have a portal to the Data Management System which will accept electronic data downloaded from the ACASI surveys. The data manager can help the study team design appropriate validations in REDCap and ensure data integrity and quality using SAS. With the help of the data manager, the study staff will be able to retrieve interim data and summary and other information related to the study data when requested.

At the request of our funder, we plan to share our *deidentified* data with researchers at the University of Washington participating in the STTR-CJ Data Harmonization project. The purpose of this project is to increase the comparability, collaboration, and scientific yield of clinical research on HIV and drug abuse by combining data from multiple sites. No PHI will be included.

*** Analytic plan for Aims.**

Dr. Brent Johnson will assist Dr. Spaulding with the analysis of quantitative data. As in *our* previous studies, we estimate that attrition due to transfer to prison will occur in 10% of the 56, leaving about 50 persons whose data can be analyzed and summarized in comparison to an equal number of selected matched cases. Because of the small sample size in this preliminary feasibility study, our statistical analysis plan will consist of simple descriptive statistics of primary study endpoints and their unadjusted associations with clinical or psychosocial variables. **(AIM 2) *Connection to Care*** will be measured in 2 ways, first as the time between the day of release from the index incarceration and the first day the participant kept an appointment in an HIV clinic in which a laboratory draw is completed. The time to linkage can be displayed as a Kaplan-Meier curve, with the understanding that time to linkage may be right-censored if it did not occur by the release date. The second analysis will treat linkage as a dichotomous variable (participant linked/failed to link). We will explore the association of variables relevant to linkage, via univariate logistic regression. **(AIM 3)  *Retention in care over one year*:** The intervention will be considered successful for a participant if the participant has at least 1 HIV clinic visit in the 3 months post-initial discharge as well as a 2 additional HIV clinic visits, spaced at least 3 months from the first visit post release. The subsequent clinic visits may occur either outside or inside of a jail (if re-incarcerated). Based on previous studies we anticipate that 10% of clients will go to prison or immigration customs enforcement and be disenrolled. We anticipate that from the 50 remaining jail releasees (out of the 56 original enrollees) another 6 persons will be lost to follow up. We will perform univariate logistic regression to assess the influence of variables on these 44 remaining participants and their selected matched comparison cases.

*** Power calculation**

For the exploratory analysis, for variables that split the pool of 44 into groups of 22 each, we will have 48% statistical power to detect difference in retention of 60% versus 80%; we will have 82% statistical power to detect differences between 60% and 90%. For example, if roughly half of the participants had benefits for medical coverage, and having benefits had a large effect size, we would be able to describe if having such medical benefits significantly increased retention. Otherwise, if the variable does not split the subject pool in two or did not have a large effect size, we can describe trends but not be able to comment on significance in our exploratory data analysis. We will perform a similar analysis to explore associations with viral suppression. Since not all participants will have referral to substance abuse and mental health treatment, we will use simple frequencies and descriptive statistics to summarize the marginal distribution of service utilization for these specific services; we anticipate insufficient power to calculate statistical significance of associations. We will count the number of contacts with CMs, mental health providers, and drug treatment sessions. In the ultimate RCT of *SUCCESS*, these may be analyzed as mediators for linkage and retention. In this proposed feasibility study, we will only investigate potential mediating and moderating relationships through descriptive statistics.

**3. Training**

Dr. Rapp has conducted 2 days of face-to-face training in SBCM with Project Coordinator Ms. Bowden and another study staff member, and will similarly train the newly hired study personnel to ensure that they can implement the intervention with fidelity to the conceptual model and the protocols. At the end of the training period, all intervention staff will demonstrate comprehension of the 3 principles of SBCM (detailed in 1.B.) and how they relate to the predisposing, enabling, and need-based factors inherent in the conceptual model. CMs will have refresher training sessions once per quarter. All CMs will also receive training in administration of the informed consent process and the initial baseline evaluation. Having the intervention staff, rather than a separate, independent assessment team used for later phases of evaluation, administer the baseline survey is a concession that reflects the complexity of having multiple staff members connect with participants during the short period that they are the jail. We will train the CM staff and the independent assessment team to a standard so that anyone who administers the assessments will do so uniformly.

Dr. Frew will train the members of the assessment team to ensure that they understand the study purpose, their role in the study, how to administer the ACASI surveys with participants, (a brief survey immediately post release, then half-hour surveys at months 3, 6, and 12) and how to complete all other data collection instruments. Staff and student assessors will be trained to deliver scripted interactions with participants without leading them. Dr. Spaulding will train assessors in medical chart abstraction. Issues regarding staff safety will be reviewed. The RA and student assessors will have refresher training sessions once per quarter.

**4. Plans for Data Management and Monitoring**

Because this study is a preliminary feasibility study, without a control group, we will not have a formal Data Safety and Monitoring Board. If participants fail to link to and stay in care at greater than historical rates, we will not conduct a future randomized trial of the intervention.

**5. Confidentiality**

**5.A. Plans to protect privacy of subjects and confidentiality of data and link data to identifiers**

All participant information will be kept private. Upon enrollment in the study, the participant will be assigned a study identification number to which all information will be tracked. Text message logs will also be linked to participant study IDs.

**5.B. How linkage will be protected**

Participant information will be stored in locked cabinets and on password protected computers. Access to the project data management system and text messaging data will only be given study staff/researchers with individual, private logins and passwords.

**6. Informed Consent**

Upon successful completion of an eligibility assessment, study participants will be asked by study staff to participate in the study. Study participants will be given a written Informed Consent Form which will be read to them by the study staff person at which time if the participants agree, will sign. Study participants may opt out of the study at any time. Site staff will be instructed not to recruit inebriated inmates. Once enrolled into the study, participants will be assigned to the case manager who will provide case management services.

As of March 2015, participants who have been released from jail may be given a chance for future opportunities. At the discretion of the case manager, participants may be given the opportunity to provide consent to be contacted for participation in future studies. Chosen study participants will be given a written informed consent forms corresponding to this reason for contact outside of normal study procedures. The consent form will be read to them by the study staff. If the participant agrees to be contacted, they will sign the corresponding form. Participants will provide a range of dates on the consent form indicating when contact may begin and when they no longer consent to be contacted. Participants may revoke their consent to be contacted at any time and not suffer any consequences.

**7. Plans to Inform Participants of New Findings**

The study researchers will disseminate findings from this study in both scientific and lay formats. The study staff persons or community base partners will attempt to contact clients about results that might affect their health.

**REFERENCES**

**1.** Spaulding AC, Seals RM, Page MJ, Brzozowski AK, Rhodes W, Hammett TM. HIV/AIDS among inmates of, and releasees from, US correctional facilities, 2006: declining share of epidemic but persistent public health opportunity. *PLoS ONE [Electronic Resource].*4(11):e7558.

**2.** Draine J, Ahuja D, Altice FL, et al. Strategies to enhance linkages between care for HIV/AIDS in jail and community settings. *AIDS Care.* 2011;23(3):366-377.

**3.** Spaulding AC, Arriola KR, Ramos KL, et al. Enhancing linkages to HIV primary care in jail settings. *Journal of Correctional Health Care.* 2007;12(2):93-128.

**4.** Spaulding AC, Booker CA, Freeman SH, et al. and The EnhanceLink Study Group. Jails, HIV testing and linkage to care services: an overview of the EnhanceLink project. *AIDS and Behavior (in press).*

**5.** Spaulding AC, Messina LC, Kim BI, et al. Planning for Success Predicts Virus Suppressed: Results of a Non-Controlled, Observational Study of Factors Associated with Viral Suppression among HIV-positive Persons Following Jail Release. *AIDS and Behavior (in press).*

**6.** Stein MS, Spaulding AC, Cunningham M, et al. HIV-positive and in jail: race, risk factors, and prior access to care. *AIDS and Behavior (in press).*

**7.** Booker CA, Flygare CT, Solomon L, et al. and The EnhanceLink Study Group. Linkage to HIV care for jail detainees: findings from the first 30 days after release. *AIDS and Behavior (in press).*

**8.** Chen N, Meyer J, Avery A, et al. Adherence to HIV treatment and care among previously homeless jail detainees. *AIDS and Behavior.* 2011:1-13.

**9.** Spaulding AC, Pinkerton S, Superak H, et al. Cost Analysis of Enhancing Linkages to HIV Care Following Jail: A Cost-Effective Intervention. AIDS and Behavior.(in press).

**10.** Spaulding A, Stephenson B, Macalino G, Ruby W, Clarke JG, Flanigan TP. Human immunodeficiency virus in correctional facilities: a review. *Clinical Infectious Diseases.* Aug 1 2002;35(3):305-312.

**11.** Wohl D, Rosen D, Kaplan A. HIV and incarceration: dual epidemics. *The AIDS Reader.* 2006;16(5):247-250, 257-260.

**12.** MacGowan RJ, Margolis AD, Richardson-Moore A. Voluntary rapid HIV testing in jails. *Sexually Transmitted Diseases* 2009;36(suppl 2):s9-s13.

**13.** Spaulding AC, Arriola KRJ, Hammett T, Kennedy S, Tinsley M. Rapid HIV testing In rapidly released detainees: next steps. *Sexually Transmitted Diseases* 36(suppl 2):s34-s36.

**14.** DeVoux A, Beckwith C, Avery A, et al. Early Identification of HIV: Empirical Support for Jail-Based Screening. *PLoS ONE [Electronic Resource].* 2012;7(5):e37603.

**15.** Westergaard RP, Kirk GD, Richesson DR, Galai N, Mehta SH. Incarceration Predicts Virologic Failure for HIV-Infected Injection Drug Users Receiving Antiretroviral Therapy. *Clinical Infectious Diseases.* 2011;53(7):725-731.

**16.** Stephenson BL, Wohl DA, Golin CE, Tien H-C, Stewart P, Kaplan AH. Effect of release from prison and re-incarceration on the viral loads of HIV-infected individuals. *Public Health Reports.* Jan-Feb 2005;120(1):84-88.

**17.** Springer SA, Pesanti E, Hodges J, Macura T, Doros G, Altice FL. Effectiveness of antiretroviral therapy among HIV-infected prisoners: reincarceration and the lack of sustained benefit after release to the community. *Clinical Infectious Diseases.* Jun 15 2004;38(12):1754-1760.

**18.** Baillargeon J, Giorgano TP, Harzke AJ, et al. Reincarceration and HIV progression in released prisoners. *AIDS Patient Care and STDs (in press).*

**19.** Clements-Nolle K, Marx R, Pendo M, et al. Highly active antiretroviral therapy use and HIV transmission risk behaviors among individuals who are HIV infected and were recently released from jail. *American Journal of Public Health.* Apr 2008;98(4):661-666.

**20.** Stein MS, Spaulding AC, Cunningham M, et al. HIV-Positive and in Jail: Race, Risk Factors, and Prior Access to Care. *AIDS and Behavior (in press).* 2012.

**21.** Maruschak LM, Beavers R. HIV in prisons, 2007-8. *Bulletin of the Bureau of Justice Statistics.* 2009. <http://bjs.ojp.usdoj.gov/content/pub/pdf/hivp08.pdf>. Accessed 01/16/2010.

**22.** Harrison PM, Beck AJ. Prison and jail inmates at midyear 2005. *Bulletin of the Bureau of Justice Statistics.* 2006. <http://bjs.ojp.usdoj.gov/content/pub/pdf/pjim05.pdf>. Accessed 01/02/2007.

**23.** Rapp CA, Goscha RJ, eds. *The strengths model: case management with people with psychiatric disabilities, 2nd ed* New York City: Oxford University Press 2006.

**24.** Gardner LI, Metsch LR, Anderson-Mahoney P, et al. Efficacy of a brief case management intervention to link recently diagnosed HIV-infected persons to care. *AIDS.* Mar 4 2005;19(4):423-431.

**25.** Craw JA, Gardner LI, Marks G, et al. Brief strengths-based case management promotes entry into HIV medical care: results of the antiretroviral treatment access study-II. *JAIDS.* Apr 15 2008;47(5):597-606.

**26.** Flanigan TP, Zaller N, Beckwith CG, et al. Testing for HIV, Sexually Transmitted Infections, and Viral Hepatitis in Jails: Still a Missed Opportunity for Public Health and HIV Prevention. *JAIDS.* 2010;55:s78-s83.

**27.** Malitz FE, Eldred L. Evolution of the special projects of national significance prevention with HIV-infected persons seen in primary care settings initiative. *AIDS & Behavior.* Sep 2007;11(5 Suppl):S1-5.

**28.** Cheever LW. Engaging HIV-infected patients in care: their lives depend on it. *Clinical Infectious Diseases.* 1500;44(11):1500-1502.

**29.** Gardner EM, McLees MP, Steiner JF, Del Rio C, Burman WJ. The spectrum of engagement in HIV care and its relevance to test-and-treat strategies for prevention of HIV infection. [Review]. *Clinical Infectious Diseases.* 2011;52(6):793-800.

**30.** Fleishman JA, Yehia BR, Moore R, Korthuis PT, Gebo KA, For the HIV Research Network. Establishment, retention, and loss to follow-up in outpatient HIV care. *JAIDS.* 2012;60(3):249-259.

**31.** Thompson MA, Mugavero MJ, Amico KR, et al. Guidelines for Improving Entry Into and Retention in Care and Antiretroviral Adherence for Persons With HIV: Evidence-Based Recommendations From an International Association of Physicians in AIDS Care Panel. *Annals of Internal Medicine.* 2012;156(11):817-833.

**32.** Fleisher P, Henrickson M. Towards a Typology of Case Management. <ftp://ftp.hrsa.gov//hab/Typology.pdf>. Accessed 02/01/2010.

**33.** Healey KM. Case management in the criminal justice system- reseach in action. 1999. <http://www.ncjrs.gov/pdffiles1/173409.pdf>. Accessed 03/20/2010.

**34.** Weick A, Rapp C, Sullivan WP, Kisthardt W. A Strengths Perspective for Social Work Practice. *Social Work.* 1989;34(4):350-354.

**35.** Ward T, Brown M. The good lives model and conceptual issues in offender rehabilitation. Routledge2004.

**36.** Bandura A. Self-efficacy:toward a unifying theory of behavioral change. *PsychRev.* Mar 1977;84(2):191-215.

**37.** Vaughan-Sarrazin MS, Hall JA, Rick GS. Impact of case management on use of health services by rural clients in substance abuse treatment. *Journal of Drug Issues.* 2000;30(2):435-463.

**38.** Strathdee SA, Ricketts EP, Huettner S, et al. Facilitating entry into drug treatment among injection drug users referred from a needle exchange program: Results from a community-based behavioral intervention trial. *Drug Alcohol Depend.* Jul 27 2006;83(3):225-232.

**39.** Rapp RC, Otto AL, Lane DT, Redko C, McGatha S, Carlson RG. Improving linkage with substance abuse treatment using brief case management and motivational interviewing. *Drug Alcohol Depend.* Apr 1 2008;94(1-3):172-182.

**40.** Rapp RC, Siegal HA, Li L, Saha P. Predicting postprimary treatment services and drug use outcome: a multivariate analysis. *Am J Drug Alcohol Abuse.* Nov 1998;24(4):603-615.

**41.** Siegal HA, Li L, Rapp RC. Case management as a therapeutic enhancement: impact on post-treatment criminality. *J Addict Dis.* 2002;21(4):37-46.

**42.** Wohl D, Scheyett A, Golin C, et al. Intensive Case Management Before and After Prison Release is No More Effective Than Comprehensive Pre-Release Discharge Planning in Linking HIV-Infected Prisoners to Care: A Randomized Trial. *AIDS and Behavior.* 2011;15(2):356-364.

**43.** Stoll P, Duncan T, Marr O. The Diffusion of ARTAS: What CDC Plans to Do. Paper presented at: CDC National HIV Prevention Conference; August 14-17, 2011; Atlanta GA.

**44.** Anti-Retroviral Treatment and Access to Services (ARTAS) An individual-level, multi-session intervention for people who are recently diagnosed with HIV Implementation Manual. Published May 2011. Available: <http://www.cdc.gov/hiv/topics/cba/pdf/artas_implementation_manual.pdf> Accessed: 20 August 2011.

**45.** Wingood GM, DiClemente RJ, Wingood GM, DiClemente RJ. The ADAPT-ITT model: a novel method of adapting evidence-based HIV Interventions. *JAIDS.* Mar 1 2008;47 Suppl 1:S40-46.

**46.** Davis TC, Long SW, Jackson RH, et al. Rapid estimate of adult literacy in medicine: a shortened screening instrument. *Family Medicine.* 1993;25(6):391-395.

**47.** Barragan M, Hicks G, Williams MV, Franco-Paredes C, Duffus W, del Rio C. Low health literacy is associated with HIV test acceptance. *Journal of General Internal Medicine.* 2005;20(5):422-425.

**48.** Hicks G, Barragan M, Franco-Paredes C, Williams MV, del Rio C. Health literacy is a predictor of HIV/AIDS knowledge. *Family Medicine.* 2006;38(10):717-723.

**49.** Steadman HJ, Scott JE, Osher F, Agnese TK, Robbins PC. Validation of the Brief Jail Mental Health Screen. *Psychiatric Services.* 2005;56(7):816-822.

**50.** Brief Jail Mental Health Screen. <http://gainscenter.samhsa.gov/topical_resources/bjmhs.asp>. Accessed 08/18/2012.

**51.** TCU Drug Screen II. 2006; <http://www.ibr.tcu.edu/pubs/datacoll/Forms/ddscreen-95.pdf>. Accessed 06/27/2011.

**52.** Boutwell A, Rich JD. HIV infection behind bars.[see comment][comment]. *Clinical Infectious Diseases.* Jun 15 2004;38(12):1761-1763.

**53.** Jacob Arriola KR, Braithwaite RL, Holmes E, Fortenberry RM. Post-release case management services and health-seeking behavior among HIV-infected ex-offenders. *Journal of Health Care for the Poor & Underserved.* Aug 2007;18(3):665-674.

**54.** Dunbar PJ, Madigan D, Grohskopf LA, et al. A two-way messaging system to enhance antiretroviral adherence. *Journal of the American Medical Informatics Association.* 2003;10(1):11-15.

**55.** Carroll KM, Nich C, Sifry R, et al. A general system for evaluating clinician adherence and competence in psychotherapy research in the addictions. *Drug and Alcohol Dependence.* 2000;57:225-238.
